# Naphtho-Gamma-Pyrones (N*γ*Ps) with Obvious Cholesterol Absorption Inhibitory Activity from the Marine-Derived Fungus *Aspergillus niger* S-48

**DOI:** 10.3390/molecules27082514

**Published:** 2022-04-13

**Authors:** Chang-Zheng Wu, Xiao-Ping Peng, Gang Li, Qi Wang, Hong-Xiang Lou

**Affiliations:** 1 Department of Natural Medicinal Chemistry and Pharmacognosy, School of Pharmacy, Qingdao University, Qingdao 266021, China; 2019026576@qdu.edu.cn (C.-Z.W.); pengxiaoping@qdu.edu.cn (X.-P.P.); gang.li@qdu.edu.cn (G.L.); wangqi@hmfl.ac.cn (Q.W.); 2 Key Laboratory of Chemical Biology of Ministry of Education, Department of Natural Product Chemistry, School of Pharmaceutical Sciences, Shandong University, Jinan 250012, China

**Keywords:** *Aspergillus niger*, natural products, naphtho-gamma-pyrones (N*γ*Ps), Niemann-Pick C1-Like 1, cholesterol

## Abstract

Eight naphtho-gamma-pyrones (N*γ*Ps) (**1**–**8**), together with four known biosynthetically related coumarin derivatives (**9**–**12**), were isolated from the potato dextrose agar media of a marine-derived fungus *Aspergillus niger* S-48. Among them, natural compounds **1** and **2** were tentatively subjected to benzohydrazide reaction to evaluate the importance of pyran rings in N*γ*Ps. Their structures were elucidated by extensive 1D and 2D NMR spectroscopic data and MS spectra. Compounds **1**–**4** showed obvious activity for reducing cholesterol absorption verging on ezetimibe. This work highlighted the potential of natural N*γ*Ps as NPC1L1 inhibitors.

## 1. Introduction

Marine fungi with unique metabolic mechanisms under hypersaline, hyperbaric, and oligotrophic conditions have provided structurally diverse and pharmacologically active secondary metabolites [1,2,3]. Among them, *Aspergillus niger* was one of the most-found marine fungal species [4], and biosynthesized a number of specialized small molecules, such as naphtho-gamma-pyrones (N*γ*Ps), ochratoxins, fumonisins, bicoumarins, malformins, and asperazines [5]. Particularly, N*γ*Ps pigments were extensively isolated as antimicrobial, antiviral, and antioxidant agents, attracting a lot of attention from chemists and biologists [6,7,8,9,10].

**Figure 1:**
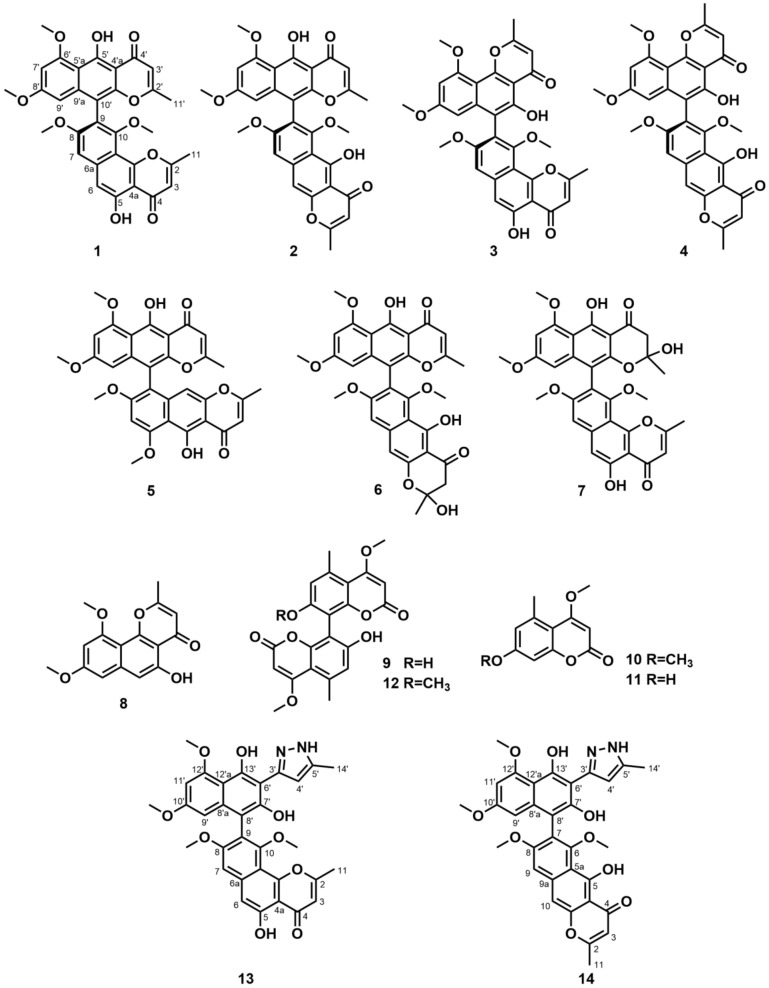
Structures of compounds **1**–**14**.

In our continuing isolation on marine-derived fungi [3], an *Aspergillus* species, *A. niger* S-48, was obtained, and was subjected to chemical investigation to pursue biologically active N*γ*Ps pigments. As expected, twelve secondary metabolites (Figure 1), including eight known N*γ*Ps, were finally isolated from *A. niger* S-48. Following the established bioassay approaches in our laboratory, these isolates were evaluated for antibacterial, antifungal, cytotoxic, and quorum-sensing inhibitory activity, and cholesterol absorption inhibition activities. In order to tentatively evaluate the importance of pyran ring in N*γ*Ps for bioactivity, two compounds with enough amounts were modified for bioassay. Herein, the detailed isolation, identification, and bioactivities of natural or semisynthetic compounds are described.

## 2. Experimental Section

### 2.1. General Experimental Procedures

HRESIMS data were obtained on an LTQ-Orbitrap spectrometer (Thermo Fisher Scientific, Waltham, MA, USA) equipped with an ESI source. NMR spectra were measured on Bruker Advance 500 MHz and JEOL JNM-ECP 600 MHz spectrometers. Optical rotations were measured on a PerkinElmer 241MC polarimeter (PerkinElmer Instruments, Norwalk, CT, USA) in MeOH at 20 °C. Electronic circular dichroism (ECD) spectra were acquired on a Chirascan spectropolarimeter (Applied Photophysics, Leatherhead, UK). Column chromatography (CC) was equipped with silica gel (200−300 mesh; Qingdao Haiyang Chemical Co., Ltd., Qingdao, China). Thin-layer chromatography (TLC) was performed with silica gel GF_254_ plates (Qingdao Haiyang Chemical Co., Ltd., China). Flash chromatography was performed on a Teledyne ISCO CombiFlash Rf 200 system equipped with a C18 spherical column (20–35 μm, 100 Å, 80 g). The semi-preparative high-performance liquid chromatography (HPLC) system (Agilent 1260 Infinity II; Agilent technologies, Böblingen, Germany) was equipped with a 1260 Quat Pump VL, a 1260 Vialsampler, a 1260 MCT, a 1260 DAD WR, and a ZORBAX SB-C18 column (5 µm, 9.4 × 250 mm), a C18 YMC-Pack ODS-A column (5 μm, 10.0 × 250 mm), and a *π* NAP COSMOSIL Packed Column (5 μm, 10.0 × 250 mm).

### 2.2. Strain and Culturing Conditions

The fungal strain *A. niger* S-48 was isolated from the root of the mangrove plant, *K. candel* (L.) Druce, collected from the Beibu Bay of Guangxi Province, China. The fungus was identified according to its morphological characteristics and 18S rRNA sequences (Figure S1 from Supplementary Materials; GenBank No. MZ573243). The fungus was deposited at the School of Pharmacy, Qingdao University, China, and was maintained at −80 °C. For large-scale fermentation, the fresh mycelia of *A. niger* S-48 were cultured on potato dextrose agar (PDA) media at 28 °C for 4 days. The agar plugs were cut into small pieces under aseptic conditions and 100 pieces were used to inoculate 100 flasks (1L) of PDA media, each containing potato extract powder 0.9 g, glucose 3.0 g, sea salt 4.5 g, agar 3.0 g, and distilled water 0.15 L at pH 5.6. The cultures were grown under static conditions at 28 °C for 40 days.

### 2.3. Extraction and Purification

The fermented cultures were extracted by ethyl acetate (EtOAc) three times. The merged organic phase was dried *in vacuo* to yield the crude extract (5.6 g). The crude extract was subjected to column chromatography on silica gel with a gradient of MeOH/CH_2_Cl_2_ system (0/100, 1/99, 2/98, 3/97, 4/96, 5/95, 1/10, 1/9, 1/8 and 1/6, *v*/*v*) to obtain six fractions (Fr.A−Fr.F) based on TLC technology. Fr.C (1.5 g) was fractionated through a CombiFlash Rf 200 purification system, eluting with MeOH-H_2_O (50% MeOH for 40 min, 70% MeOH for 40 min, 100% MeOH for 40 min) to obtain four subfractions (Fr.C1−Fr.C4). Fr.C2 (170.0 mg) was separated by semi-preparative HPLC system (MeOH/H_2_O, 50/50, 2 mL/min) using a ZORBAX SB-C18 column to give compounds **9** (9.5 mg, *t*_R_ 12.5 min), **10** (2.5 mg, *t*_R_ 14.7 min), **11** (3.3 mg, *t*_R_ 22.2 min), and **12** (14.0 mg, *t*_R_ 32.8 min). Fr.C3 (500.0 mg) was purified by a semi-preparative HPLC system (MeOH/H_2_O, 90/10, 2 mL/min) using a C18 YMC-Pack ODS-A column to afford compounds **6** (27.0 mg, *t*_R_ 17.0 min) and **7** (20.0 mg, *t*_R_ 18.4 min). Fr.C4 (350.0 mg) was loaded onto Sephadex LH-20 column eluting with MeOH/CH_2_Cl_2_ (2/1) to obtain four fractions (Fr.C41−Fr.C44). Following a similar measure, the Fr.C42 (300.0 mg) was further purified by a semi-preparative HPLC system (CH_3_CN/H_2_O, 65/35, 2 mL/min) using a *π* NAP COSMOSIL Packed Column to obtain compounds **1** (7.2 mg, *t*_R_ 26.2 min), **2** (21.5 mg, *t*_R_ 29.8 min), **3** (5.4 mg, *t*_R_ 31.6 min), **4** (3.5 mg, *t*_R_ 37.9 min), **5** (2.6 mg, *t*_R_ 24.5 min), and **8** (8.0 mg, *t*_R_ 18.3 min).

### 2.4. Structural Modification

Hydrazine monohydrate (3.75 μL, 0.12 mmol) was joined in a solution of compound **1** (7 mg, 0.012 mmol) in absolute ethanol (5 mL), and then the mixture was stirred for eight hours under 90 °C reflux, with TLC analysis indicating the consumption of the starting material. After reaction, water (5 mL) was added to terminate the reaction. The solution was evaporated slowly in vacuo and was further extracted with ethyl acetate (3 × 5 mL). The organic phase was washed with brine and dried with sodium sulfate, filtered, and concentrated slowly using rotary evaporation. The mixture was purified by a semi-preparative HPLC system (CH_3_CN/H_2_O, 80/20, 2 mL/min) using a C18 YMC-Pack ODS-A column to obtain semisynthetic compound **13** (2.1 mg, 30.0% yield, ≥98%). A similar reaction approach was applied to compound **2** to obtain the derivative **14** (Figure S2 from Supplementary Materials).

Compound **13**: brown powder; ^1^H NMR (DMSO-*d*_6_, 600 MHz), and ^13^C NMR (DMSO-*d*_6_, 150 MHz), see Table S1 from Supplementary Materials; positive HRESIMS *m/z* [M + H]^+^ 585.1865 (calcd for C_32_H_29_N_2_O_9_, 585.1868) see Figure S11 from Supplementary Materials.

Compound **14**: brown powder; ^1^H NMR (DMSO-*d*_6_, 600 MHz) and ^13^C NMR (DMSO-*d*_6_, 150 MHz) see Table S2 from Supplementary Materials; positive HRESIMS *m/z* [M + H]^+^ 585.1870 (calcd for C_32_H_29_N_2_O_9_, 585.1868) see Figure S16 from Supplementary Materials.

### 2.5. Molecular Docking Methods

The molecular docking was carried out according to the previously described approach [11]. In general, it was carried out by SYBYL-X 2.0. The ligand molecule was drawn using the standard parameters of SYBYL-X. Their geometric conformations were energy-minimized further by employing the Tripos force field for 1000 steps, and Gasteiger–Hückel charges were calculated. The protein receptor was prepared using the standard method. PyMOL was used as a viewer for interaction between ligands and the protein receptor.

### 2.6. Inhibition of Cholesterol Absorption

Cholesterol uptake in Caco-2 cells was performed according to a previously reported method [12,13]. Caco-2 cells were received from the American Type Culture Collection (Rockvill, MD) (ATCC^®^-HTB-37^TM^). Samples in DMSO were dissolved in cell-culture medium and diluted to a concentration of 100 μM. Ezetimibe (100 μM) was used as a positive control for this study. The fluorescence was measured at the excitation wavelength of 485 nm and emission wavelength of 535 nm. BCA kit (Thermo Fisher Scientific, Waltham, MA, USA) was used to determine protein concentration with bovine serum albumin as standard. The whole protein represented the total number of cells used for normalization. The effects were expressed as the percentage of cholesterol uptake corresponding to the control values.

### 2.7. Antimicrobial Activities

Bacterial and fungal pathogens (*Staphylococcus aureus*, *Pseudomonas aeruginosa*, *Bacillus subtilis*, *Escherichia coli*, *Candida albicans*, *Fusarium foetens*, *F. solanum*, *F. mangiferae*, *F. oxysporum* f. sp. cubense, *F. graminearum*, *Colletotrichum musae* (ACCC 31244), *C. coccodes* (ACCC 36067), *C. asianum*, *Cucumber fusarium wilt*, *Cowpea wilt*, *Nectria* sp., and *Alternaria solani*) were used to assess the antimicrobial activities of compounds. Antimicrobial activity was carried out by the paper-agar disk diffusion assay as in the previously reported method [14]. Samples were dissolved in MeOH and diluted to a concentration of 4 mg/mL. Next, 10 μL of the sample solutions was dropped into 6 mm sterile filter paper disks, and the filter paper disks were placed evenly on the solid media with test strains [14]. Streptomycin, actidione, and fluconazole were used as the positive controls, and MeOH was used as the blank control. Antimicrobial activity was evaluated by the diameter of inhibitory zones in the solid media.

## 3. Results and Discussion

### 3.1. Identification of Metabolites

The structures of compounds **1**–**12** (Figure 1), including eight known N*γ*Ps, fonsecinone A (**1**) [15], aurasperone A (**2**) [15], asperpyrones B and C (**3** and **4**) [16], rubasperone B (**5**) [17], aurasperone E (**6**) [18], fonsecinone C (**7**) [18], and flavasperone (**8**) [19], as well as four known coumarins, orlandin (**9**) [16], 4,7-dimethoxy-5-methylcoumarin (**10**) [20], 7-hydroxy-4-methoxy-5-methylcoumarin (**11**) [21], and desmethylkotanin (**12**) [22], were identified by comparing their NMR data and MS spectra with previously reported data. The ECD spectra of compounds **1**–**4** (see Figures S53 and S54) indicated that the absolute configurations of compounds **1**–**4** were (*R*), which was consistent with the previously reported data [18]. Specifically, compounds **1**–**8** are N*γ*Ps compounds, which were previously isolated from *Aspergillus* spp. The structures of N*γ*Ps consist of both naphthalene and *γ*-pyrone moiety, and there are monomeric and dimeric forms [23]. Moreover, compounds **1** and **2** displayed antibacterial activities against *Helicobacter pylori* [16]. The biological activity of N*γ*Ps is inextricably linked to its structure. The radical scavenging potential of N*γ*Ps is related to the number of hydroxyl substitutes on the skeleton [8], and the phytotoxicity of N*γ*Ps is connected to the *γ*-pyrone ring [24]. Compounds **9**–**12** are coumarin derivatives, and they had shown antibacterial and antifungal activities [7].

### 3.2. Modification and Identification of Semisynthetic NγPs

In order to evaluate the importance of the pyran ring in N*γ*Ps on bioactivity, compounds **1** and **2** were tentatively modified based on a benzohydrazide reaction [25]. Nucleophilic attack of hydrazine at C-2 of the N*γ*P, followed by ring opening, further nucleophilic attack of the second nitrogen atom at the carbonyl carbon, and subsequent dehydration led to the formation of the pyrazole ring (Figure S3 from Supplementary Materials). Finally, compounds **13** and **14** were obtained as the corresponding semisynthetic products consisting of the partial N*γ*P and the pyrazole ring (Figure 1).

Compound **13** was obtained as a brown powder. Its molecular formula was determined to be C_32_H_28_N_2_O_9_ on the basis of HRESIMS analysis, indicating twenty degrees of unsaturation. Compared to compound **1**, the molecular weight of compound **13** went up by 14 amu. Therefore, only one pyrone site of bis-N*γ*Ps was subjected to reaction. Specifically, the unreacted pyrone was confirmed by detailed analysis of ^1^H and ^13^C NMR data, such as C-9 (*δ*_C_ 119.7), together with the key HMBC correlation signals (Figure S4 from Supplementary Materials) from H-7 (*δ*_H_ 7.24) to C-9/C-10a (*δ*_C_ 107.4), from H-6 (*δ*_H_ 7.10) to C-4a (*δ*_C_ 108.3)/C-7 (*δ*_C_ 101.7), and from H-3 (*δ*_H_ 6.52) to C-2 (*δ*_C_ 168.1)/C-4a. The key HMBC correlation signals (Figure S4 from Supplementary Materials) of 3H-14′ (*δ*_H_ 2.35) with C-4′ (*δ*_C_ 105.9)/C-5′ (*δ*_C_ 138.7), and of H-4′ (*δ*_H_ 6.93) with C-3′ (*δ*_C_ 148.5)/C-5′, indicated compound **13** was a pyrazole-type compound. Thus, the structure of semisynthetic compound **13** was determined.

Compound **14** was obtained as a brown powder and had the same molecular formula C_32_H_28_N_2_O_9_ as **13** from the HRESIMS analysis. It showed that the semisynthetic compound **14** contained a pyrazole ring. This was validated by key HMBC correlation (Figure S5 from Supplementary Materials) signals from 3H-14′ (*δ*_H_ 2.34) to C-4′ (*δ*_C_ 105.9)/C-5′ (*δ*_C_ 138.7), and from H-4′ (*δ*_H_ 6.92) to C-3′ (*δ*_C_ 148.6)/C-5′. Similarly, the unreacted pyrone was detected through detailed analysis of ^1^H and ^13^C NMR data, such as C-7 (*δ*_C_ 120.1), together with the key HMBC correlation signals (Figure S5 from Supplementary Materials) of H-9 (*δ*_H_ 7.27) with C-5a (*δ*_C_ 110.7)/C-7, of H-10 (*δ*_H_ 7.37) with C-5a/C-9 (*δ*_C_ 101.8), and of H-10 with C-4 (*δ*_C_ 184.1). Therefore, the structure of compound **14** was determined.

### 3.3. Biological Activities

**Figure 2:**
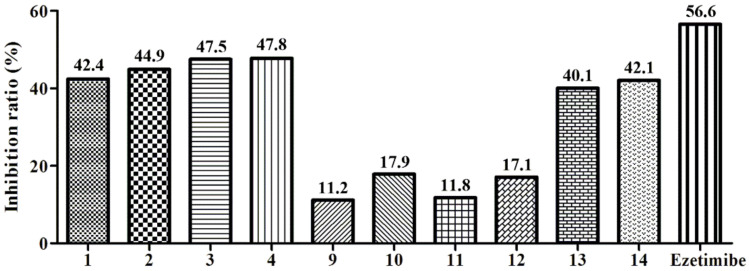
Compounds **1**–**4** and **9**–**14** at the concentration of 100 μM, the rate of inhibition of cholesterol esterase. Ezetimibe was used as a positive control.

**Figure 3:**
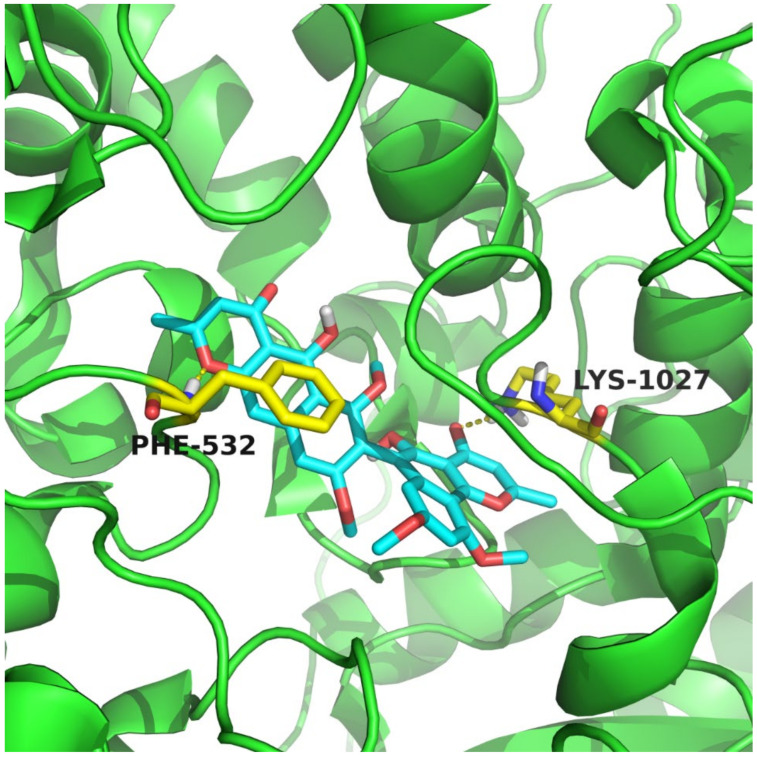
The binding mode of compound **4** with NPC1L1. Compound **4** was shown in cyan. The figure was produced with PyMOL. The related amino acids were shown in yellow.

Niemann-Pick C1-Like 1 (NPC1L1) is a key target involving cholesterol cellar uptake [13]. Both compounds **1**–**4** and **9**–1**4** were tested for cholesterol absorption inhibition activity [26]. Compounds **1**–**4** showed similar inhibitory activity in 100 µM compared with ezetimibe, the only FDA-approved NPC1L1 inhibitor (Figure 2) [12]. The results showed that compound **4** had the highest inhibitory activity for reducing cholesterol absorption among all compounds, and deserves further evaluation. The reason for the function of N*γ*Ps may be related to the pyrones site. To elucidate the binding mechanism of compound **4** and NPC1L1, molecular docking was performed to predict the binding mode of compound **4**. Interestingly, Lys1027 and Phe532 were involved in the formation of hydrogen bonds with N*γ*Ps (Figure 3). In addition, compound **4** and Phe532 interacted by π-π stacking, which further increased the affinity of compound **4** with NPC1L1. Through decreasing cholesterol absorption with a NPC1L1 inhibitor, ezetimibe was the first and only inhibitor approved for the treatment of hypercholesterolemia for nearly 20 years [12]. Natural products from marine fungi could be assumed to obtain NPC1L1 potential inhibitors.

The antimicrobial activities of all fourteen compounds, including eight N*γ*Ps, four coumarins, and two semisynthetic compounds, were evaluated by the paper-agar disk-diffusion methods. However, none of them showed activities against test pathogens, including Gram-positive and Gram-negative bacteria.

## 4. Conclusions

In summary, this study describes the isolation, identification, cholesterol absorption inhibition activity, and antimicrobial activity of N*γ*Ps and coumarins from the marine-derived fungus *A. niger* S-48. Their structures were identified by 1D and 2D NMR spectroscopic data and MS analysis. In addition, the reaction of natural N*γ*Ps with hydrazine afforded two semisynthetic compounds, containing naphtho-gamma-pyrones and the pyrazole ring. It has to be noted that benzohydrazide reaction was rarely reported on N*γ*Ps. All isolated or semisynthetic compounds had no effects during the assays of antimicrobial activities. Notably, compound **4**, as one of the isolated N*γ*Ps, showed activity for reducing cholesterol absorption comparable to the positive drug ezetimibe. As far as we know, this is the first report to curb cholesterol cellar uptake activity using N*γ*Ps. Therefore, we obtain N*γ*Ps as potential NPC1L1 inhibitors, which call for further research.

## Data Availability

Not applicable.

## Supplementary Materials

The following supporting information can be downloaded at: https://www.mdpi.com/article/10.3390/molecules27082514/s1, Figures S1–S57; Tables S1–S5. NMR data, NMR spectra, HRESIMS of compounds **13** and **14**. NMR data, NMR spectra, HRESIMS or ESIMS of compounds **1**–**12**. Experimental ECD spectra and optical rotations of compounds **1**–**4**.

## Sample Availability

Samples of the compounds are available from the authors.
