# Supplementary material for: Naphtho-Gamma-Pyrones (NγPs) with Obvious Cholesterol Absorption Inhibitory Activity from the Marine-Derived Fungus Aspergillus niger S-48
Source: Molecules. 2022 Apr 13;27(8):2514. doi: 10.3390/molecules27082514 (PMC9029069; doi:10.3390/molecules27082514)
Supplement: Supplementary file 1 [file molecules-27-02514-s001.zip › molecules-1668245-supplementary.pdf]

molecules

Supplementary Materials

Naphtho-Gamma-Pyrones (NγPs) with Obvious Cholesterol Absorption Inhibitory Activity from the Marine-Derived Fungus *Aspergillus niger* S-48

Chang-Zheng Wu [1,†], Xiao-Ping Peng [1,†], Gang Li [1], Qi Wang [1] and Hong-Xiang Lou [1,2,*]

[1] Department of Natural Medicinal Chemistry and Pharmacognosy, School of Pharmacy, Qingdao University, Qingdao 266021, China; 2019026576@qdu.edu.cn (C.-Z.W.); pengxiaoping@qdu.edu.cn (X.-P.P.); gang.li@qdu.edu.cn (G.L.); wangqi@hmfl.ac.cn (Q.W.)

[2] Key Laboratory of Chemical Biology of Ministry of Education, Department of Natural Product Chemistry, School of Pharmaceutical Sciences, Shandong University, Jinan, China

* Correspondence: louhongxiang@sdu.edu.cn ; Tel.: +86-531-8838-2012

† These authors contributed equally to this work.

Contents:

Figures S1–S57

Table S1–S5

Figure S1 The 18S rRNA gene sequences data of *Aspergillus niger* S-48……………5

Figure S2 Reaction schemes for semisynthetic compounds **13** and **14**…………......…6

Figure S3 Possible reaction pathway for compounds **1** and **2**………………………..7

Figure S4 Key HMBC correlations of compound **13**…………………………………8

Figure S5 Key HMBC correlations of compound **14**…………………………………9

Figure S6 ^{1}H NMR spectrum (600 MHz) of compound **13** in DMSO-d_6…...............10

Figure S7 ^{13}C NMR spectrum (150 MHz) of compound **13** in DMSO-d_6……..........11

Figure S8 ^{1}H-^{1}H COSY spectrum (600 MHz) of compound **13** in DMSO-d_6….........12

Figure S9 HMBC spectrum (600 MHz) of compound **13** in DMSO-d_6…..................13

Figure S10 HSQC spectrum (600 MHz) of compound **13** in DMSO-d_6……………....14

Figure S11 HRESIMS spectrum of compound **13**…..........………….....………......…..15

Figure S12 ^{1}H NMR spectrum (600 MHz) of compound **14** in DMSO-d_6…………16

Figure S13 ^{13}C NMR spectrum (150 MHz) of compound **14** in DMSO-d_6………....17

Figure S14 HMBC spectrum (600 MHz) of compound **14** in DMSO-d_6……………...18

Figure S15 HSQC spectrum (600 MHz) of compound **14** in DMSO-d_6……….....…19

Figure S16 HRESIMS spectrum of compound **14**……………………………….........20

Figure S17 ^{1}H NMR spectrum (500 MHz) of compound **1** in CDCl$_3$……………….21

Figure S18 ^{13}C NMR spectrum (125 MHz) of compound **1** in CDCl$_3$………...……22

Figure S19 HRESIMS spectrum of compound **1**..……………….........................23

Figure S20 ^{1}H NMR spectrum (500 MHz) of compound **2** in CDCl$_3$………………24

Figure S21 ^{13}C NMR spectrum (125 MHz) of compound **2** in CDCl$_3$……………...25

Figure S22 HRESIMS spectrum of compound **2**………………………………..........26

Figure S23 ^{1}H NMR spectrum (500 MHz) of compound **3** in CDCl$_3$………………27

Figure S24 ^{13}C NMR spectrum (125 MHz) of compound **3** in CDCl$_3$……………...28

Figure S25 HRESIMS spectrum of compound **3**………………………………..........29

Figure S26 ^{1}H NMR spectrum (500 MHz) of compound **4** in CDCl$_3$………………30

Figure S27 ^{13}C NMR spectrum (125 MHz) of compound **4** in CDCl$_3$………...…31

Figure S28 HRESIMS spectrum of compound **4**……………………………...........32

Figure S29 ^{1}H NMR spectrum (600 MHz) of compound **5** in CDCl$_3$……………….33

Figure S30 ^{13}C NMR spectrum (150 MHz) of compound **5** in CDCl$_3$………...……34

Figure S31 ESIMS spectrum of compound **5**……………………………….............35

Figure S32 ^{1}H NMR spectrum (500 MHz) of compound **6** in DMSO-d_6…………...36

Figure S33 ^{13}C NMR spectrum (125 MHz) of compound **6** in DMSO-d_6……….…37

Figure S34 ESIMS spectrum of compound **6**……………………………….................38

Figure S35 ^{1}H NMR spectrum (500 MHz) of compound **7** in DMSO-d_6……….….39
Figure S36 ^{13}C NMR spectrum (125 MHz) of compound **7** in DMSO-d_6………..…40
Figure S37 ESIMS spectrum of compound **7**……………………….…...............41
Figure S38 ^{1}H NMR spectrum (500 MHz) of compound **8** in CDCl$_3$………………42
Figure S39 ^{13}C NMR spectrum (125 MHz) of compound **8** in CDCl$_3$…………..…43
Figure S40 ESIMS spectrum of compound **8**……………………….…...............44
Figure S41 ^{1}H NMR spectrum (500 MHz) of compound **9** in DMSO-d_6……...……45
Figure S42 ^{13}C NMR spectrum (125 MHz) of compound **9** in DMSO-d_6…………..46
Figure S43 ESIMS spectrum of compound **9**……………………….……...........47
Figure S44 ^{1}H NMR spectrum (600 MHz) of compound **10** in DMSO-d_6………..…48
Figure S45 ^{13}C NMR spectrum (150 MHz) of compound **10** in DMSO-d_6…………49
Figure S46 ESIMS spectrum of compound **10**……………………….…..............50
Figure S47 ^{1}H NMR spectrum (500 MHz) of compound **11** in DMSO-d_6………..…51
Figure S48 ^{13}C NMR spectrum (125 MHz) of compound **11** in DMSO-d_6…………52
Figure S49 ESIMS spectrum of compound **11**……………………….….............53
Figure S50 ^{1}H NMR spectrum (500 MHz) of compound **12** in CDCl$_3$…………..…54
Figure S51 ^{13}C NMR spectrum (125 MHz) of compound **12** in CDCl$_3$……………55
Figure S52 ESIMS spectrum of compound **12**……………………….….............56
Figure S53 Experimental ECD spectra of compound **1**-**4**…………..……………...57
Figure S54 Experimental ECD spectra of compound **1**, **2**, and **4** in the previously reported data…………………………………………………………………57
Figure S55 Comparison of ^{1}H NMR spectrums (500 MHz) of compounds **1**-**4** in CDCl$_3$…………………………………………………………………………...58
Figure S56 Comparison of ^{13}C NMR spectrums (125 MHz) of compounds **1**-**4** in CDCl$_3$…………………………………………………………………………...59
Figure S57 ^{1}H and ^{13}C NMR data of compounds **5**-**12**…………………..............61
Table S1 ^{1}H NMR (600 MHz) and ^{13}C NMR (150 MHz) data of compound **13** in DMSO-d_6……………………………………………………………………..62
Table S2 ^{1}H NMR (600 MHz) and ^{13}C NMR (150 MHz) data of compound **14** in DMSO-d_6……………………………………………………………………..63
Table S3 ^{1}H NMR (500 MHz) data of compounds **1**-**4** in CDCl$_3$……………………64
Table S4 ^{13}C NMR (125 MHz) data of compounds **1**-**4** in CDCl$_3$…………………..65
Table S5 Optical rotations of compounds **1**-**4** in MeOH at 20 °C……………..….....67

TGCGGAAGGATCATTACCGAGTGCGGGTCCTTTGGGCCCAACCTCCCATCCGTGTCTATTGTAC

CCTGTTGCTTCGGCGGGCCCGCCGCTTGTCGGCCGCCGGGGGGGCGCCTCTGCCCCCCGGGCCC

GTGCCCGCCGGAGACCCCAACACGAACACTGTCTGAAAGCGTGCAGTCTGAGTTGATTGAATG

CAATCAGTTAAAACTTTCAACAATGGATCTCTTGGTTCCGGCATCGATGAAGAACGCAGCGAA

ATGCGATAACTAATGTGAATTGCAGAATTCAGTGAATCATCGAGTCTTTGAACGCACATTGCGC

CCCCTGGTATTCCGGGGGGCATGCCTGTCCGAGCGTCATTGCTGCCCTCAAGCCCGGCTTGTGT

GTTGGGTCGCCGTCCCCCTCTCCGGGGGGACGGGCCCGAAAGGCAGCGGCGGCACCGCGTCCG

ATCCTCGAGCGTATGGGGCTTTGTCACATGCTCTGTAGGATTGGCCGGCGCCTGCCGACGTTTT

CCAACCATTCTTTCCAGGTTGACCTCGGATCAGGTAGGGATACCCGCTGAACTTAAGCATATC

Figure S1. The 18S rRNA gene sequences data of *Aspergillus niger* S-48.

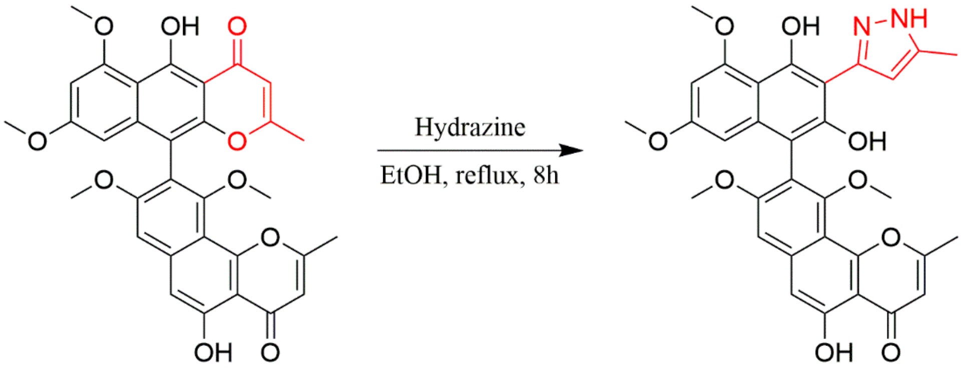

1 **13**

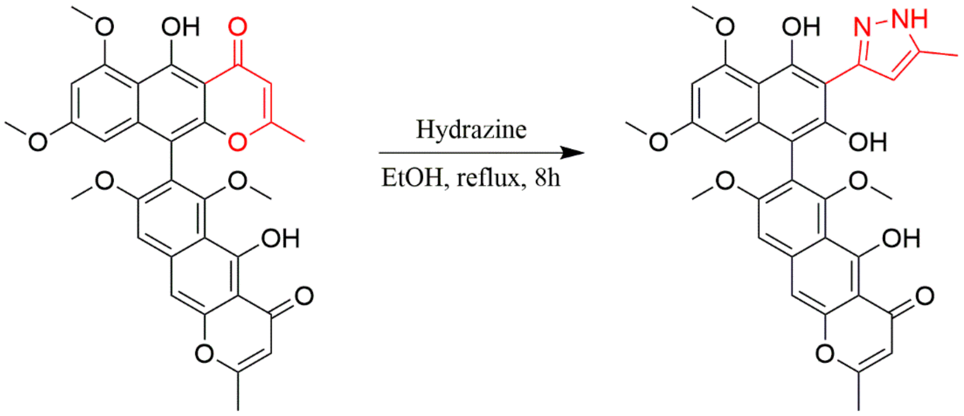

2 **14**

Figure S2. Reaction schemes for semisynthetic compounds **13** and **14**.

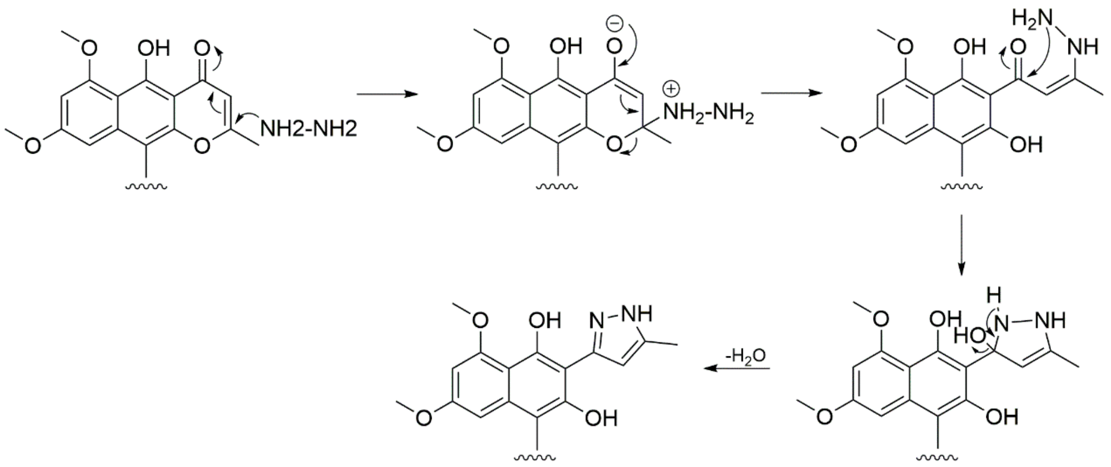

Figure S3. Possible reaction pathway for compounds **1** and **2**.

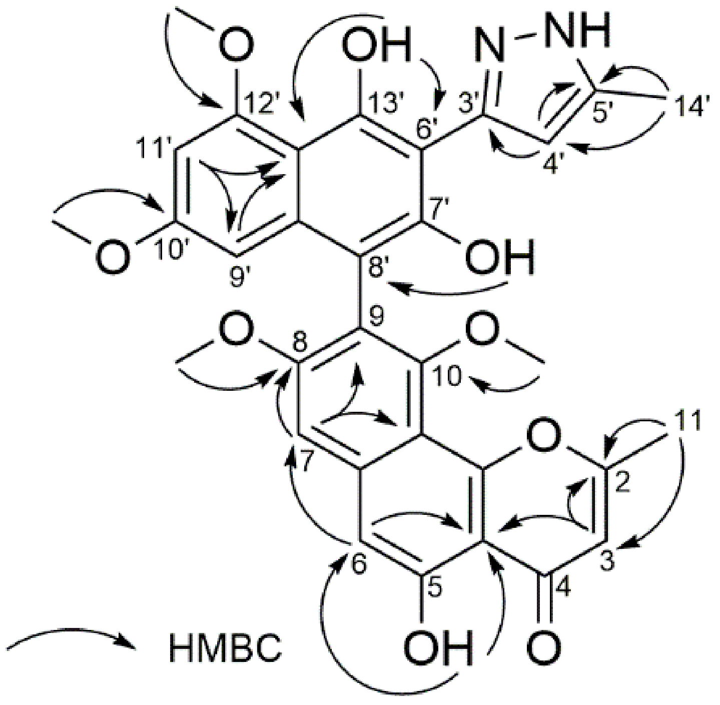

13

Figure S4. Key HMBC correlations of compound **13**.

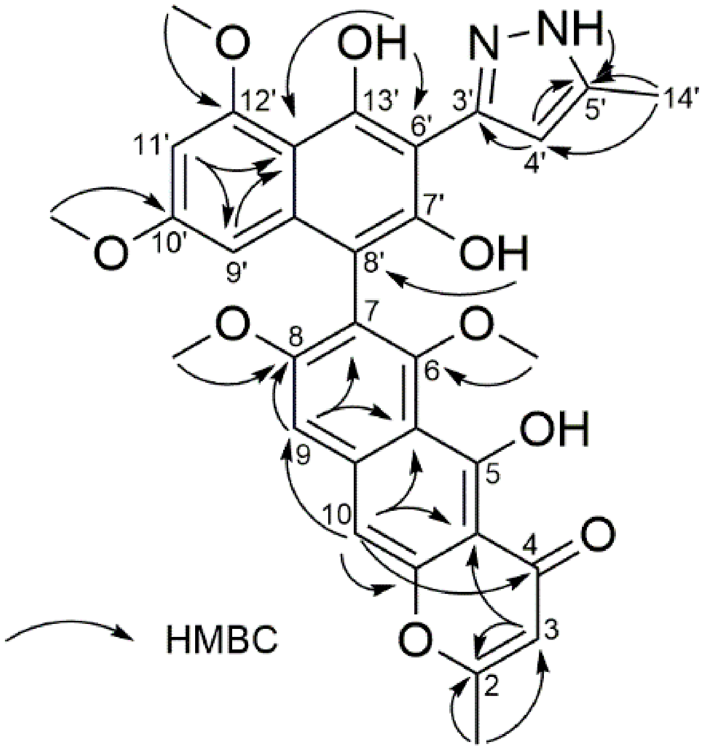

14

Figure S5. Key HMBC correlations of compound **14**.

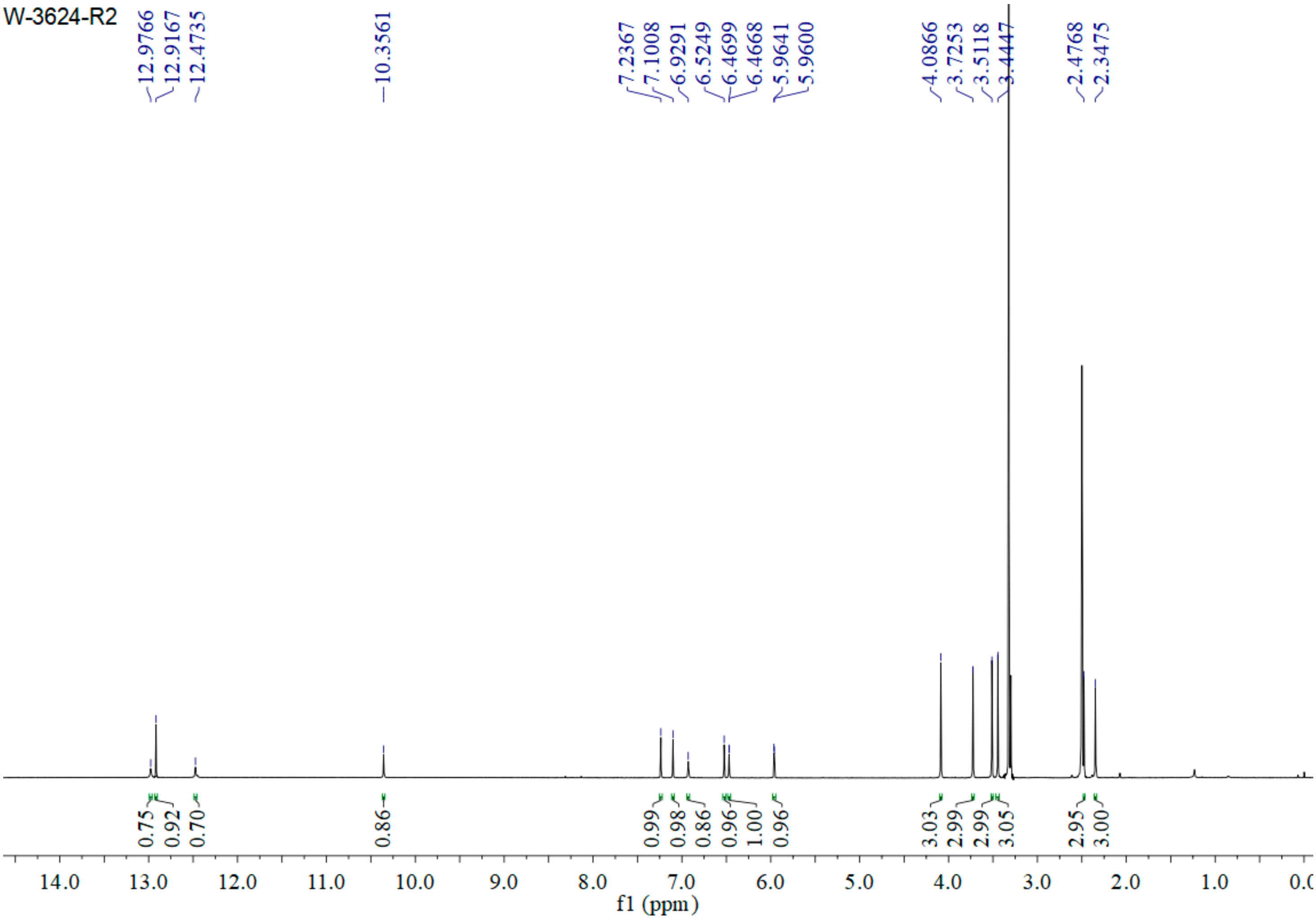

Figure S6. ^{1}H NMR spectrum (600 MHz) of compound **13** in DMSO-d_6.

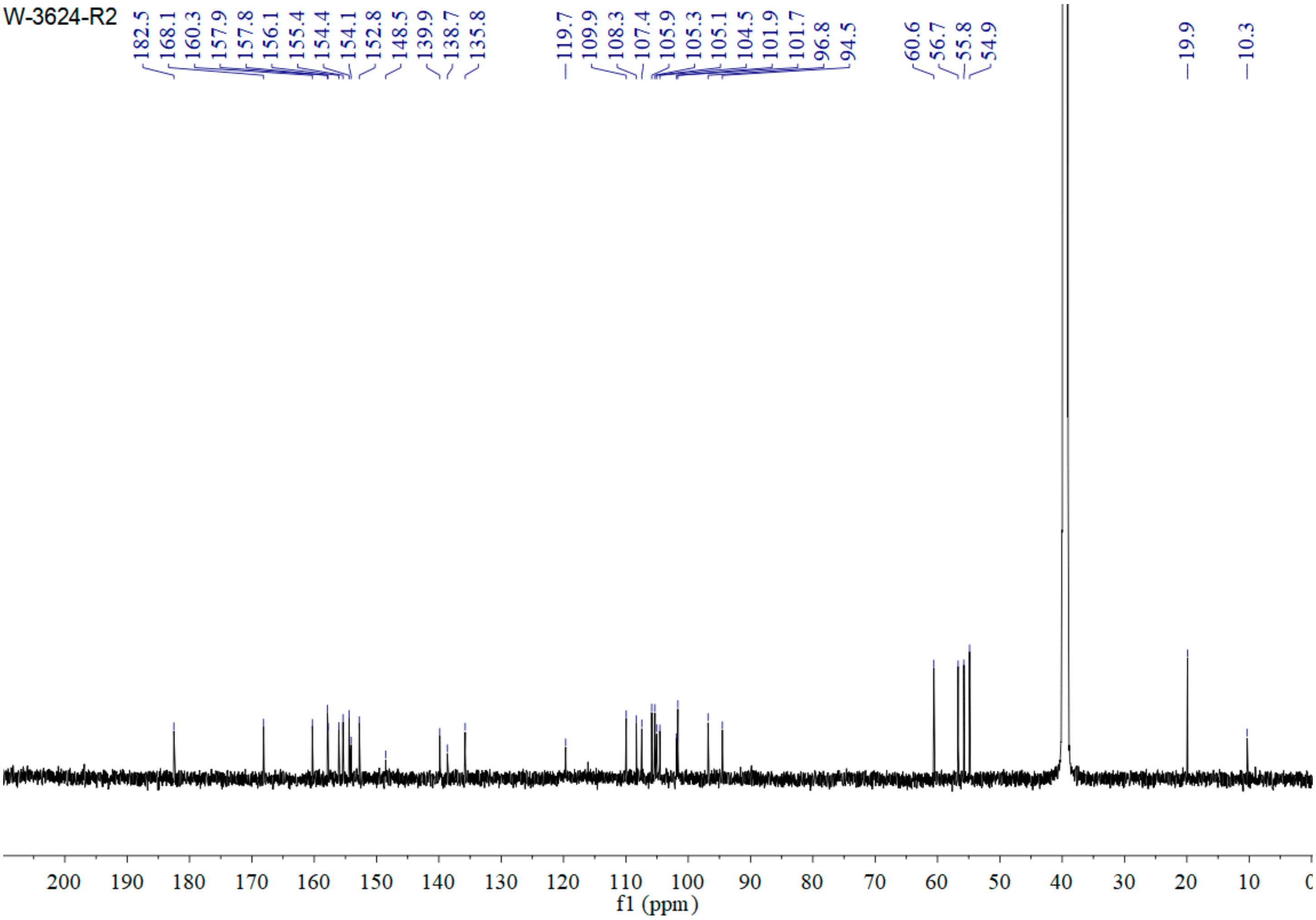

Figure S7. ^{13}C NMR spectrum (150 MHz) of compound **13** in DMSO-d_6.

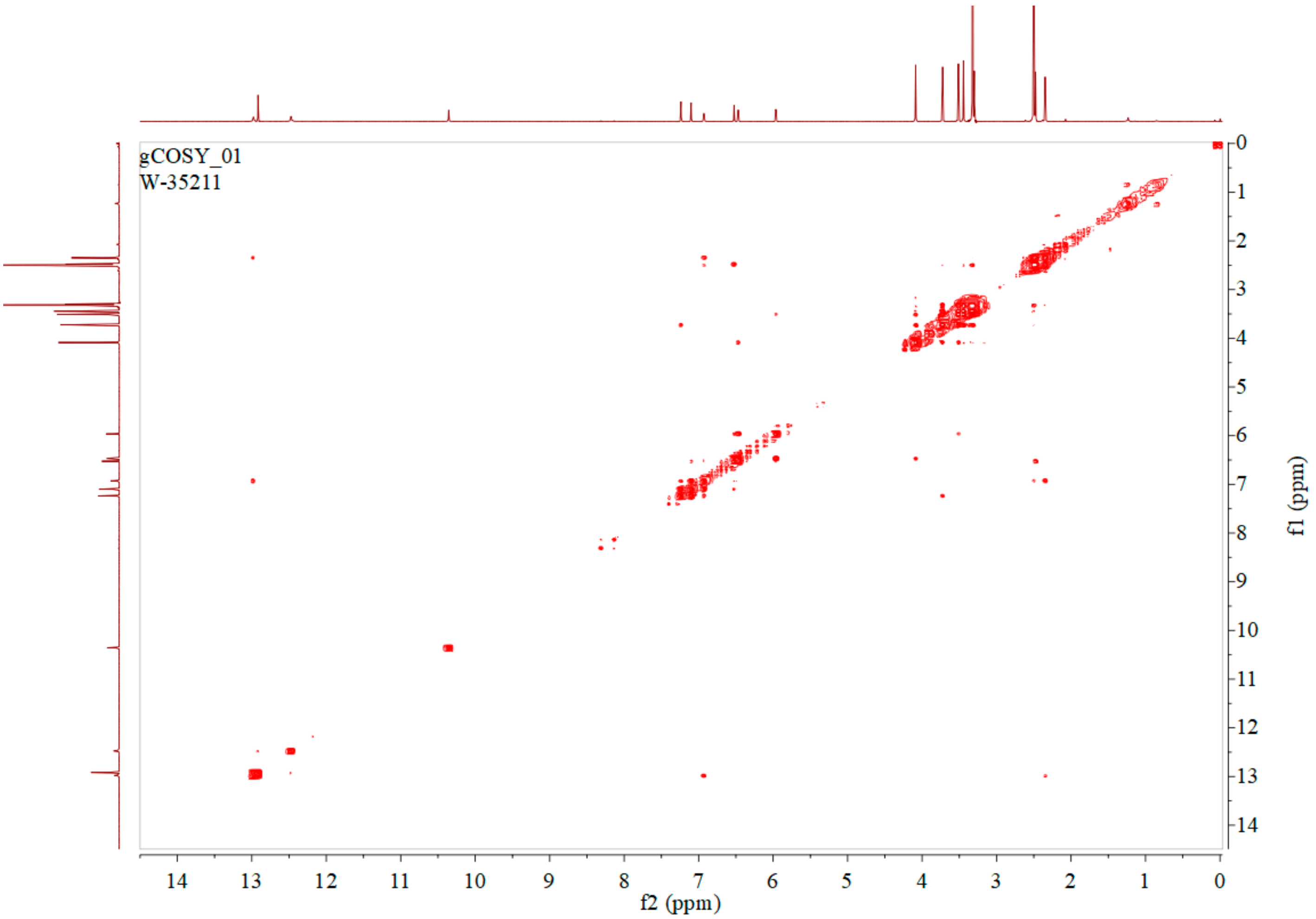

Figure S8. ^{1}H-^{1}H COSY spectrum (600 MHz) of compound **13** in DMSO-d_6.

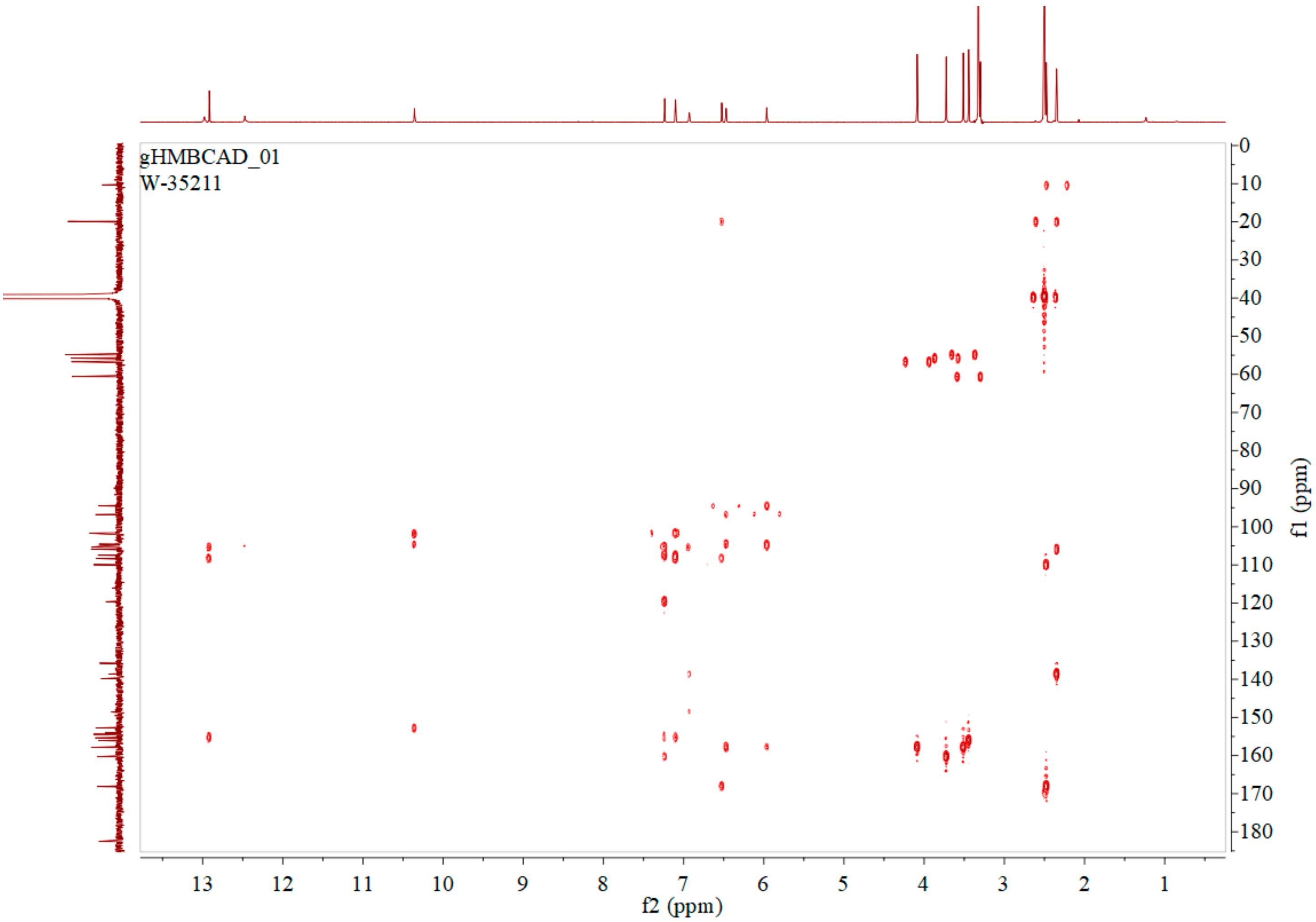

Figure S9. HMBC spectrum (600 MHz) of compound **13** in DMSO-d_6.

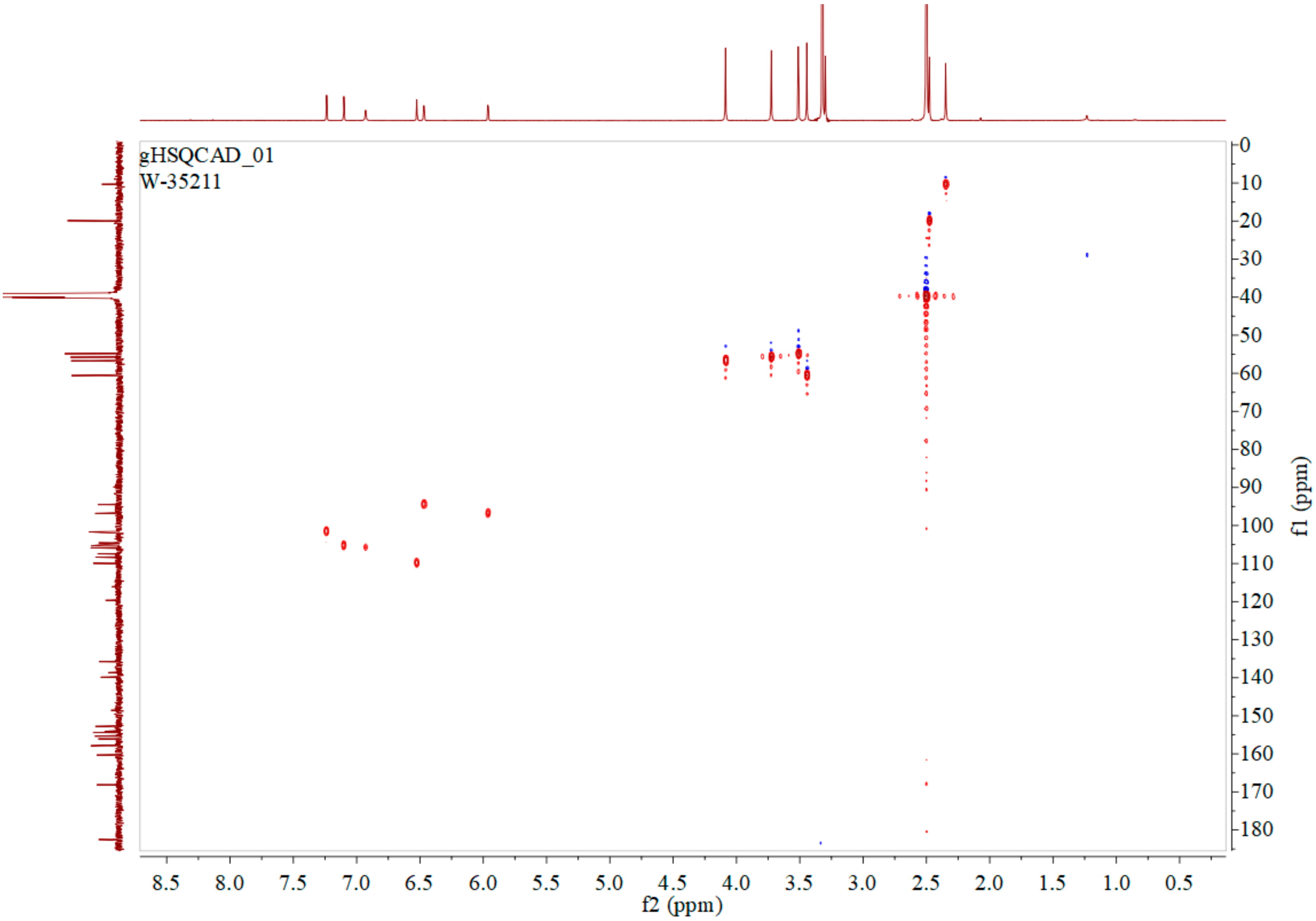

Figure S10. HSQC spectrum (600 MHz) of compound **13** in DMSO-d_6.

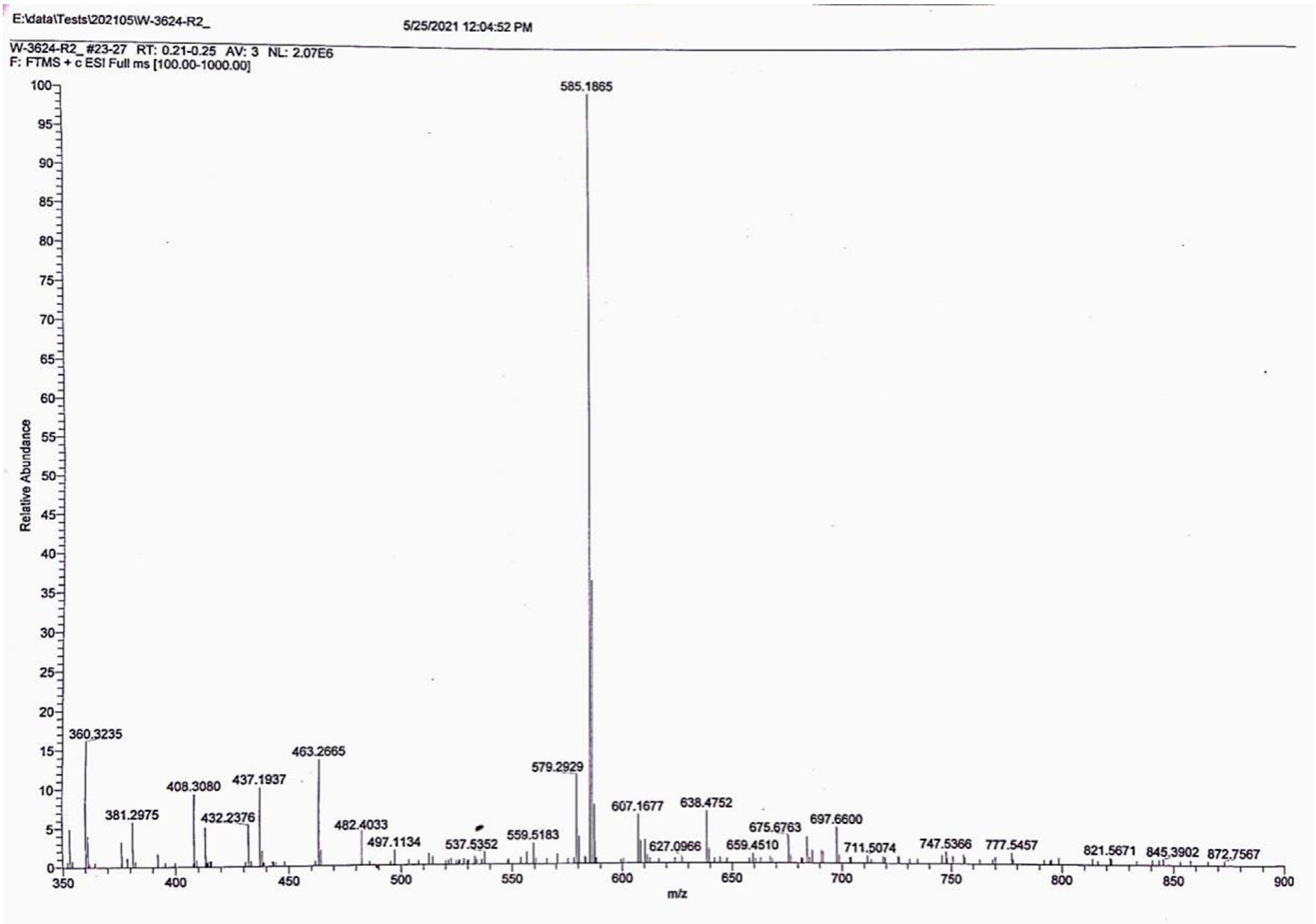

Figure S11. HRESIMS spectrum of compound **13**.

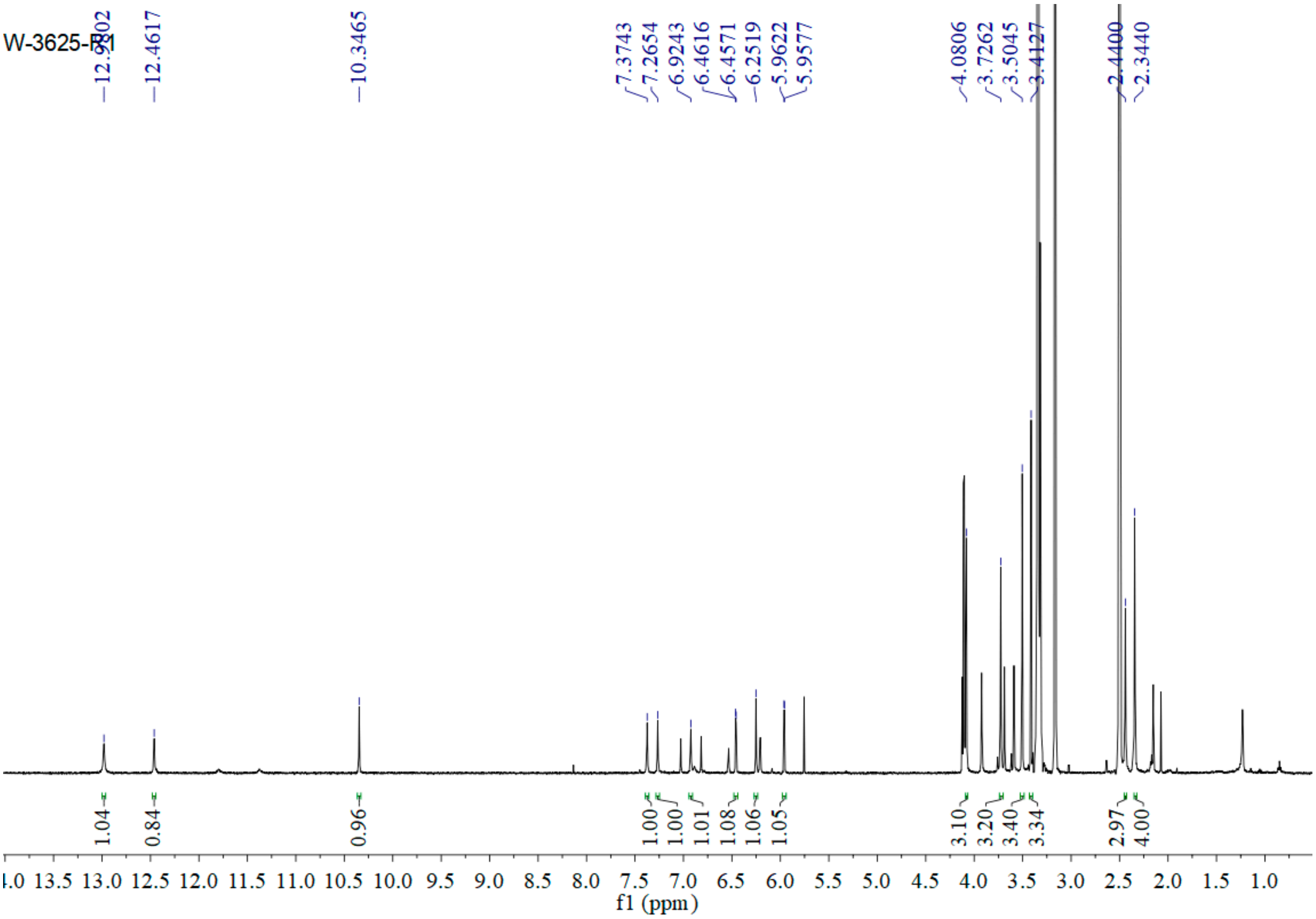

Figure S12. ^{1}H NMR spectrum (600 MHz) of compound **14** in DMSO-d_6.

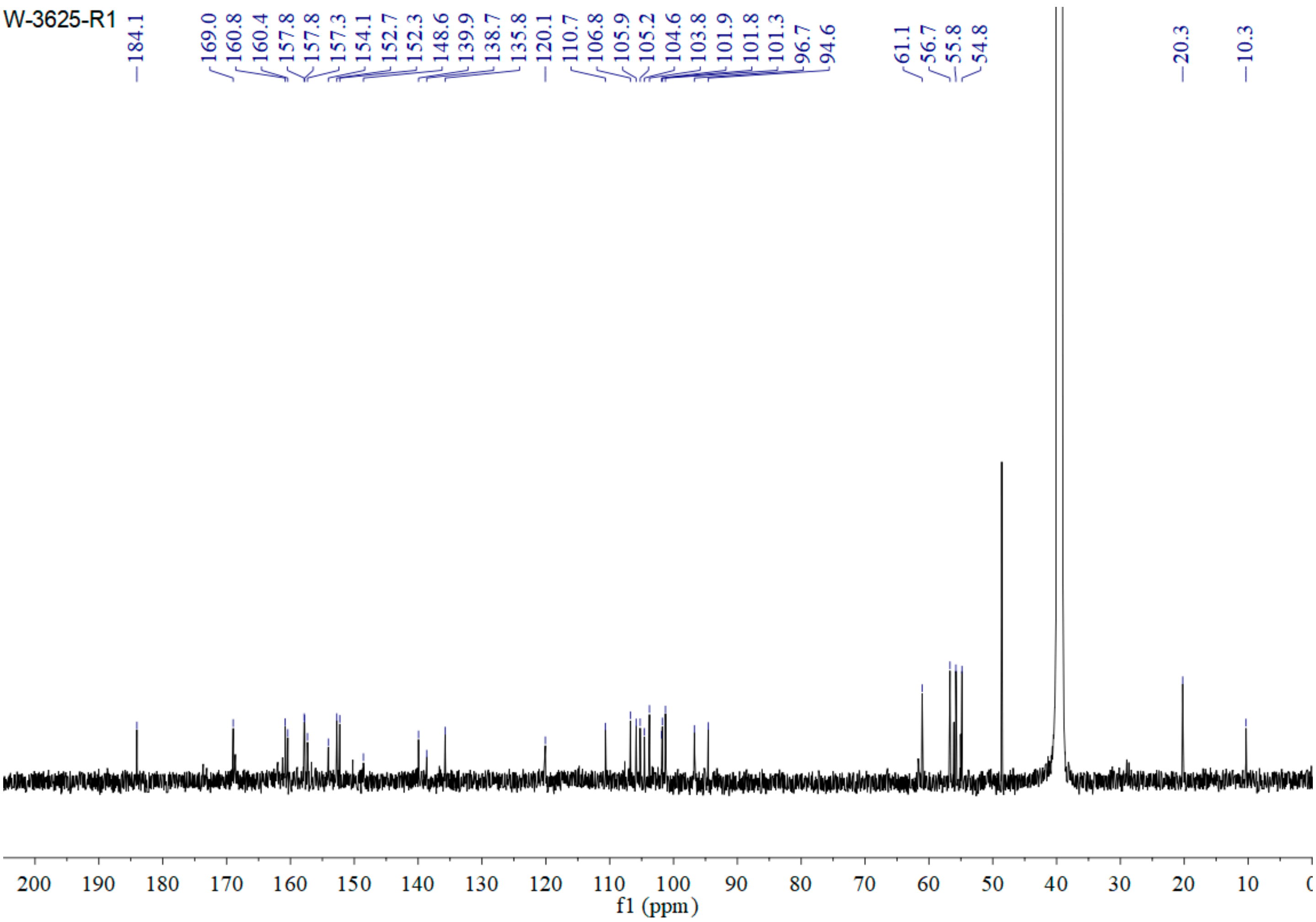

Figure S13. ^{13}C NMR spectrum (150 MHz) of compound **14** in DMSO-d_6.

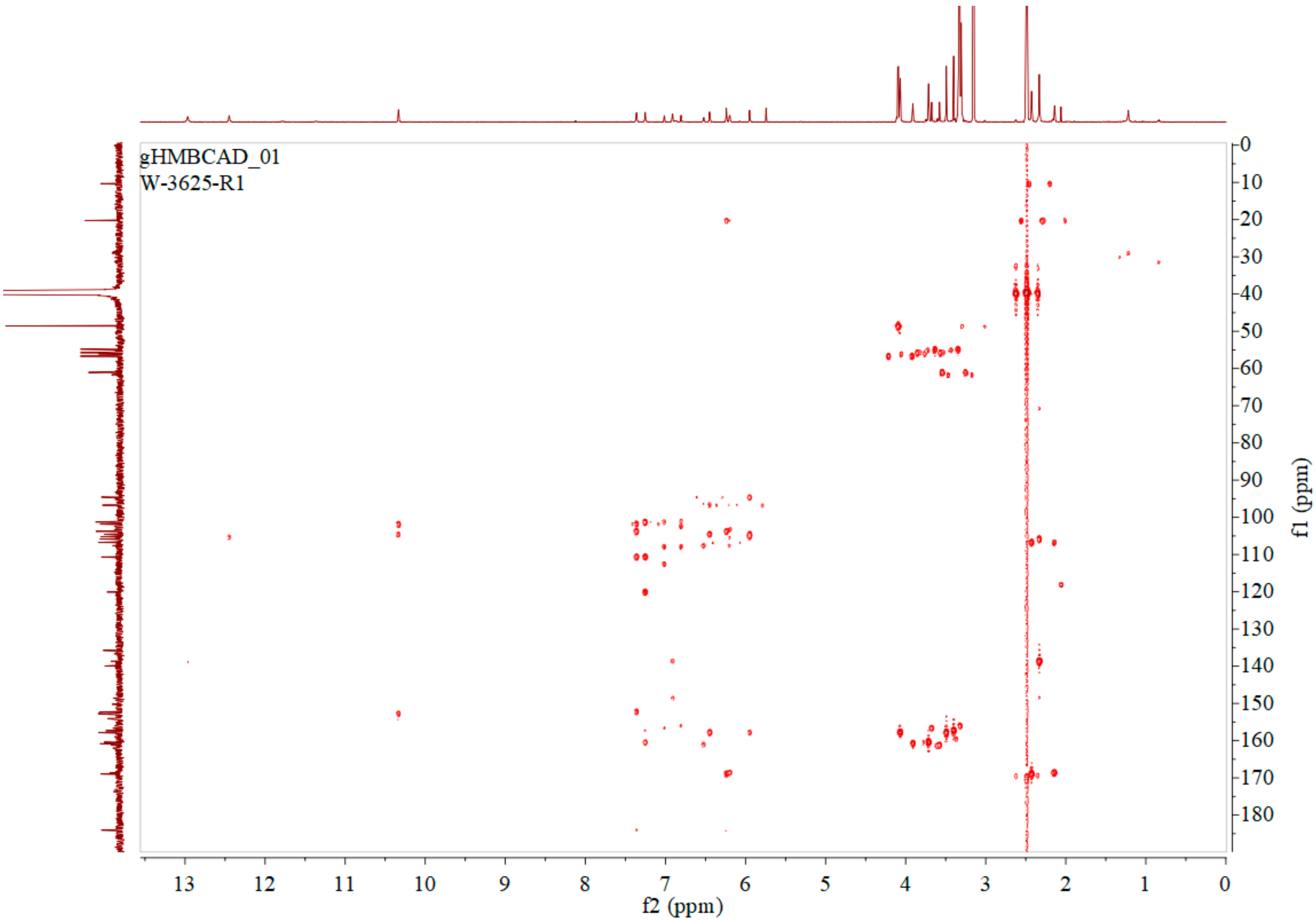

Figure S14. HMBC spectrum (600 MHz) of compound **14** in DMSO-d_6.

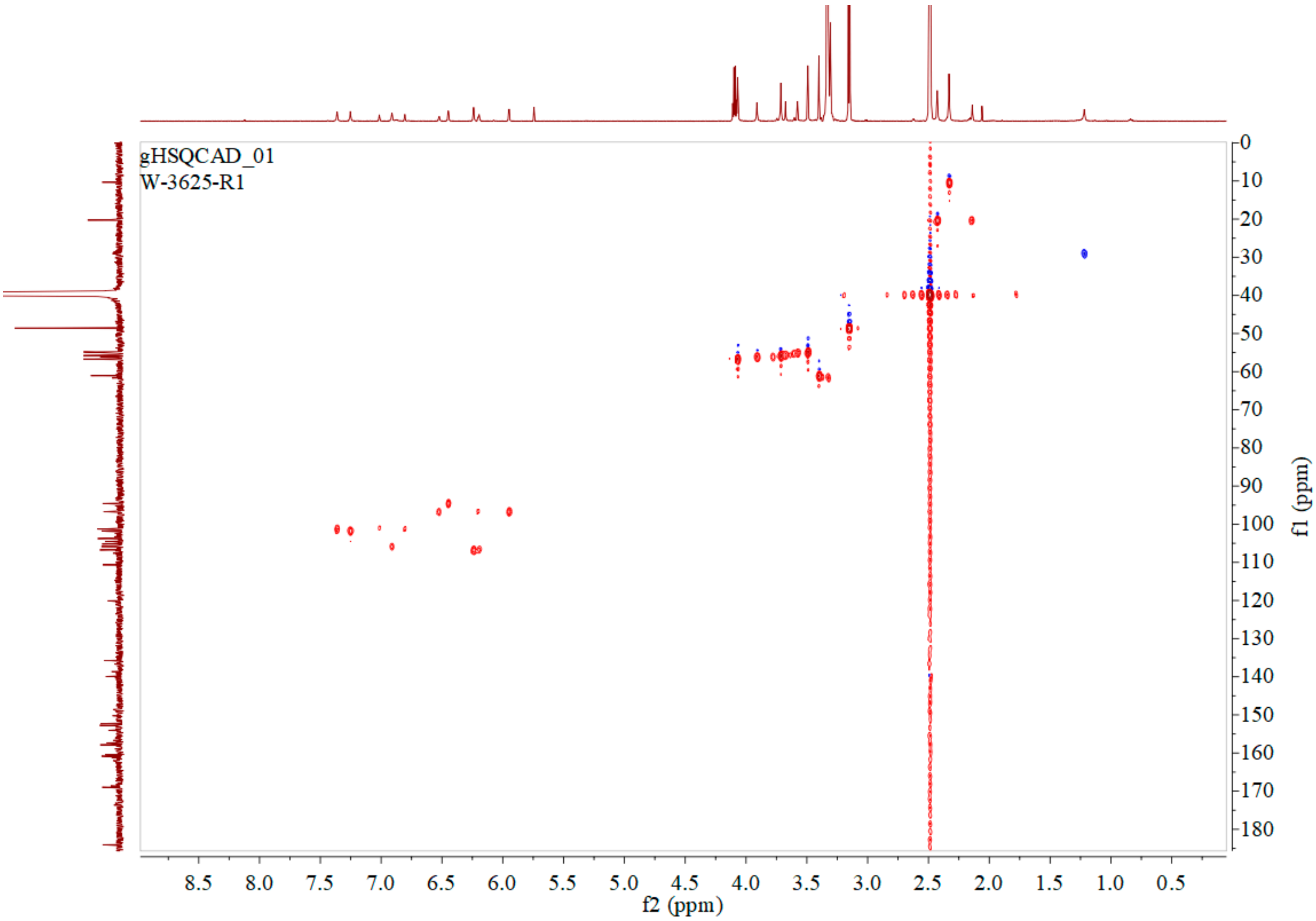

Figure S15. HSQC spectrum (600 MHz) of compound **14** in DMSO-d_6.

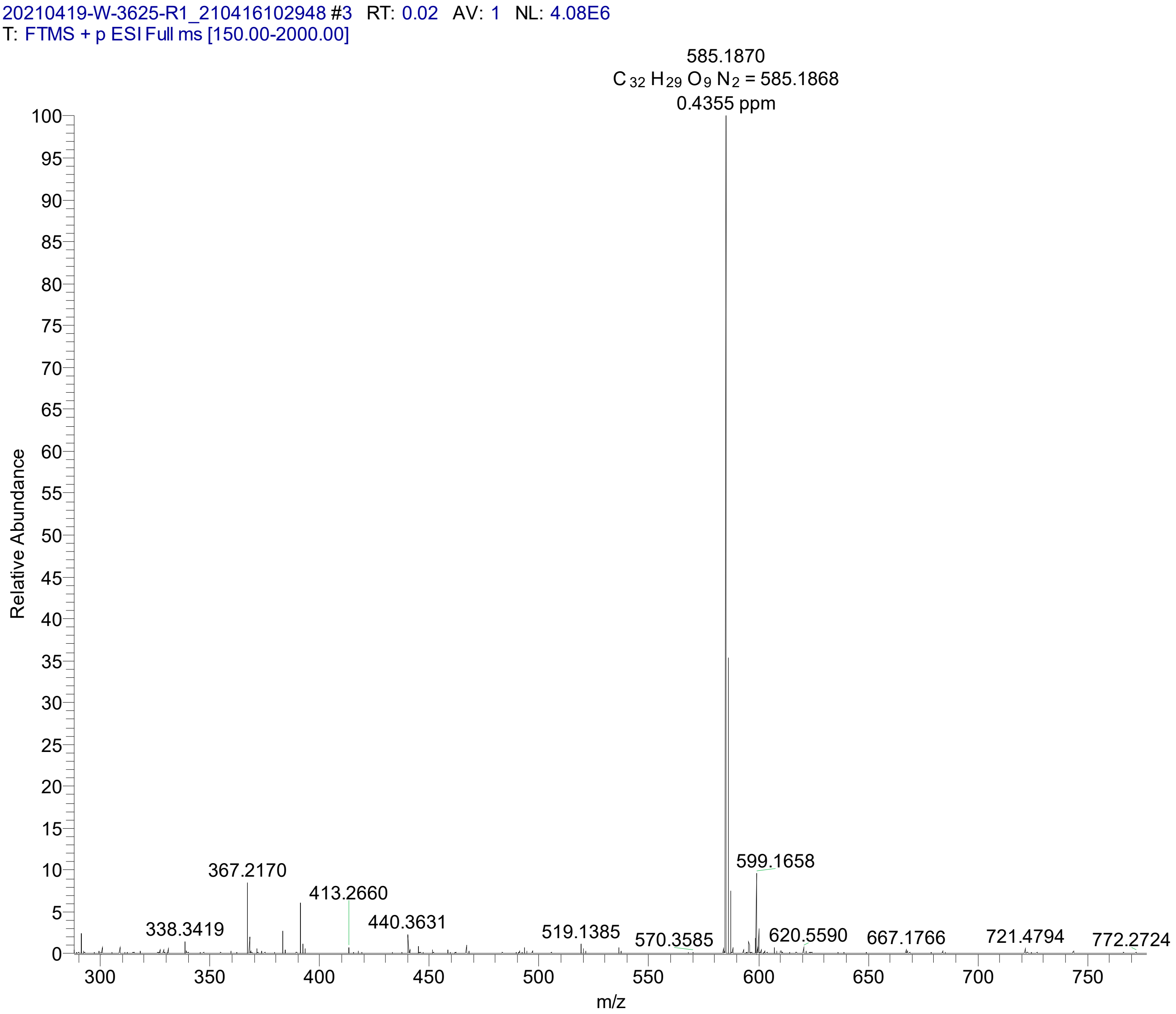

Figure S16. HRESIMS spectrum of compound **14**.

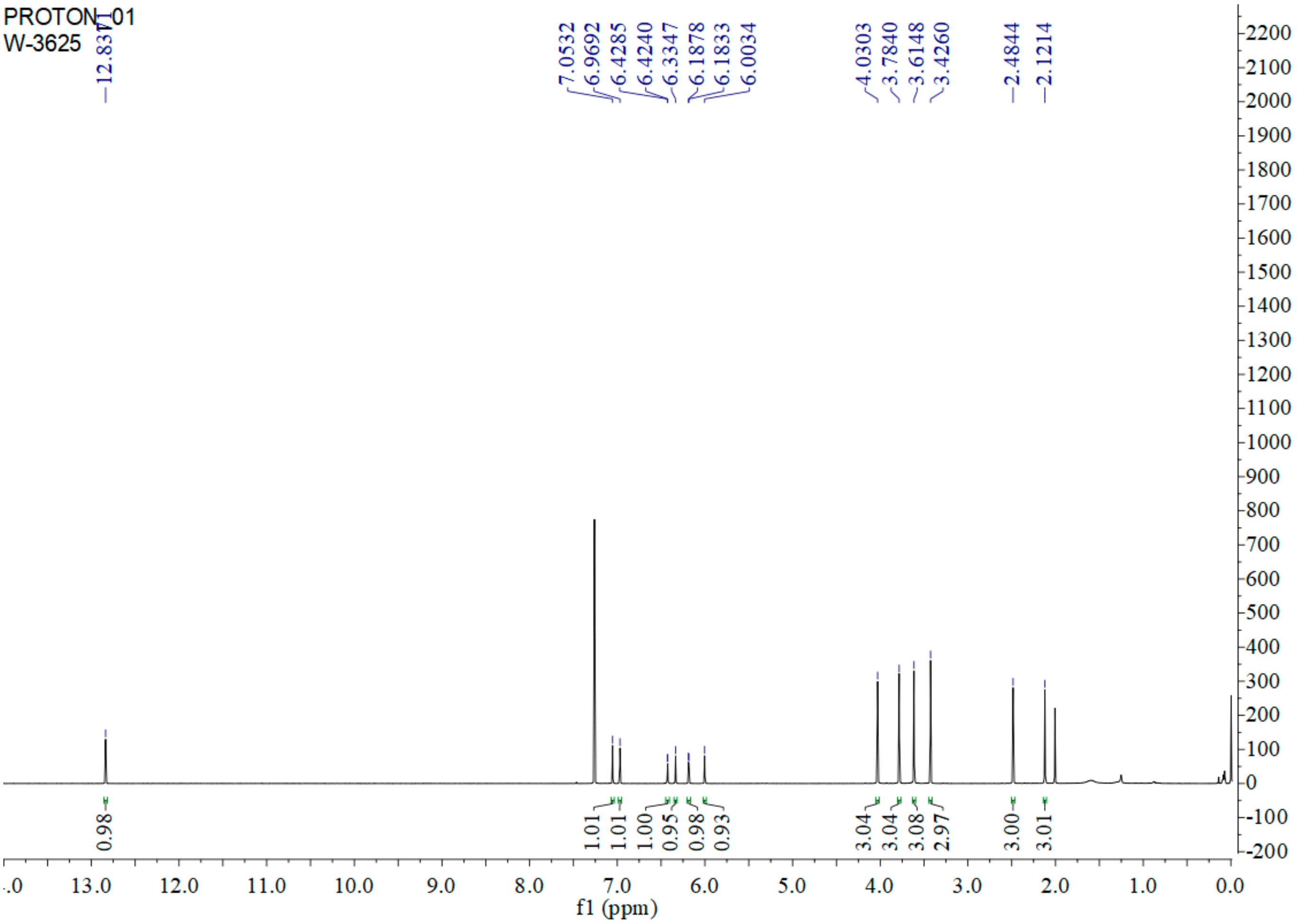

Figure S17. ^{1}H NMR spectrum (500 MHz) of compound **1** in $CDCl_3$.

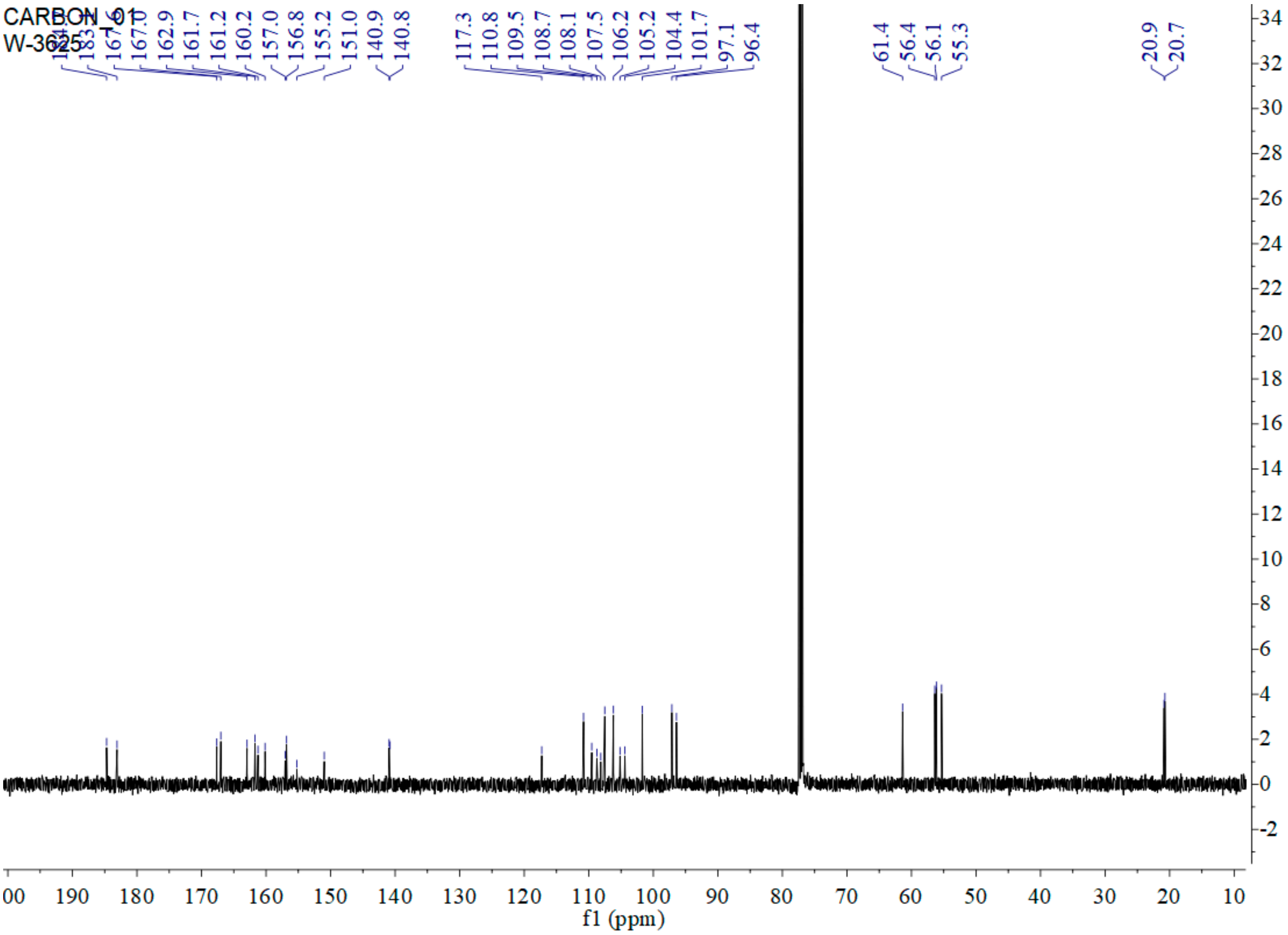

Figure S18. ^{13}C NMR spectrum (125 MHz) of compound **1** in $CDCl_3$.

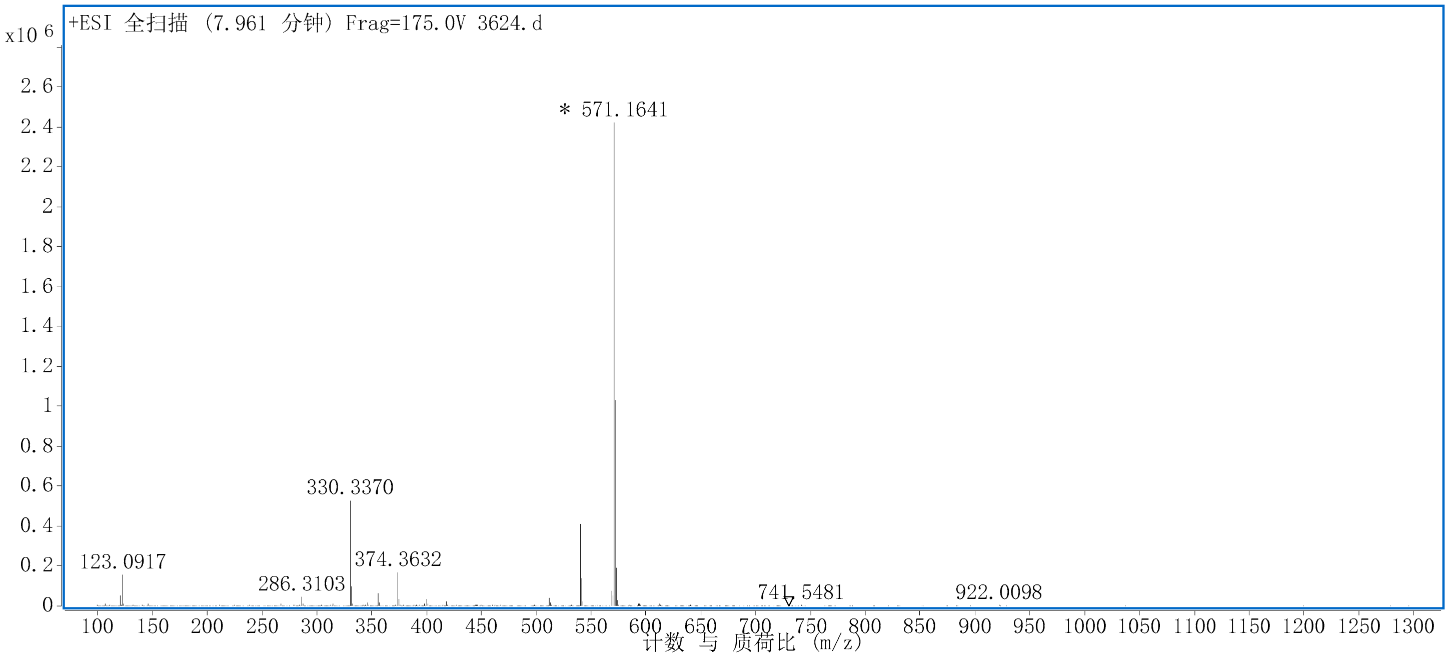

Figure S19. HRESIMS spectrum of compound **1**.

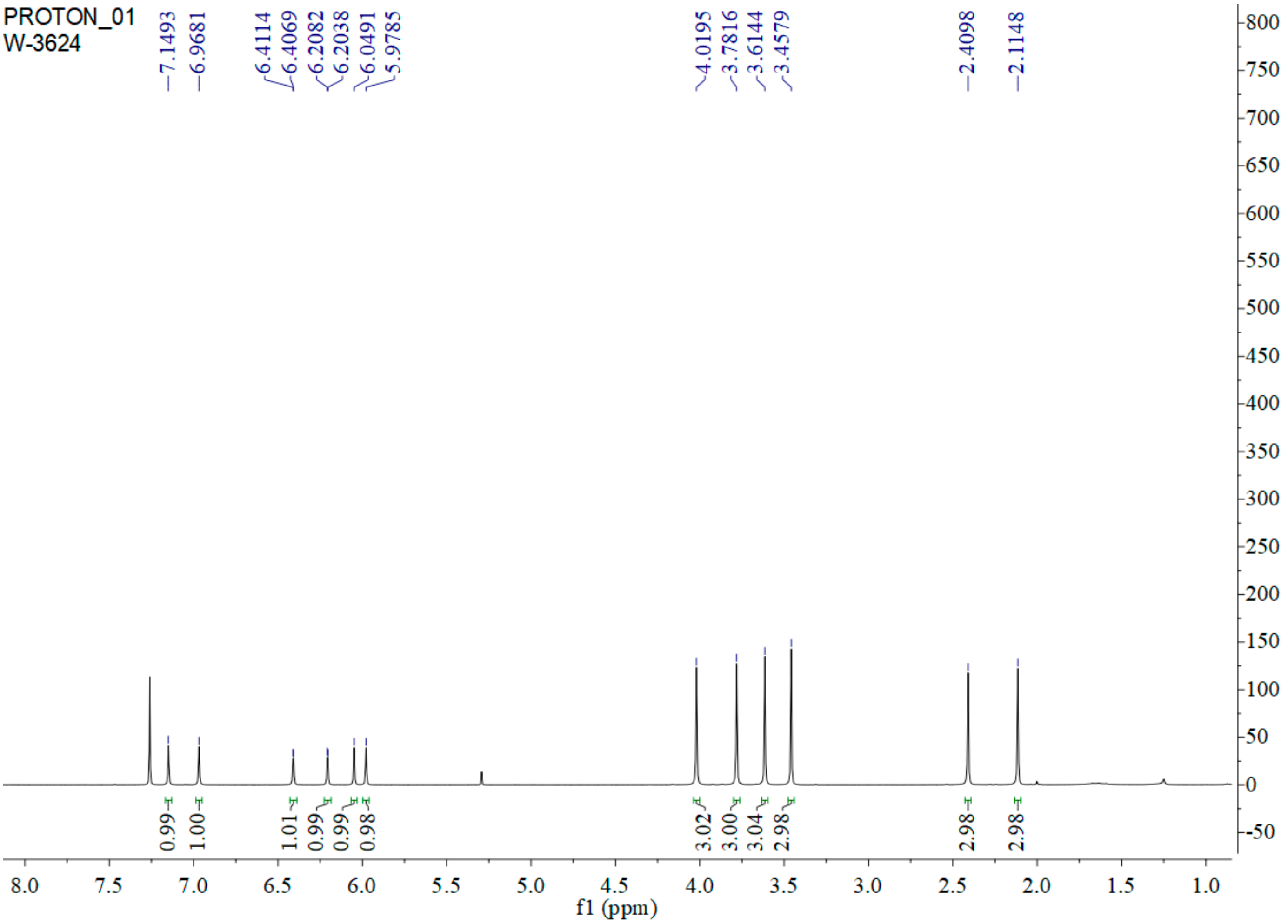

Figure S20. ^{1}H NMR spectrum (500 MHz) of compound **2** in $CDCl_3$.

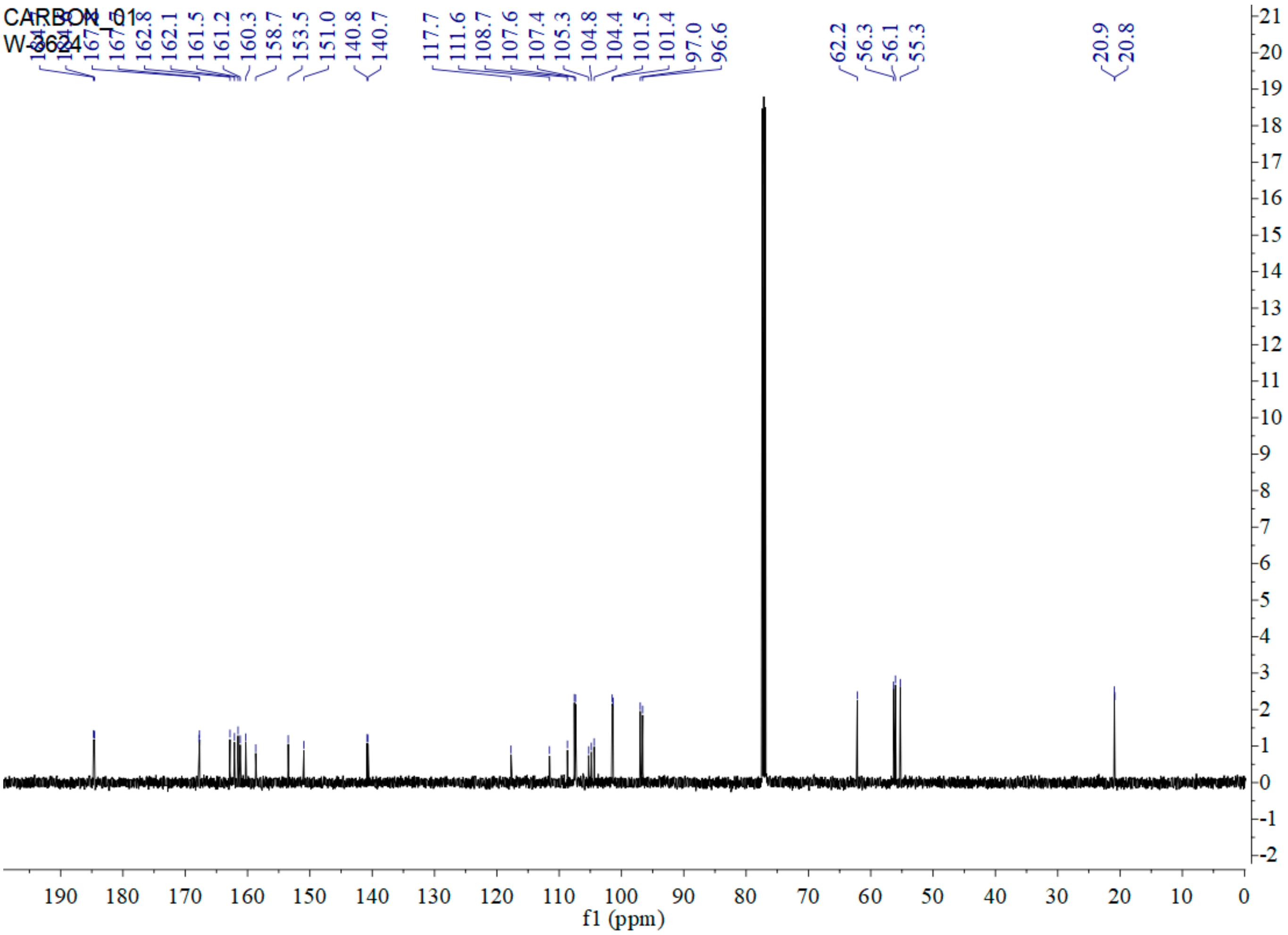

Figure S21. ^{13}C NMR spectrum (125 MHz) of compound **2** in $CDCl_3$.

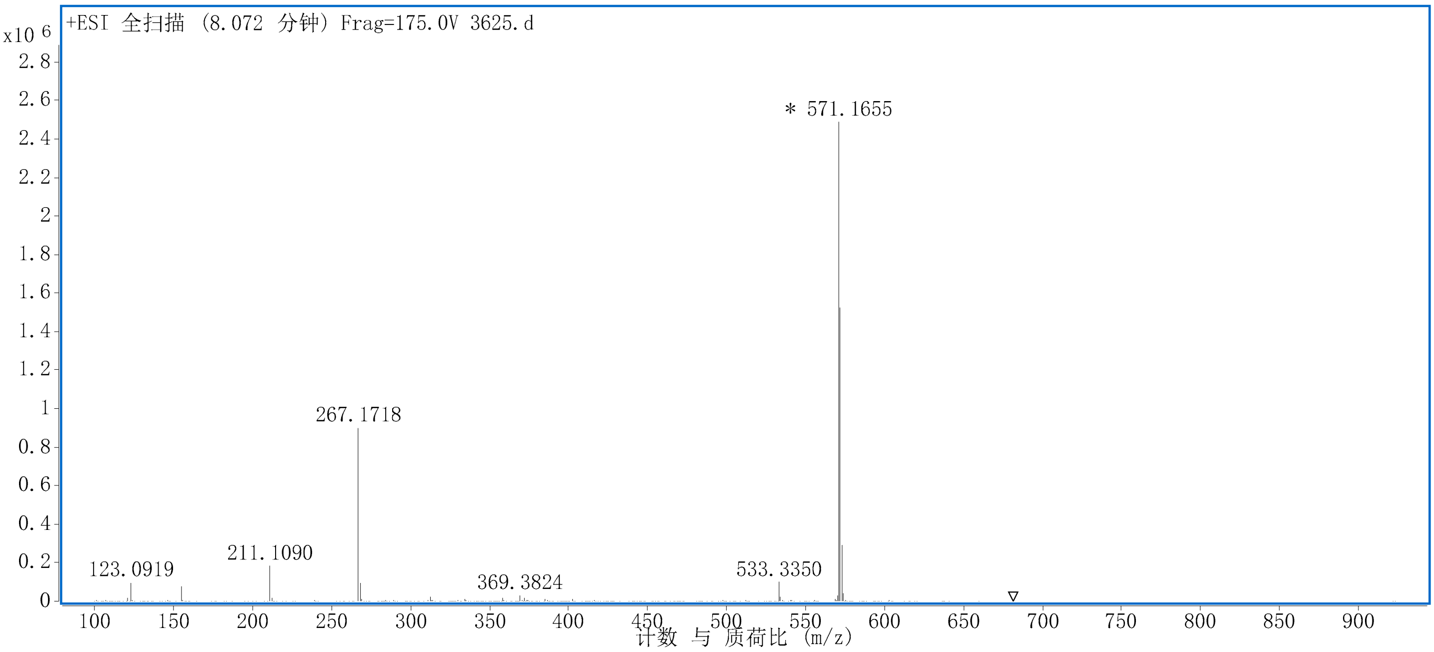

Figure S22. HRESIMS spectrum of compound **2**.

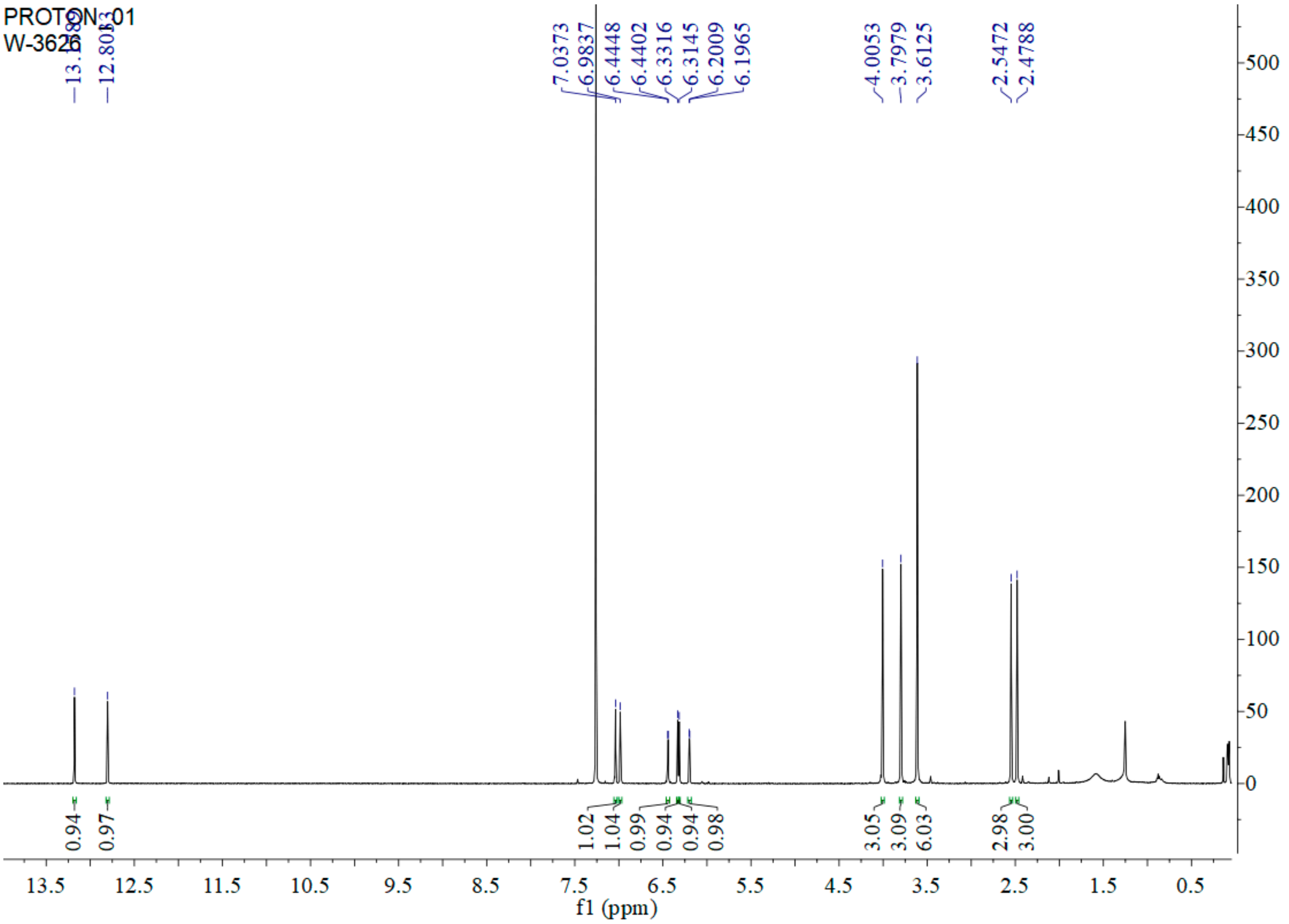

Figure S23. ^{1}H NMR spectrum (500 MHz) of compound **3** in $CDCl_3$.

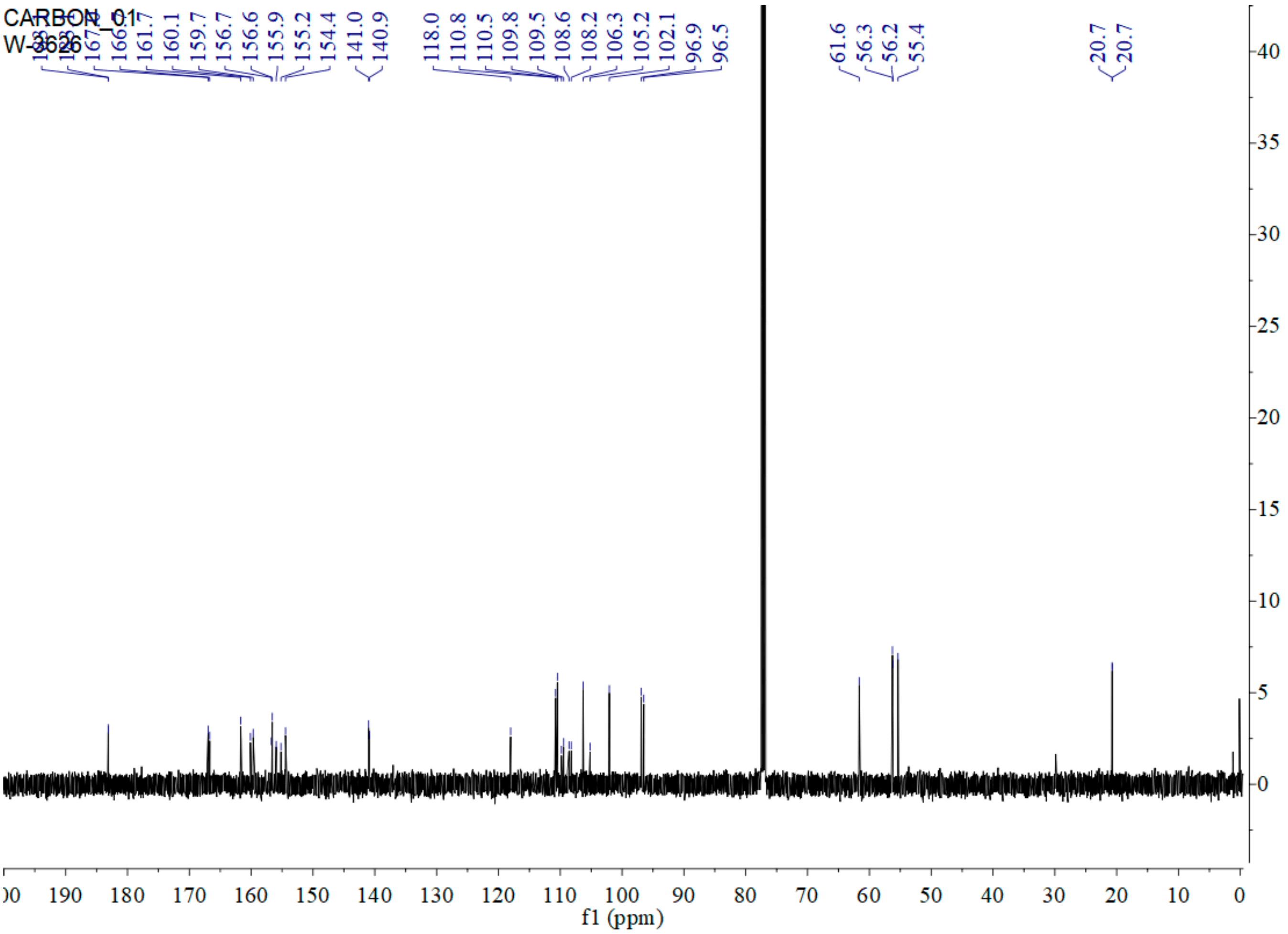

Figure S24. ^{13}C NMR spectrum (125 MHz) of compound **3** in $CDCl_3$.

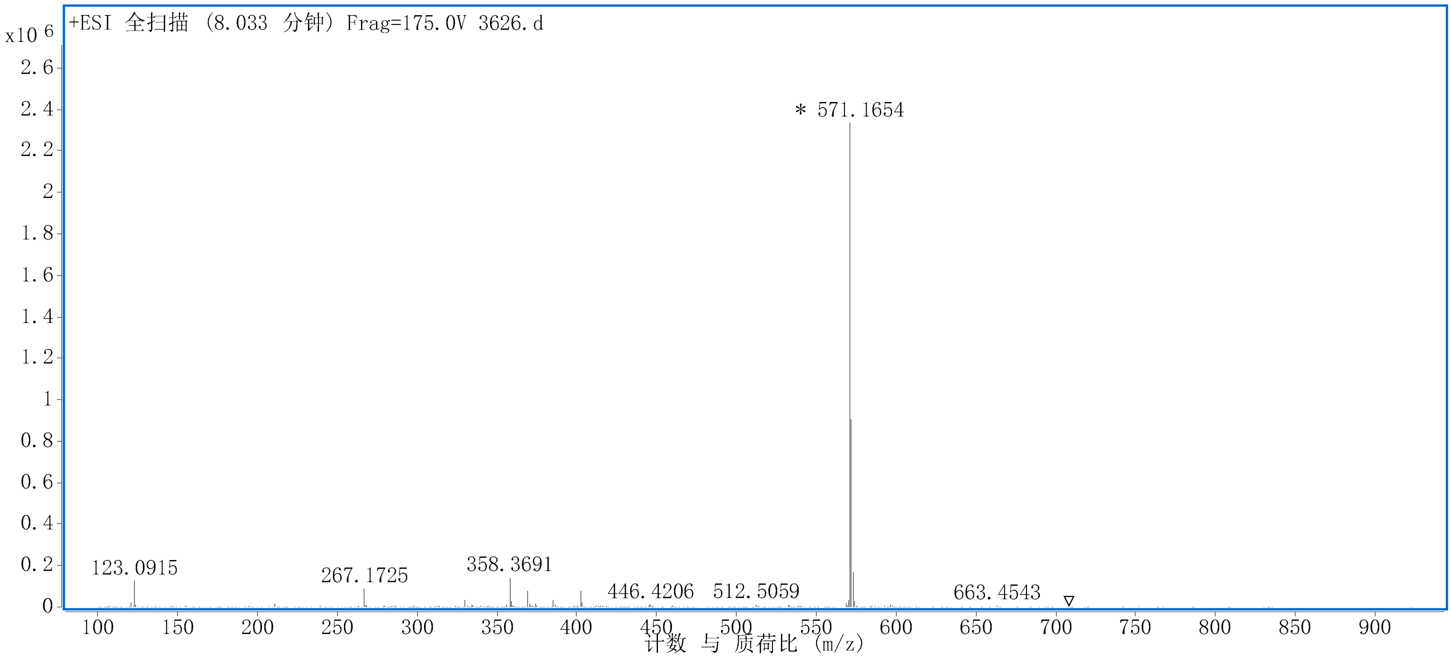

Figure S25. HRESIMS spectrum of compound **3**.

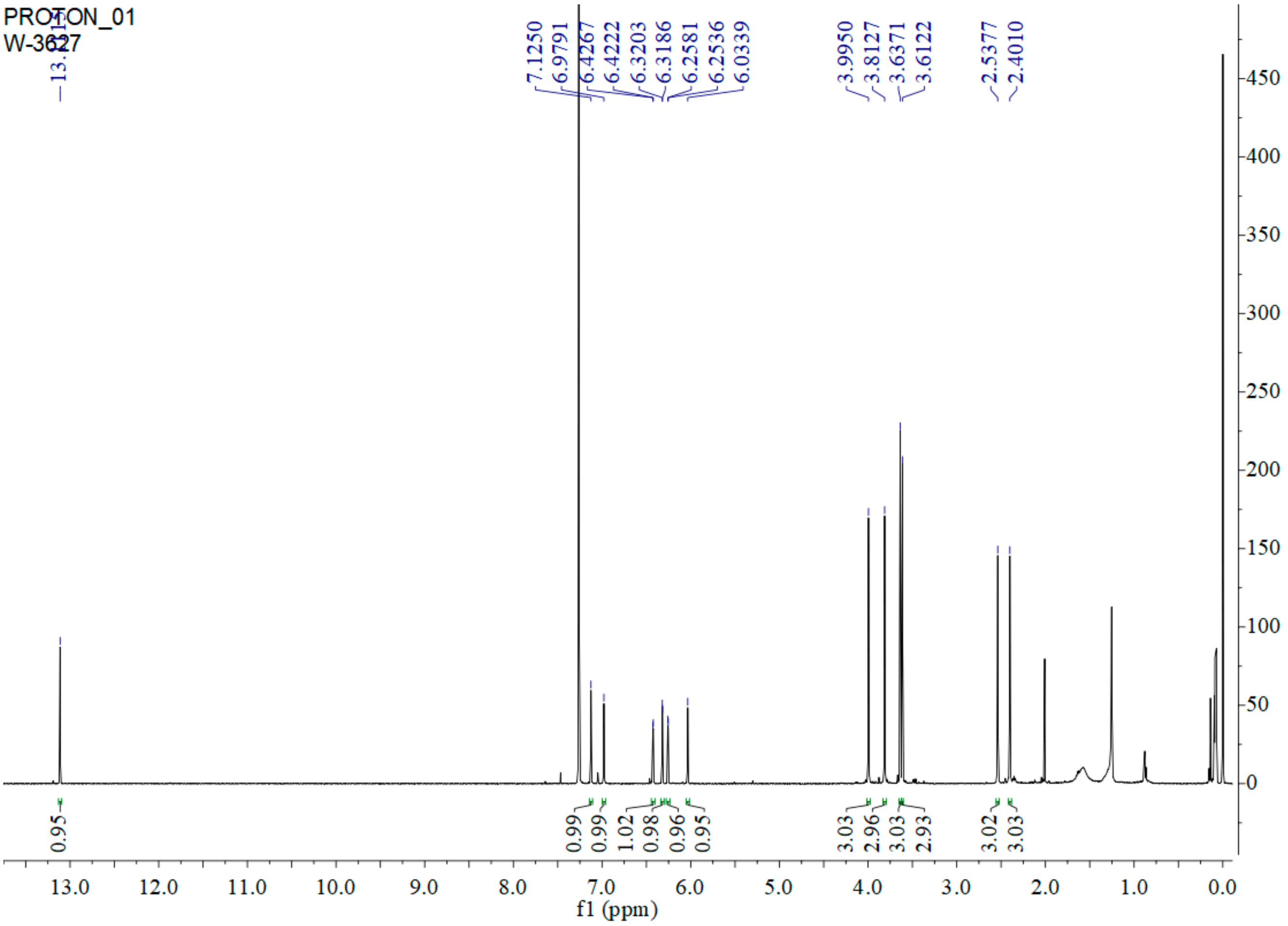

Figure S26. ^{1}H NMR spectrum (500 MHz) of compound **4** in $CDCl_3$.

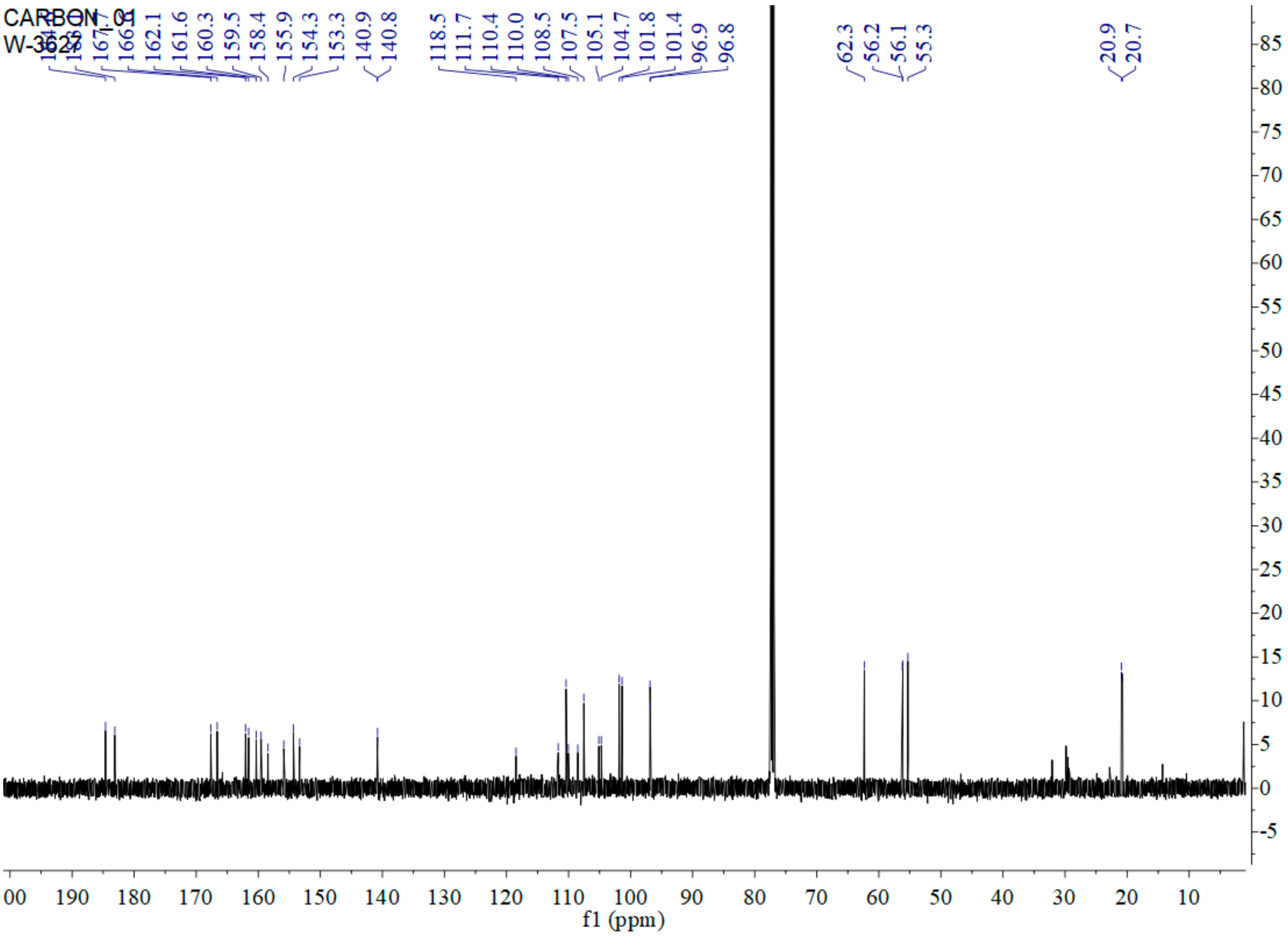

Figure S27. ^{13}C NMR spectrum (125 MHz) of compound **4** in $CDCl_3$.

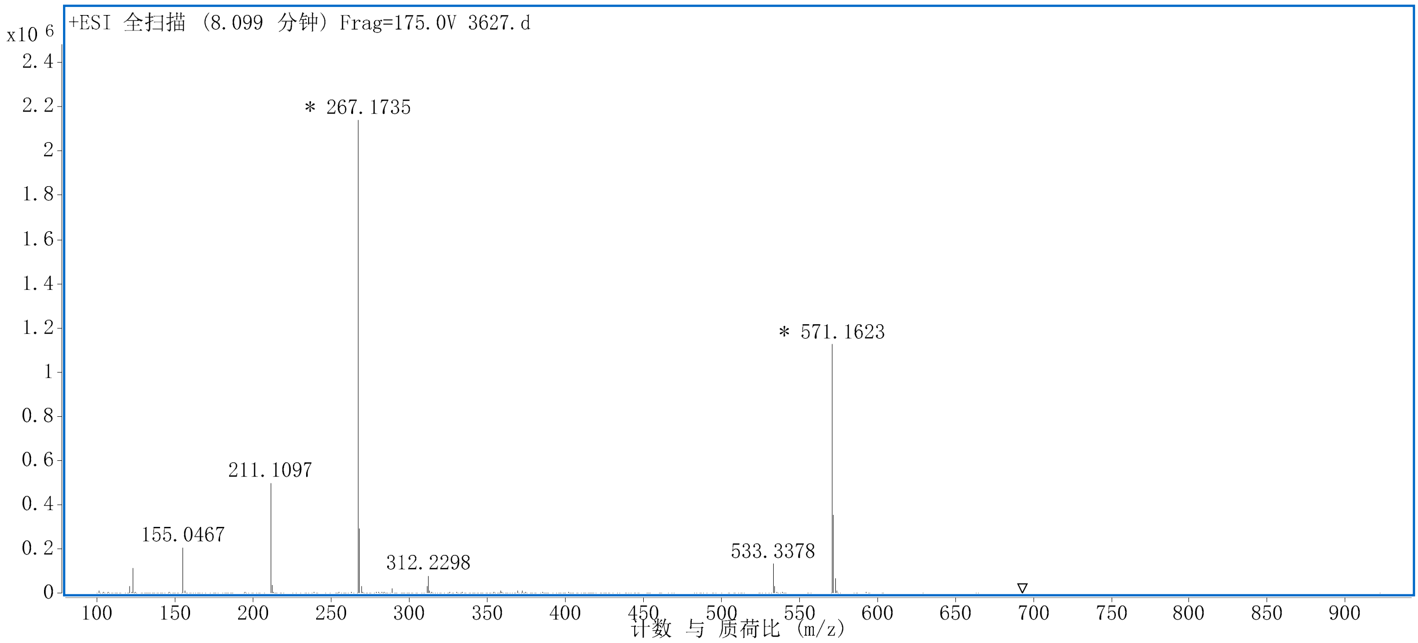

Figure S28. HRESIMS spectrum of compound **4**.

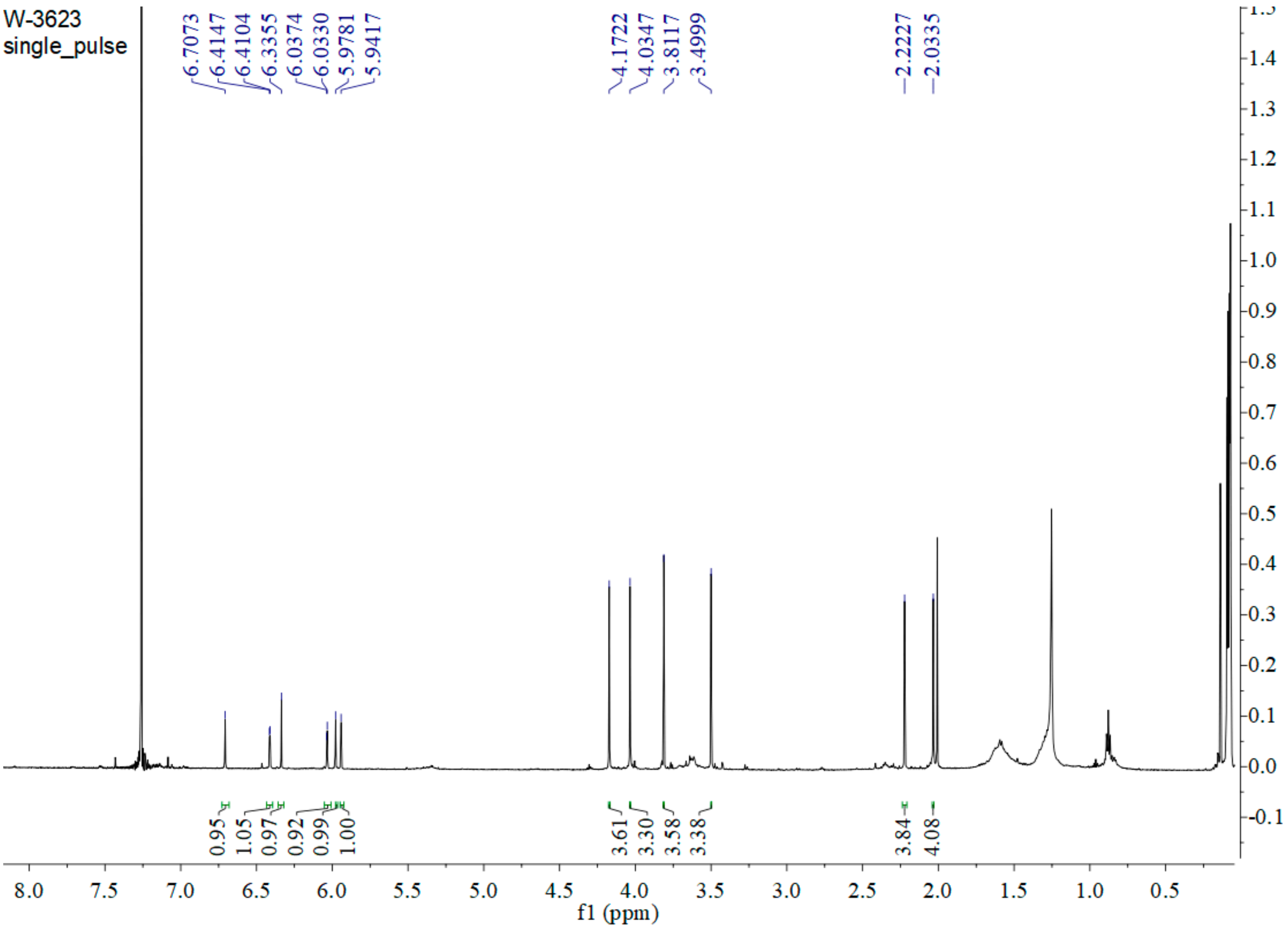

Figure S29. ^{1}H NMR spectrum (600 MHz) of compound **5** in $CDCl_3$.

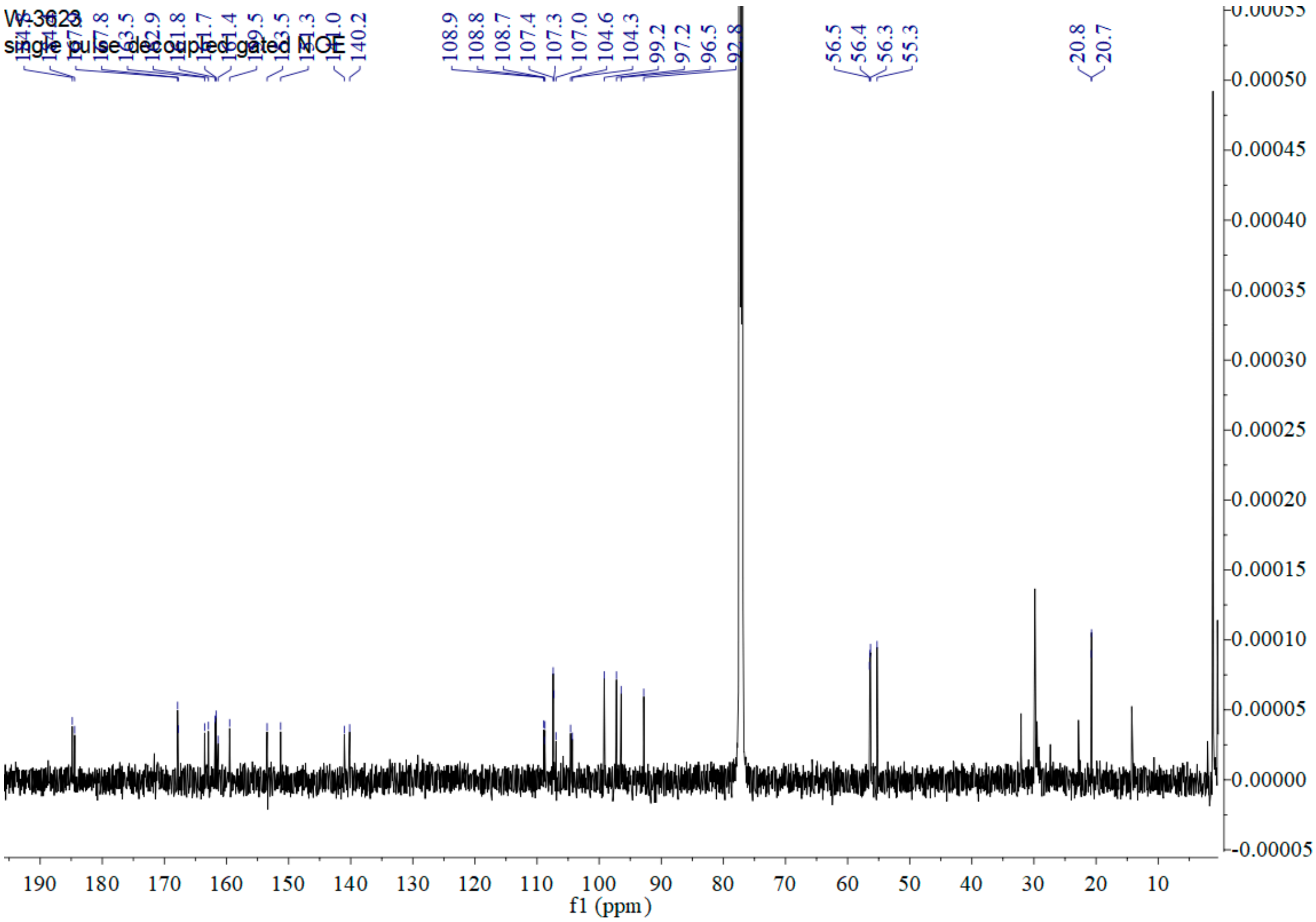

Figure S30. ^{13}C NMR spectrum (150 MHz) of compound **5** in $CDCl_3$.

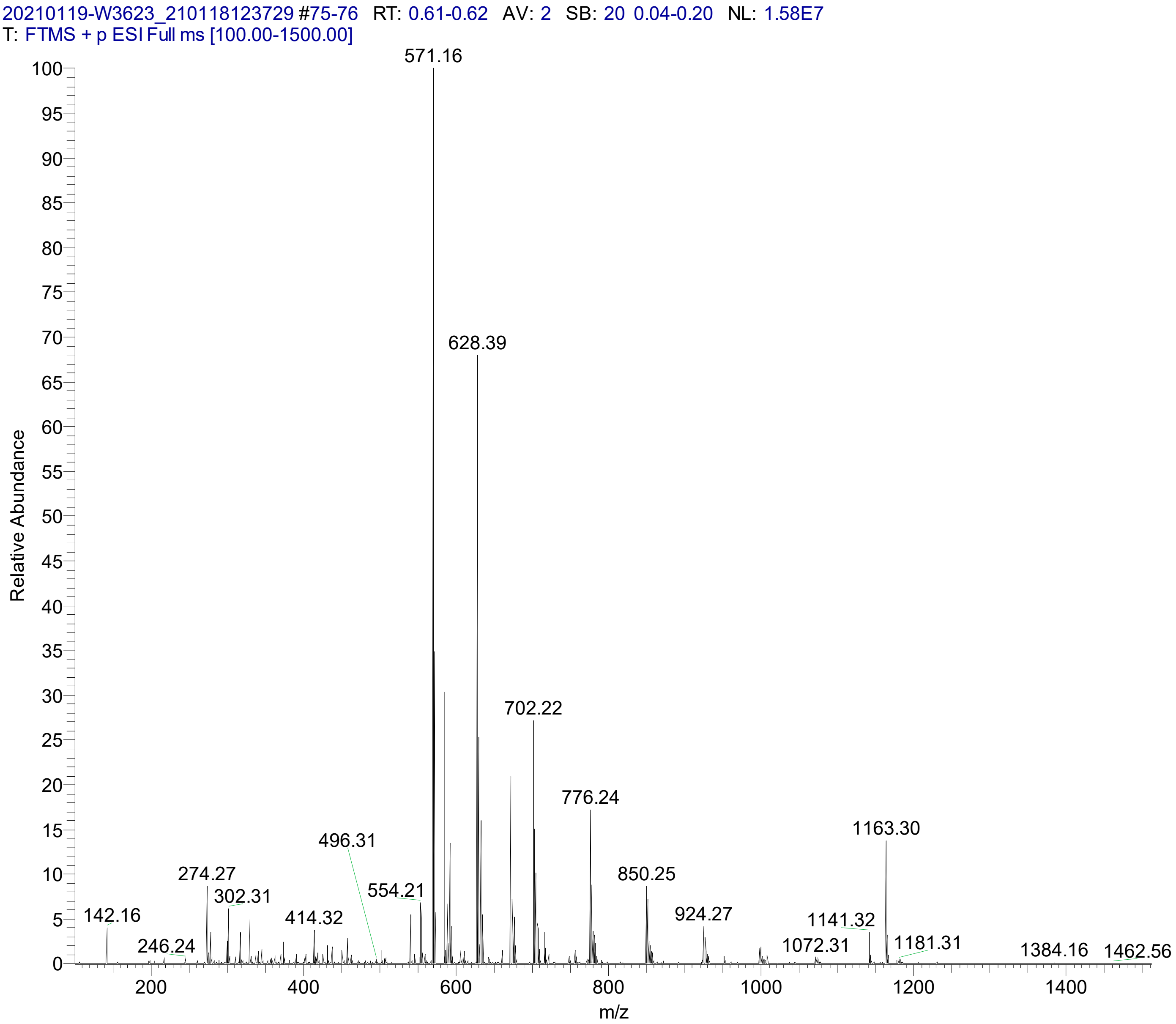

Figure S31. ESIMS spectrum of compound **5**.

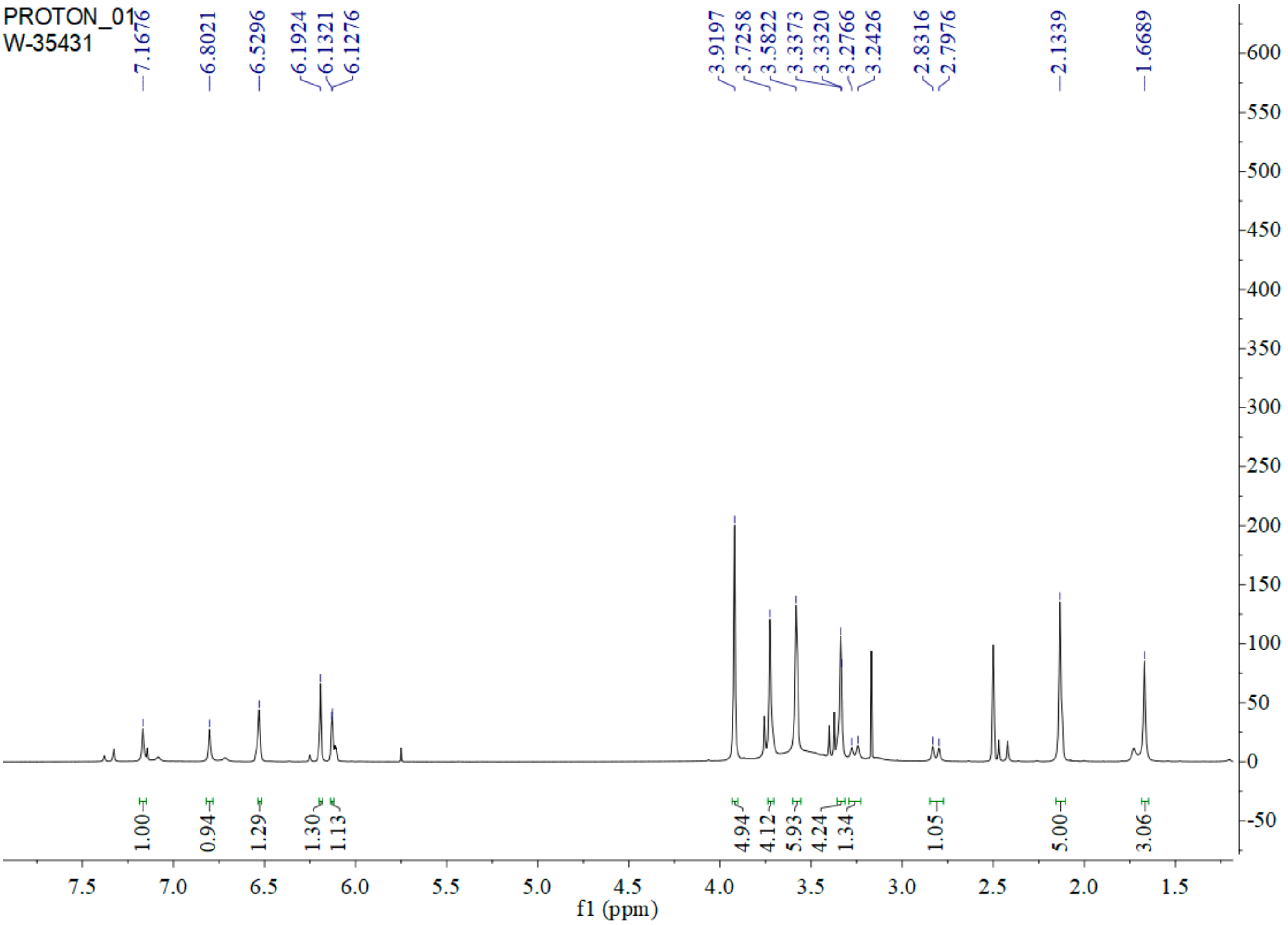

Figure S32. ^{1}H NMR spectrum (500 MHz) of compound **6** in DMSO-d_6.

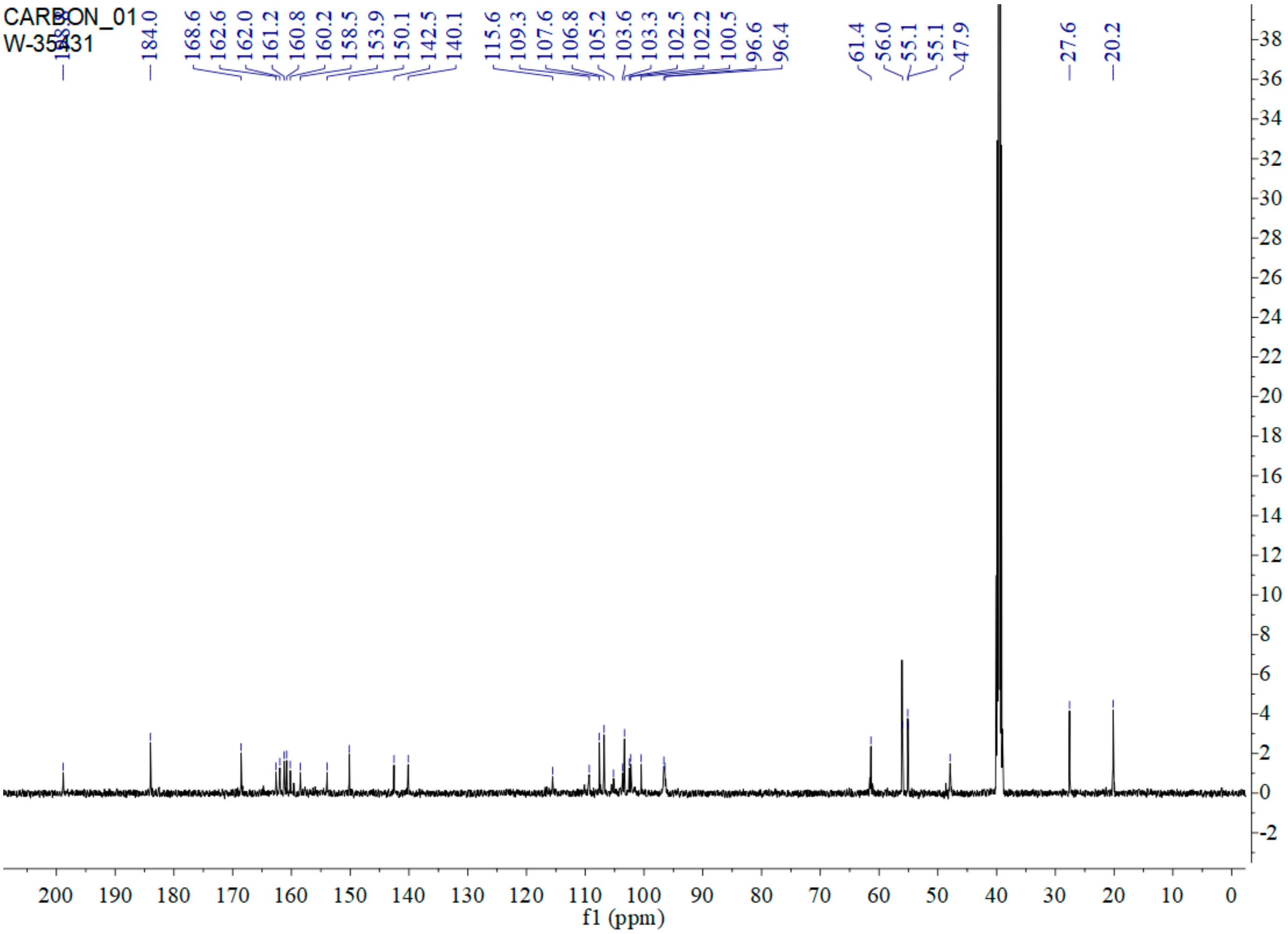

Figure S33. ^{13}C NMR spectrum (125 MHz) of compound **6** in DMSO-d_6.

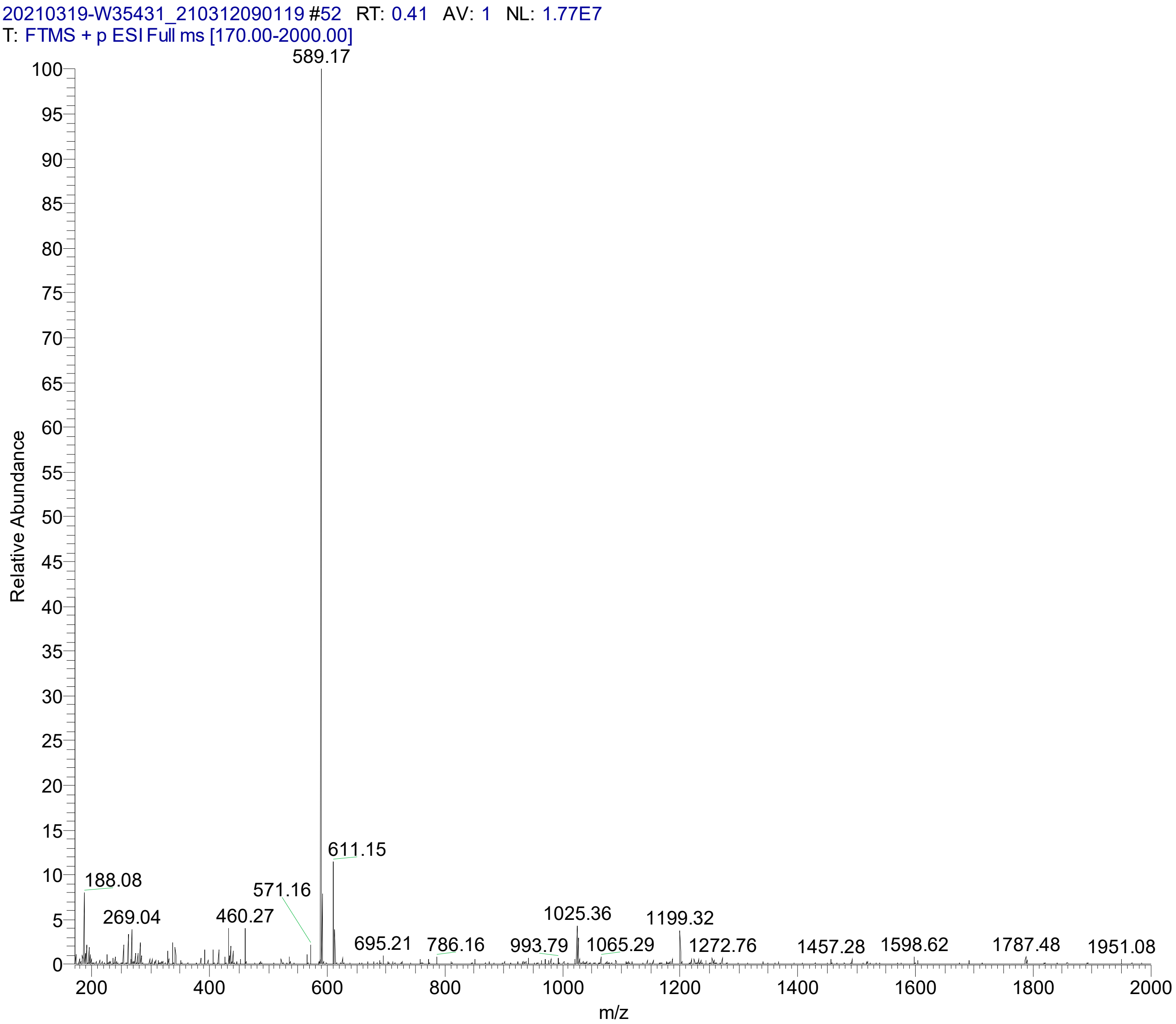

Figure S34. ESIMS spectrum of compound **6**.

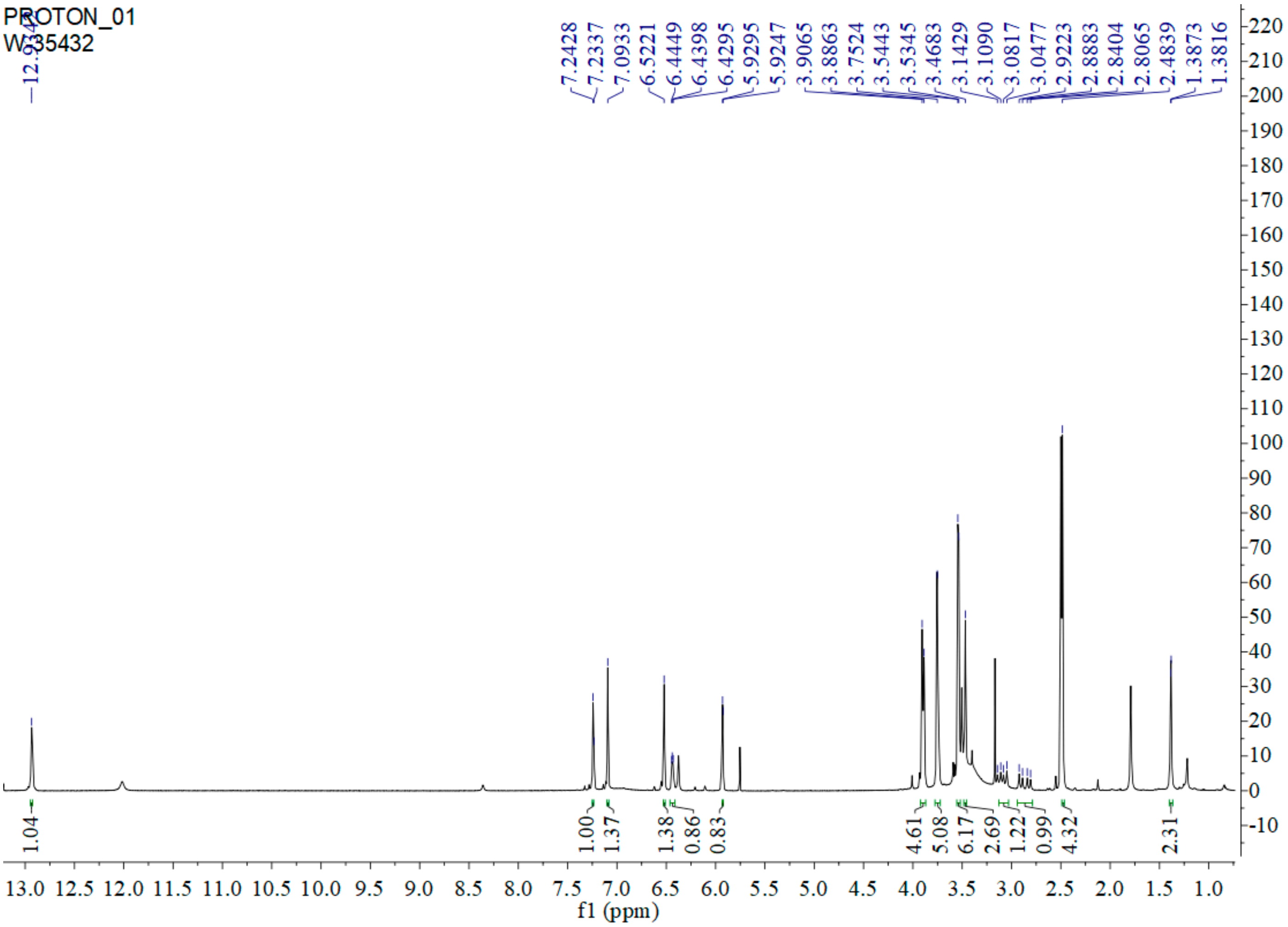

Figure S35. ^{1}H NMR spectrum (500 MHz) of compound **7** in DMSO-d_6.

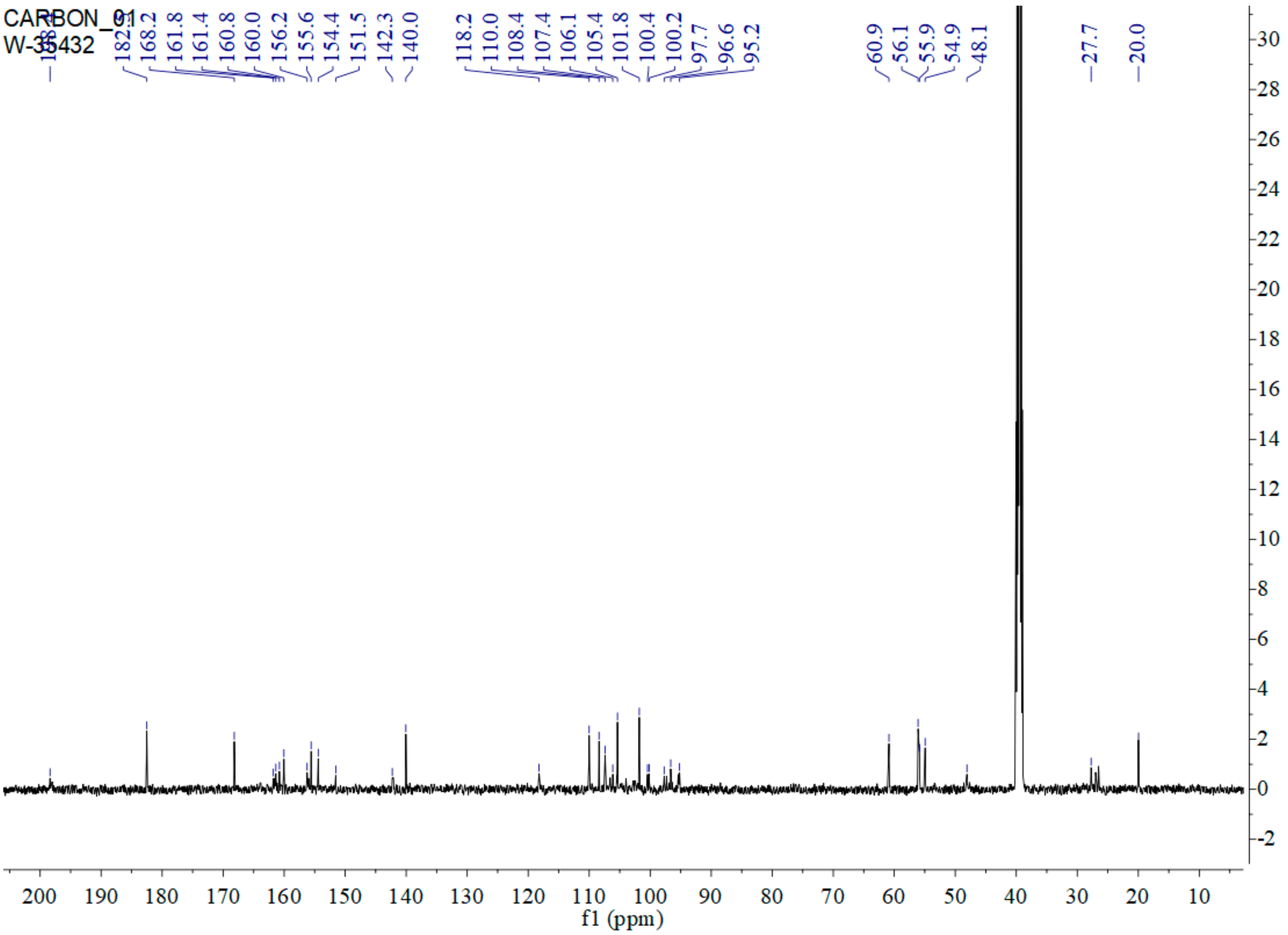

Figure S36. ^{13}C NMR spectrum (125 MHz) of compound **7** in DMSO-d_6.

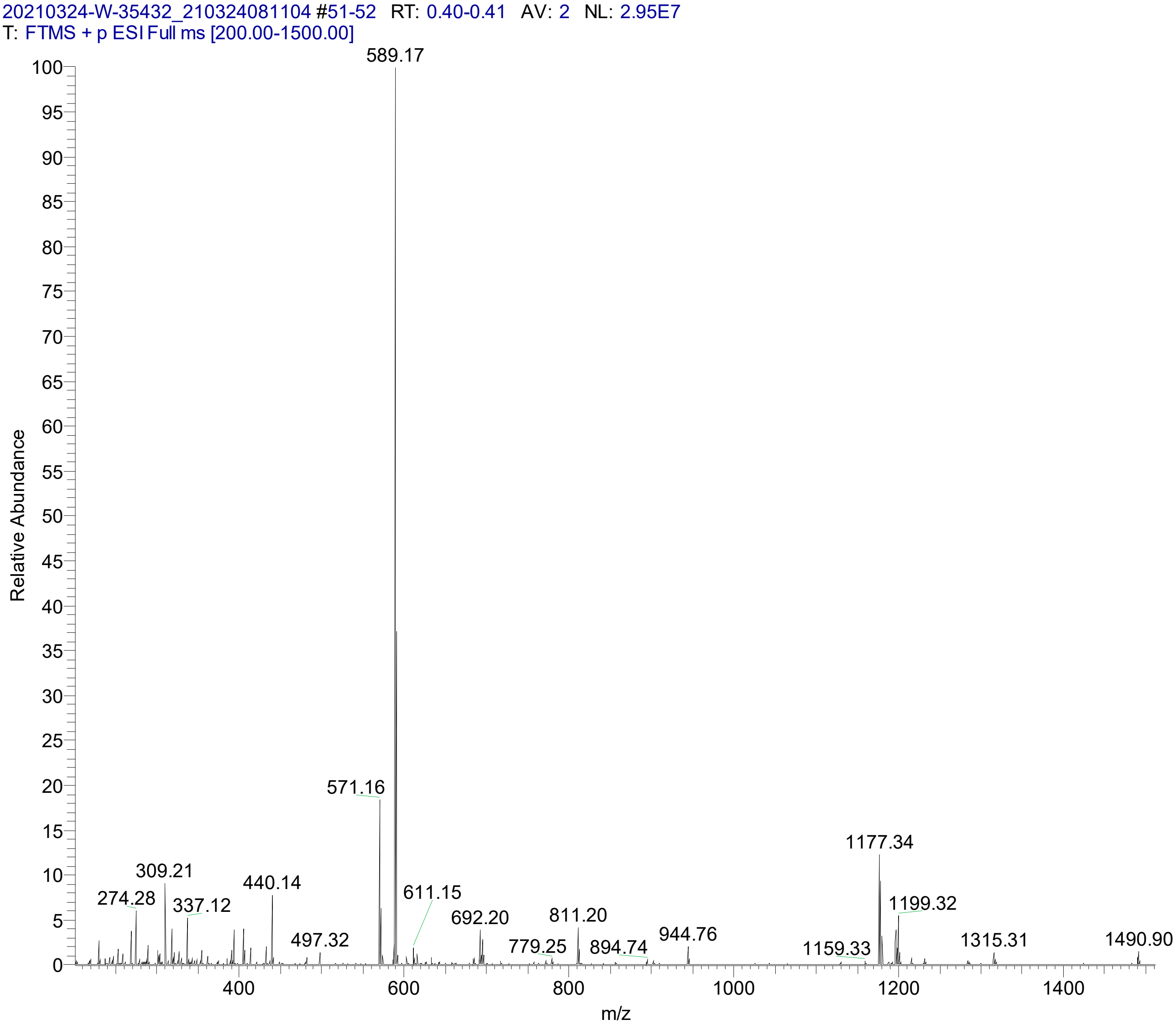

Figure S37. ESIMS spectrum of compound **7**.

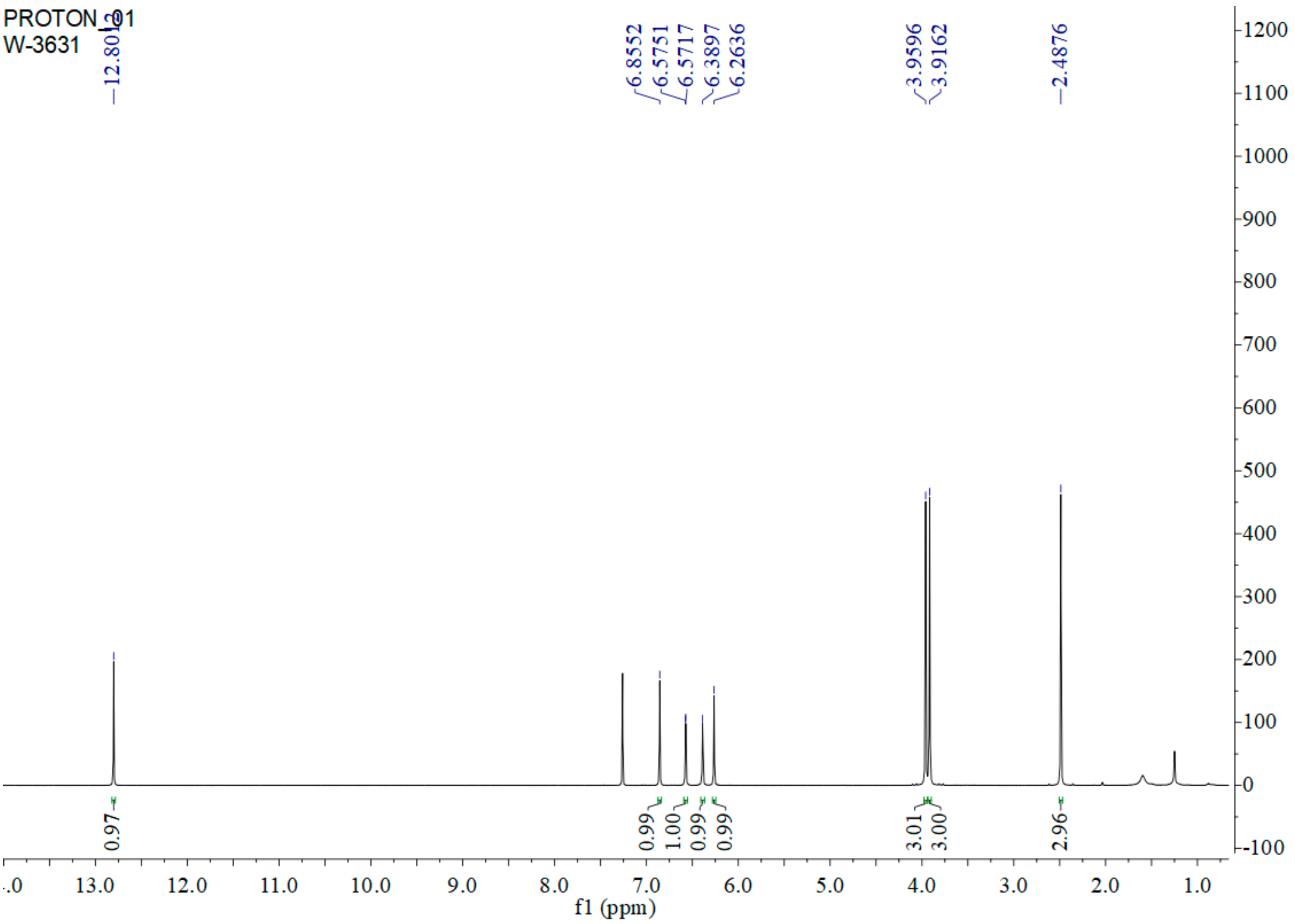

Figure S38. ^{1}H NMR spectrum (500 MHz) of compound **8** in $CDCl_3$.

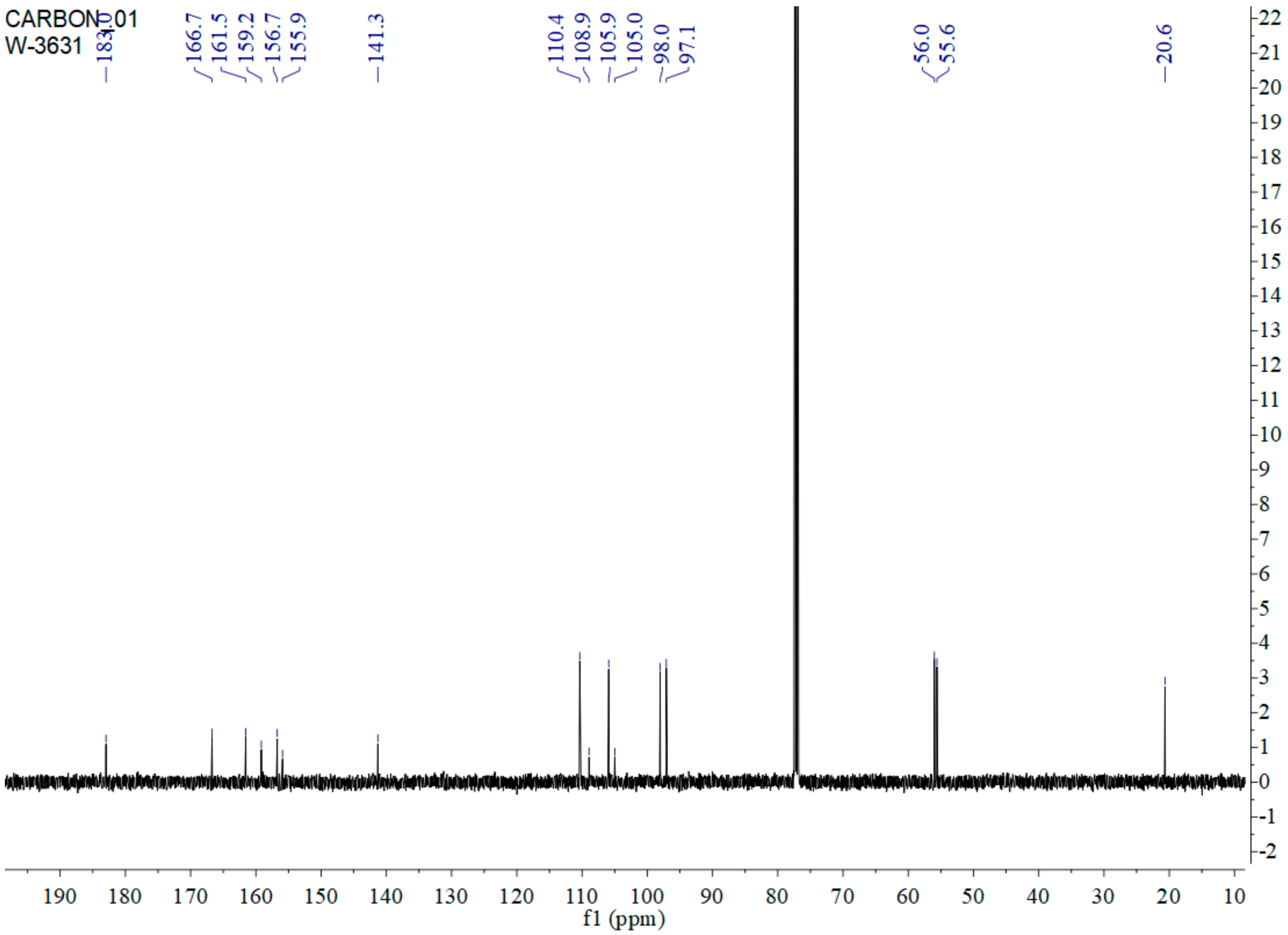

Figure S39. ^{13}C NMR spectrum (125 MHz) of compound **8** in $CDCl_3$.

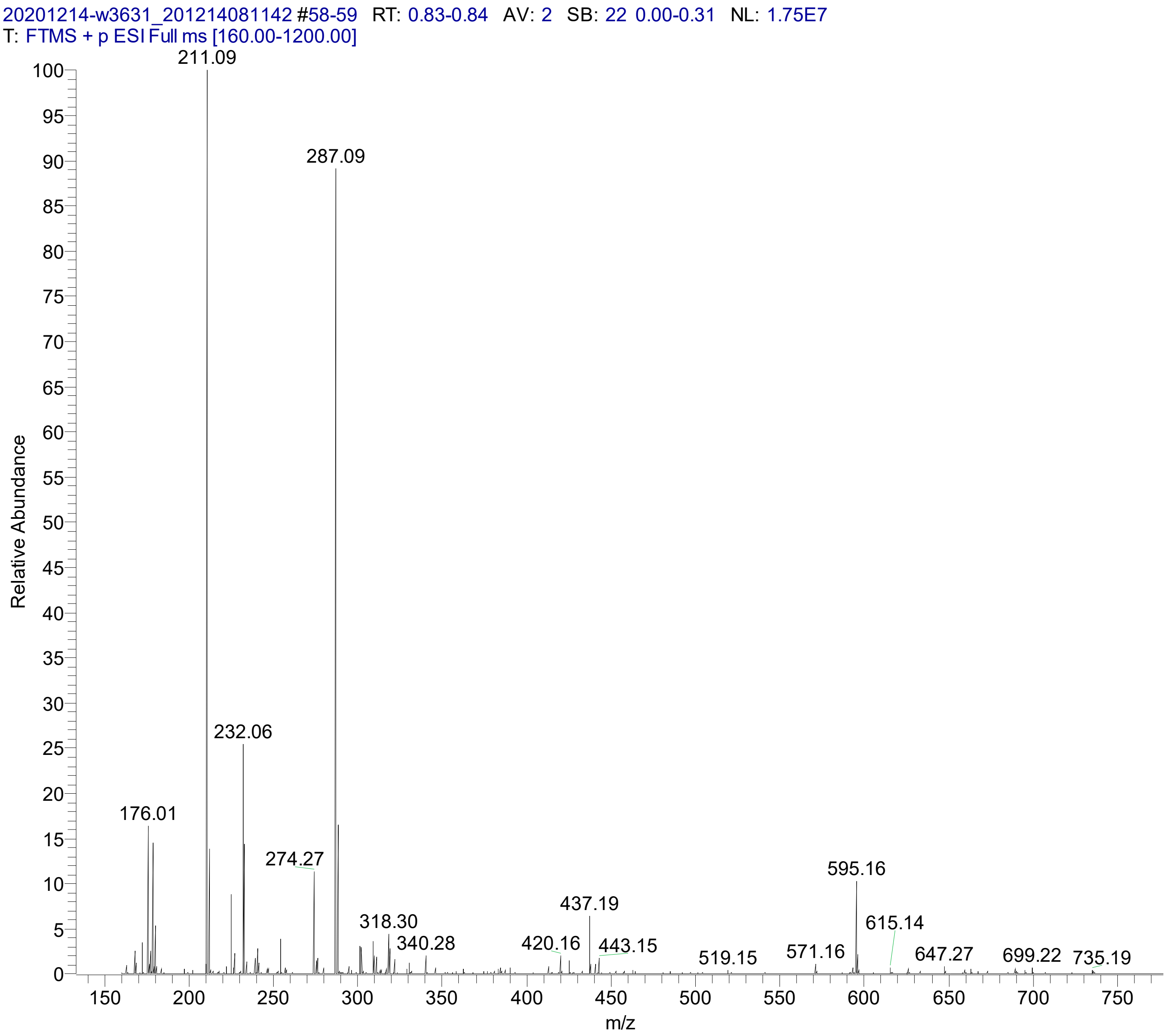

Figure S40. ESIMS spectrum of compound **8**.

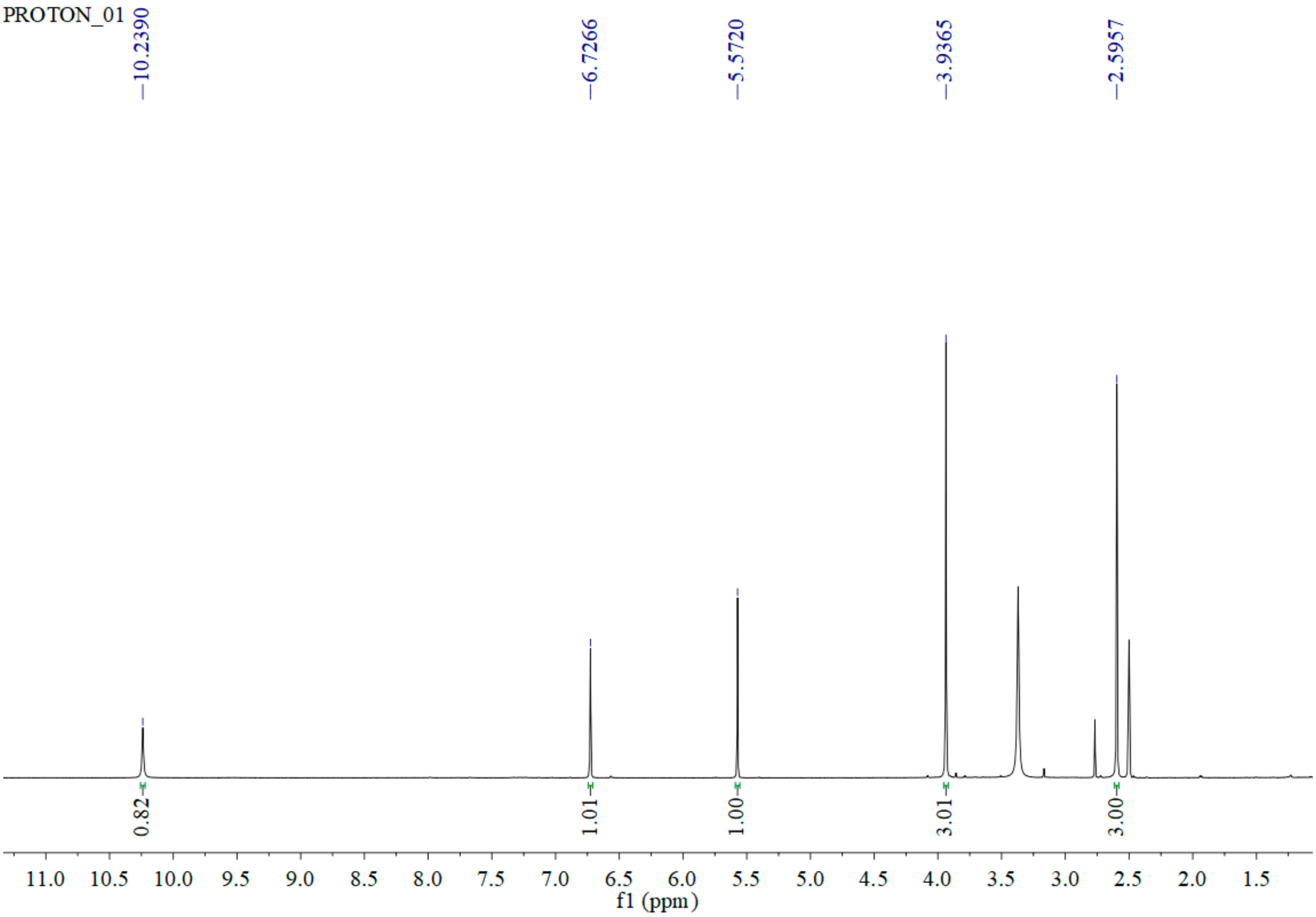

Figure S41. ^{1}H NMR spectrum (500 MHz) of compound **9** in DMSO-d_6.

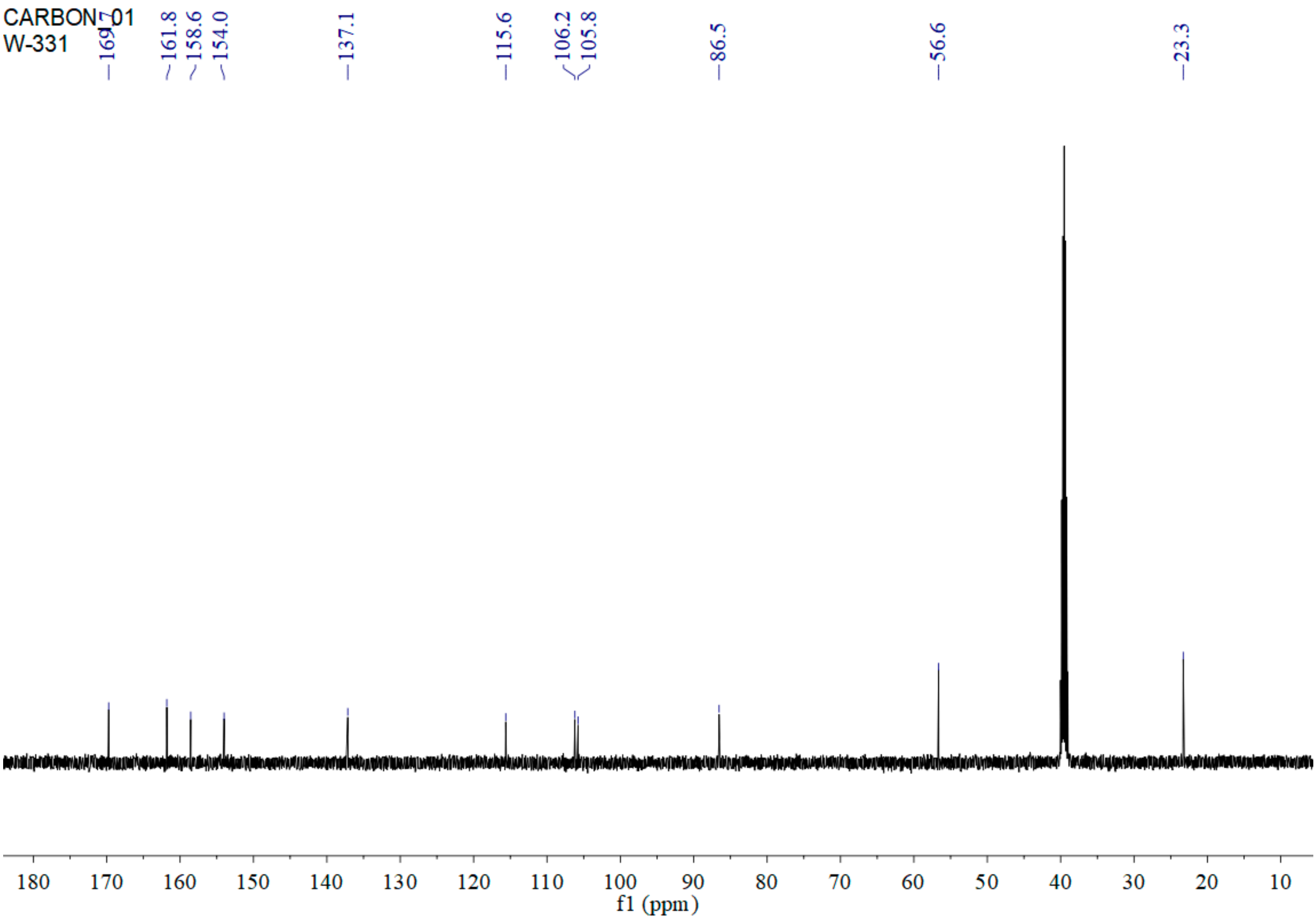

Figure S42. ^{13}C NMR spectrum (125 MHz) of compound **9** in DMSO-d_6.

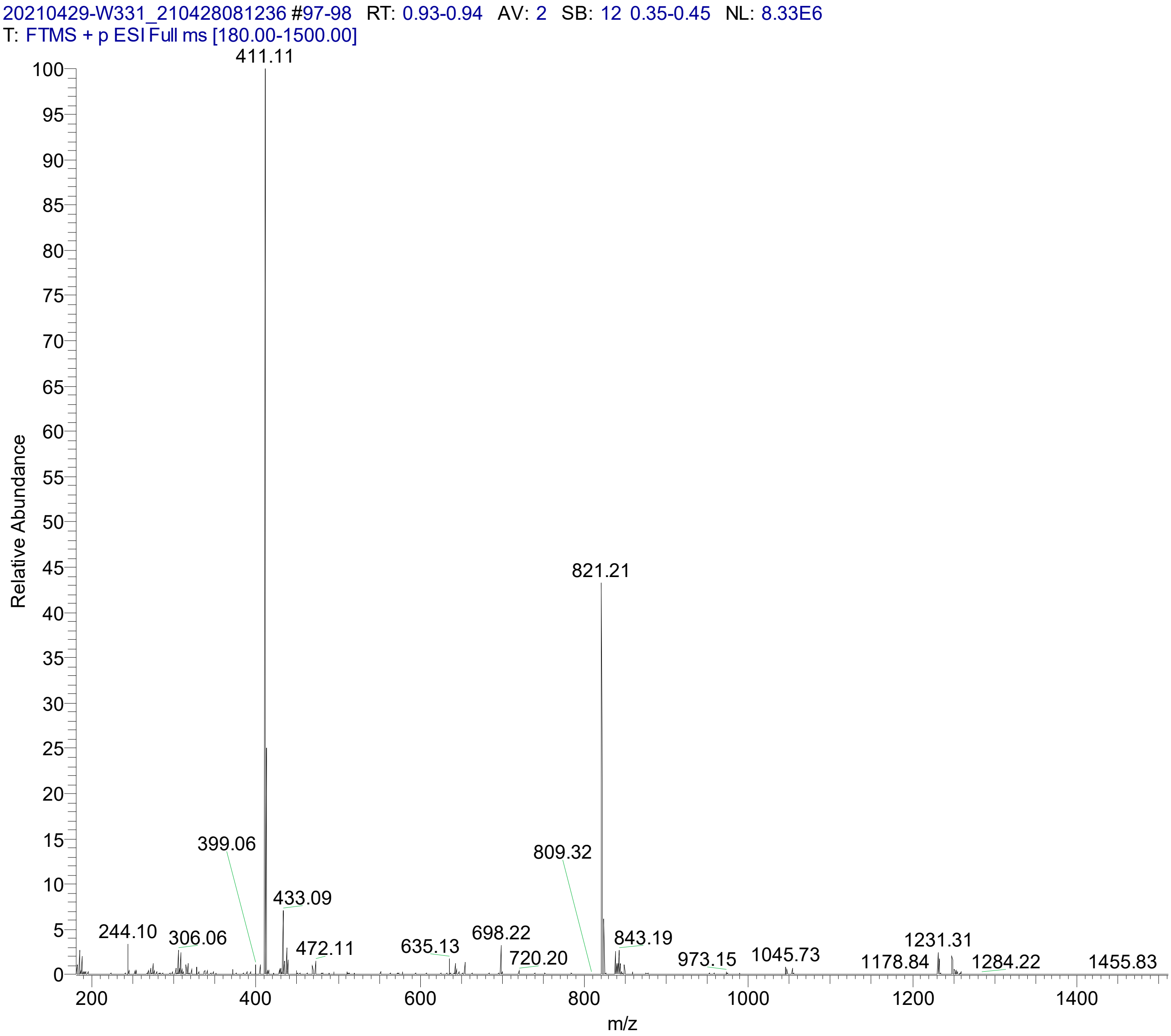

Figure S43. ESIMS spectrum of compound **9**.

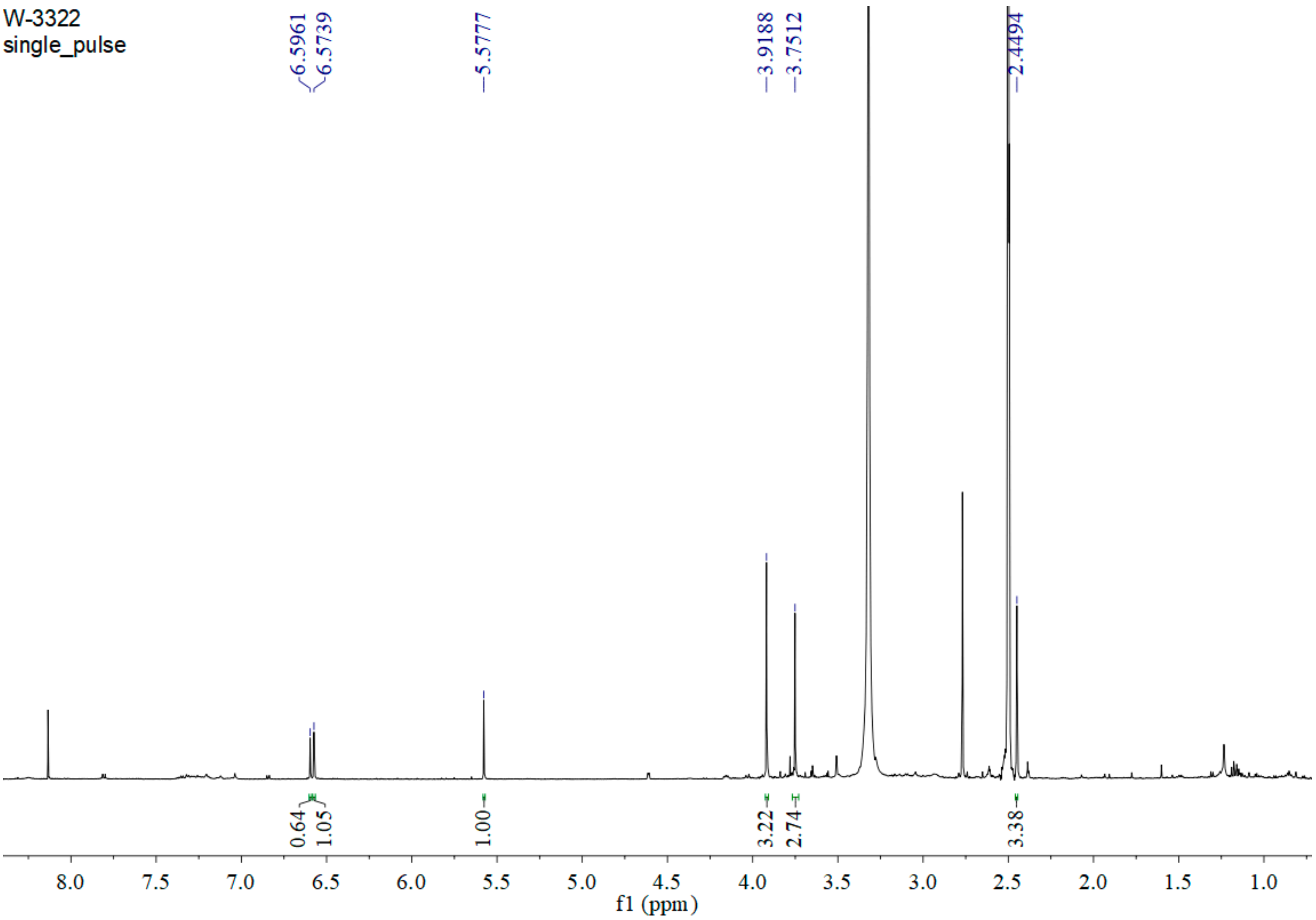

Figure S44. ^{1}H NMR spectrum (600 MHz) of compound **10** in DMSO-d_6.

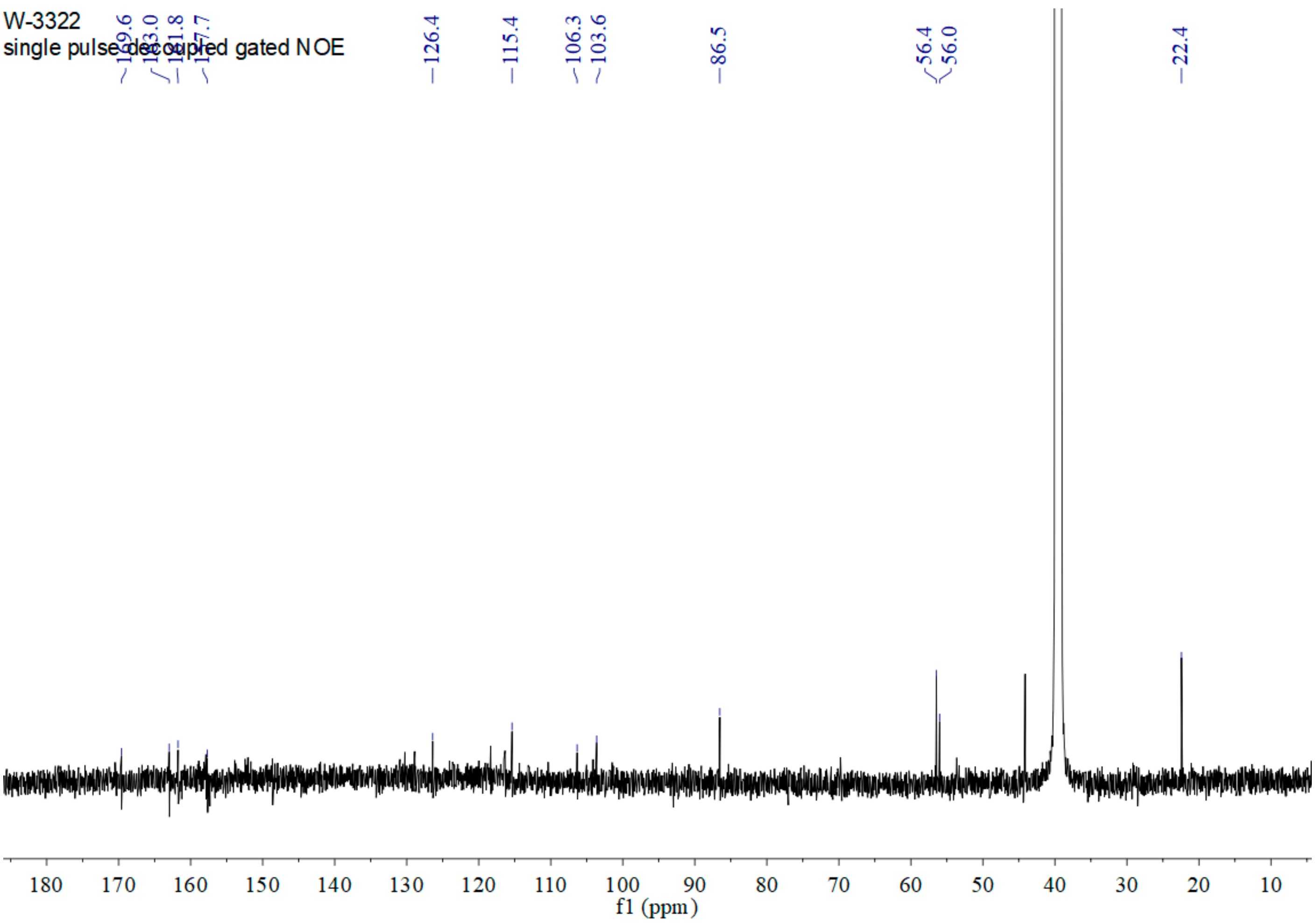

Figure S45. ^{13}C NMR spectrum (150 MHz) of compound **10** in DMSO-d_6.

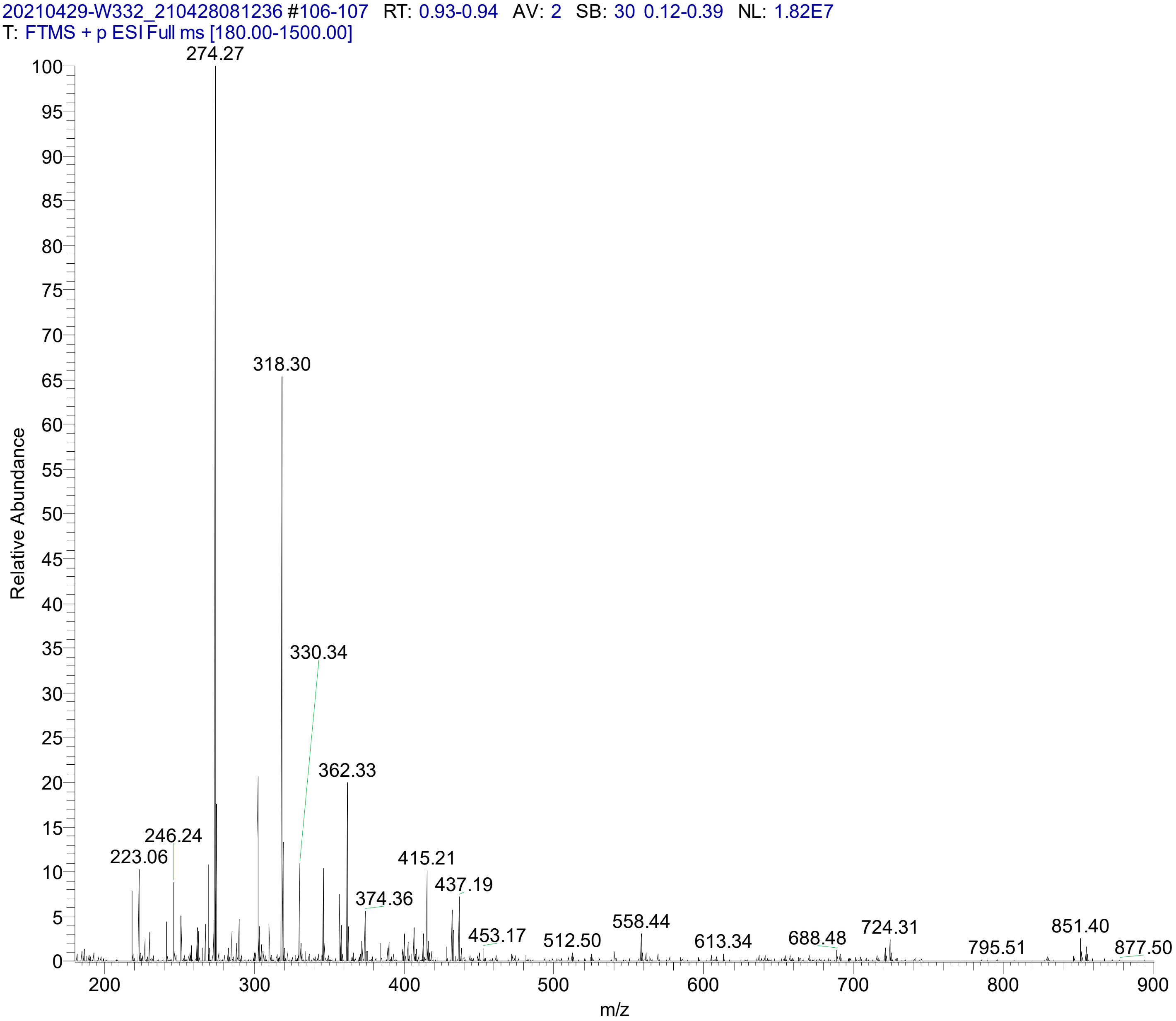

Figure S46. ESIMS spectrum of compound **10**.

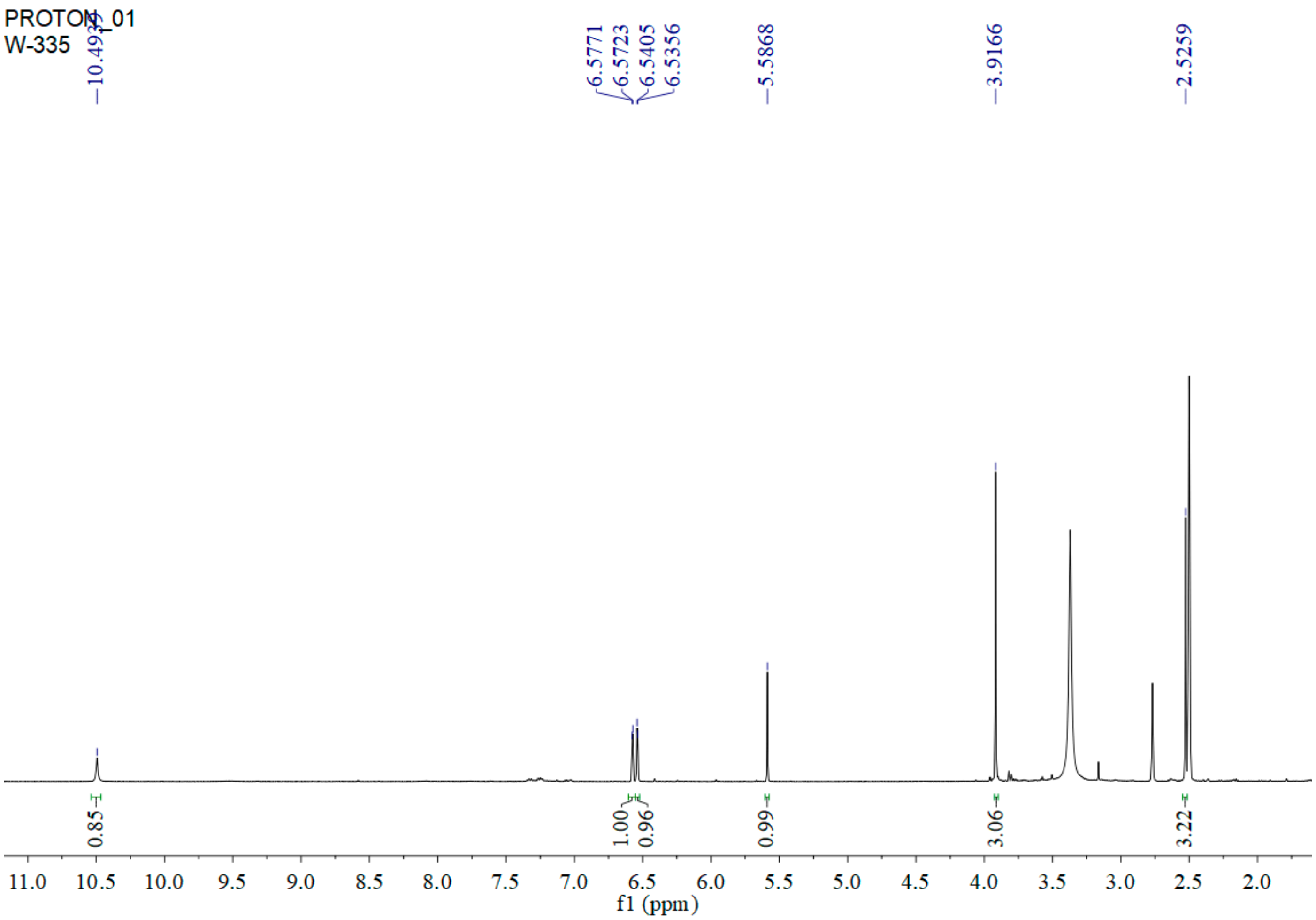

Figure S47. ^{1}H NMR spectrum (500 MHz) of compound **11** in DMSO-d_6.

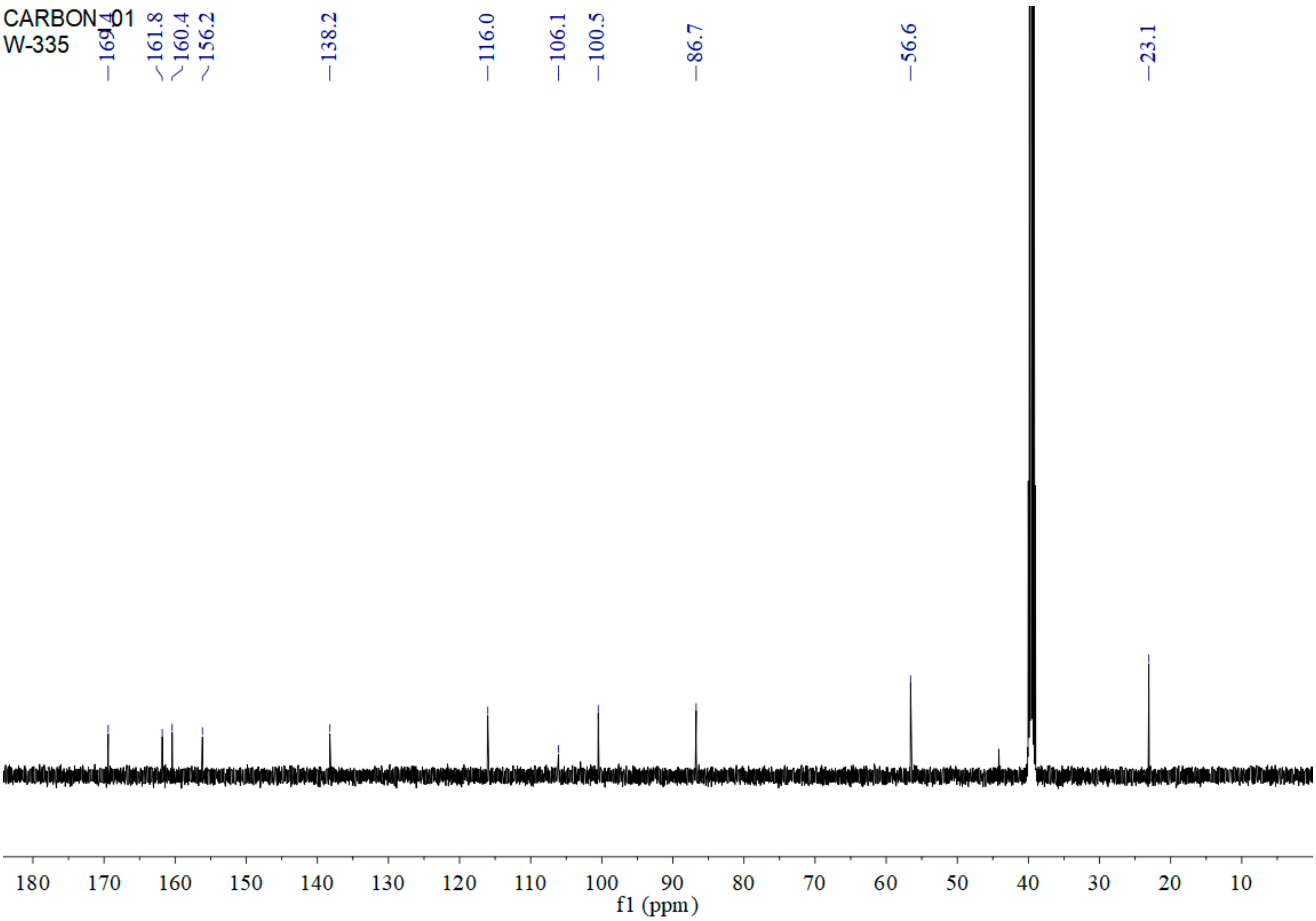

Figure S48. ^{13}C NMR spectrum (125 MHz) of compound **11** in DMSO-d_6.

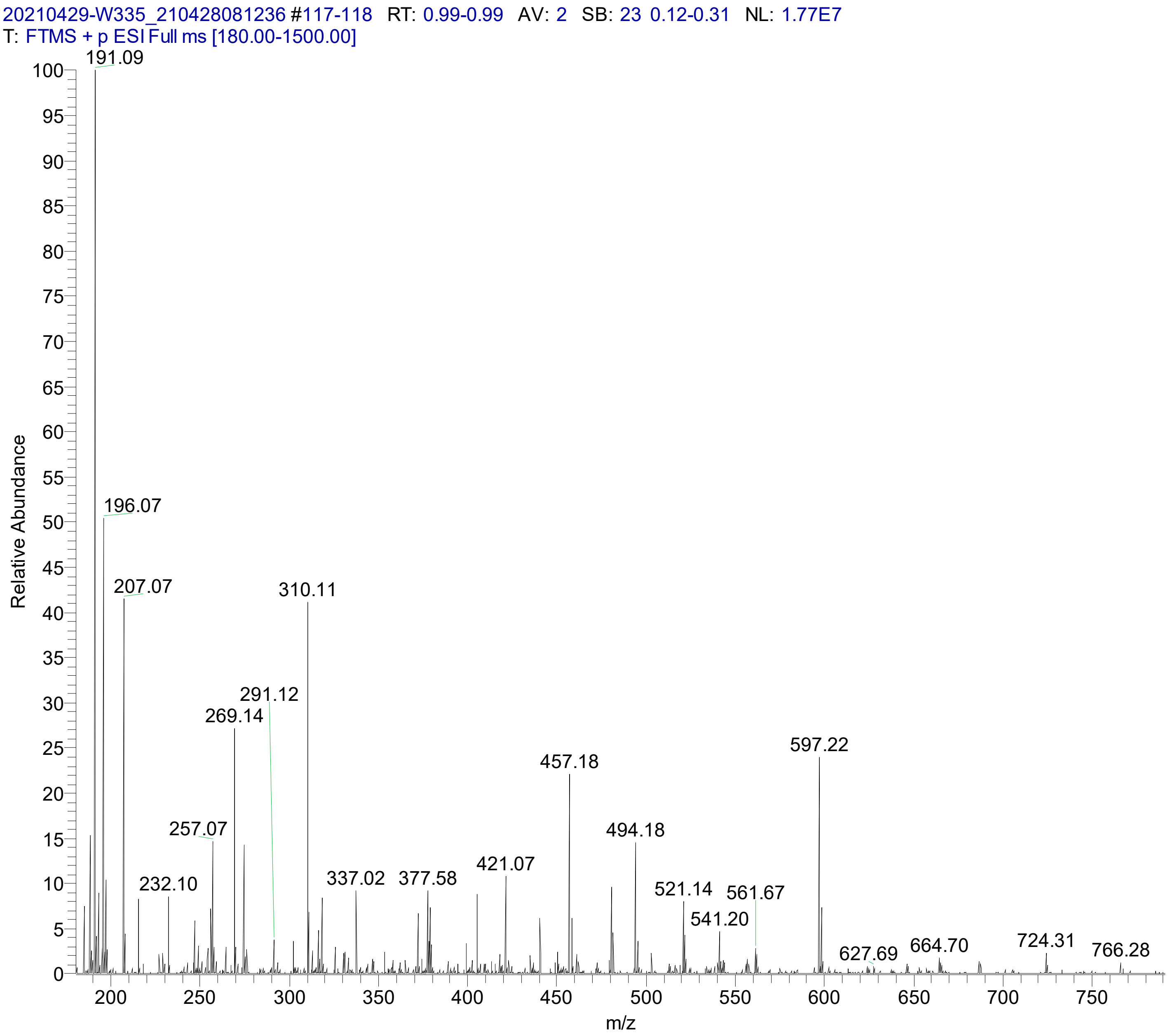

Figure S49. ESIMS spectrum of compound **11**.

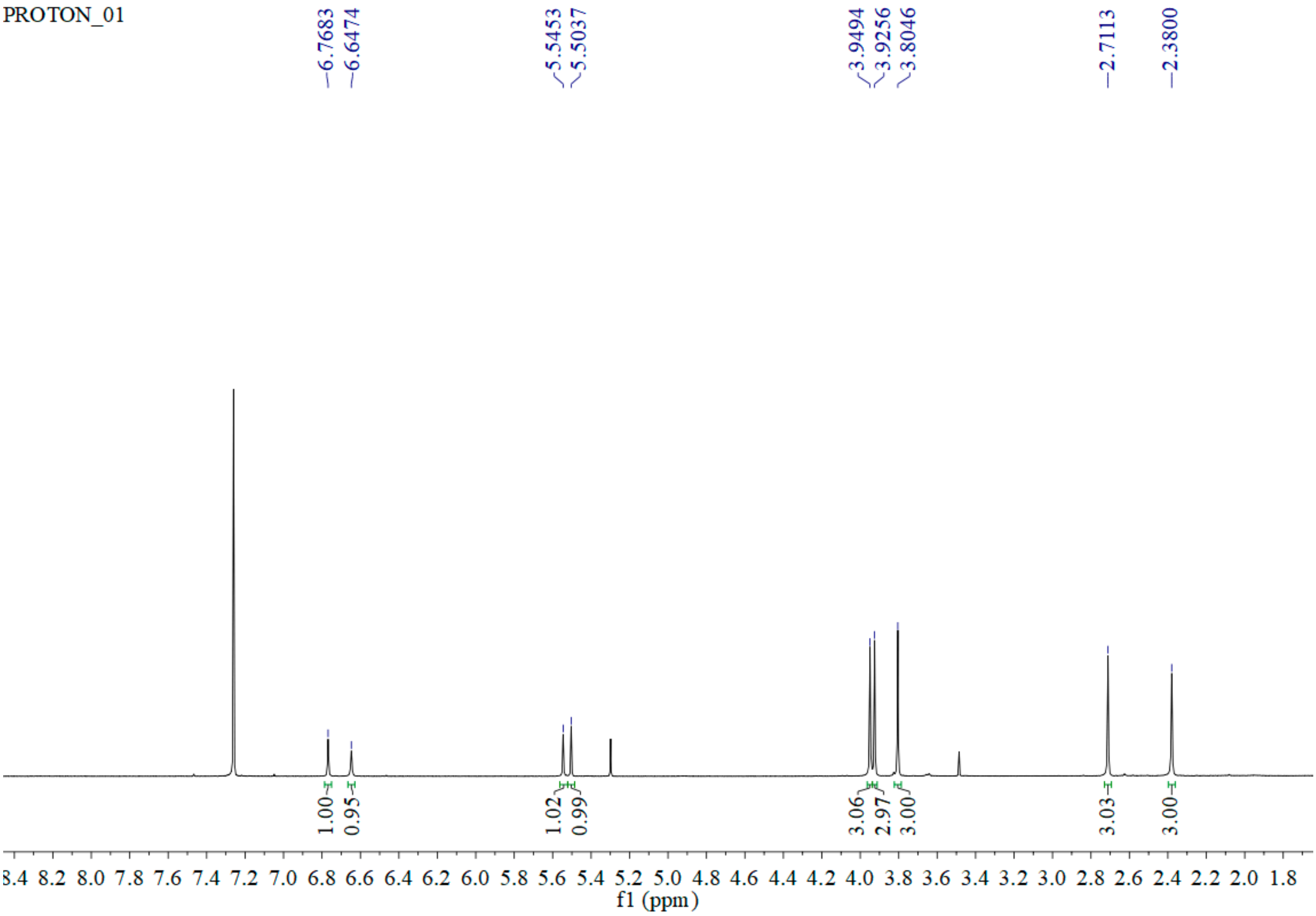

Figure S50. ^{1}H NMR spectrum (500 MHz) of compound **12** in $CDCl_3$.

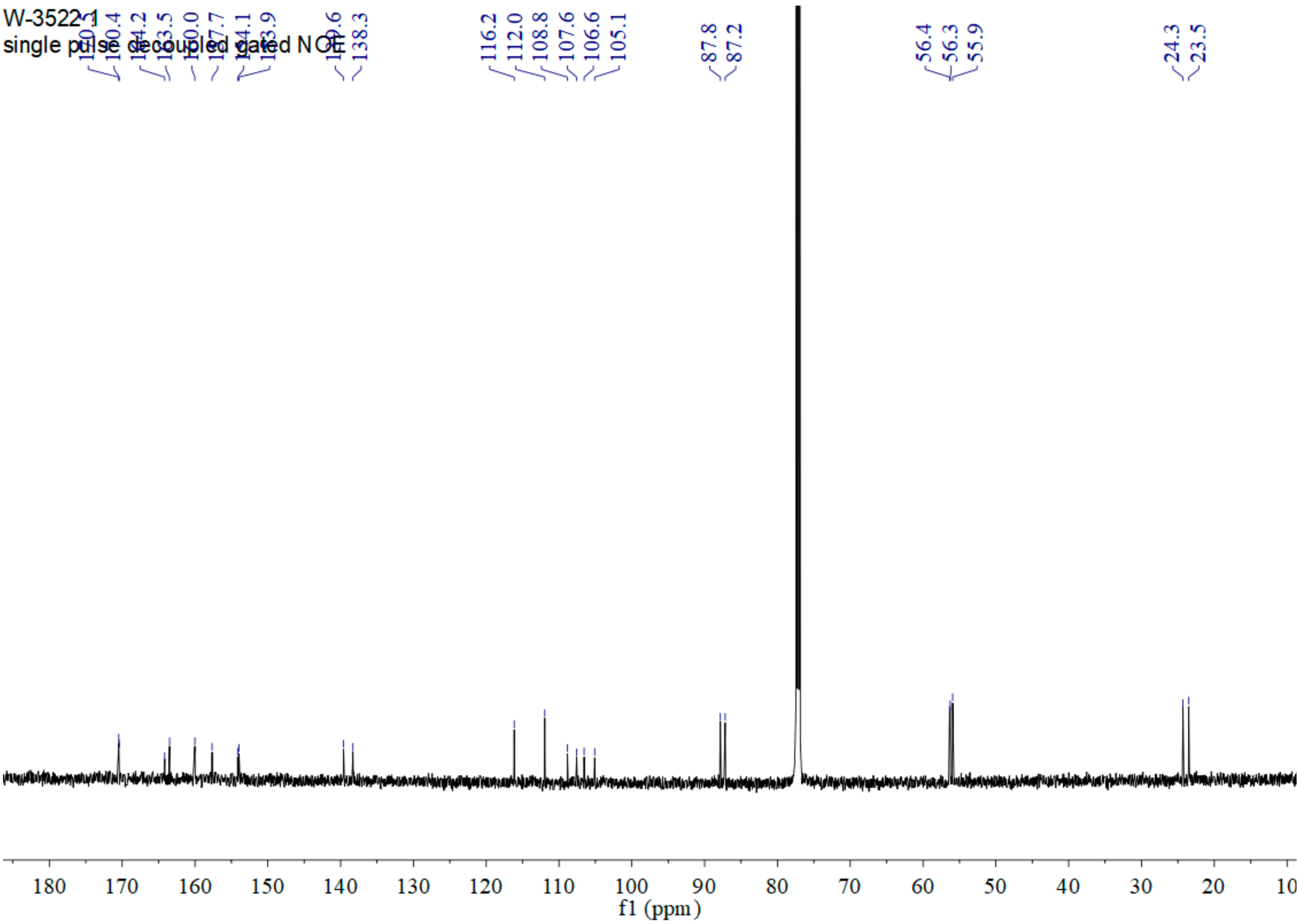

Figure S51. ^{13}C NMR spectrum (125 MHz) of compound **12** in $CDCl_3$.

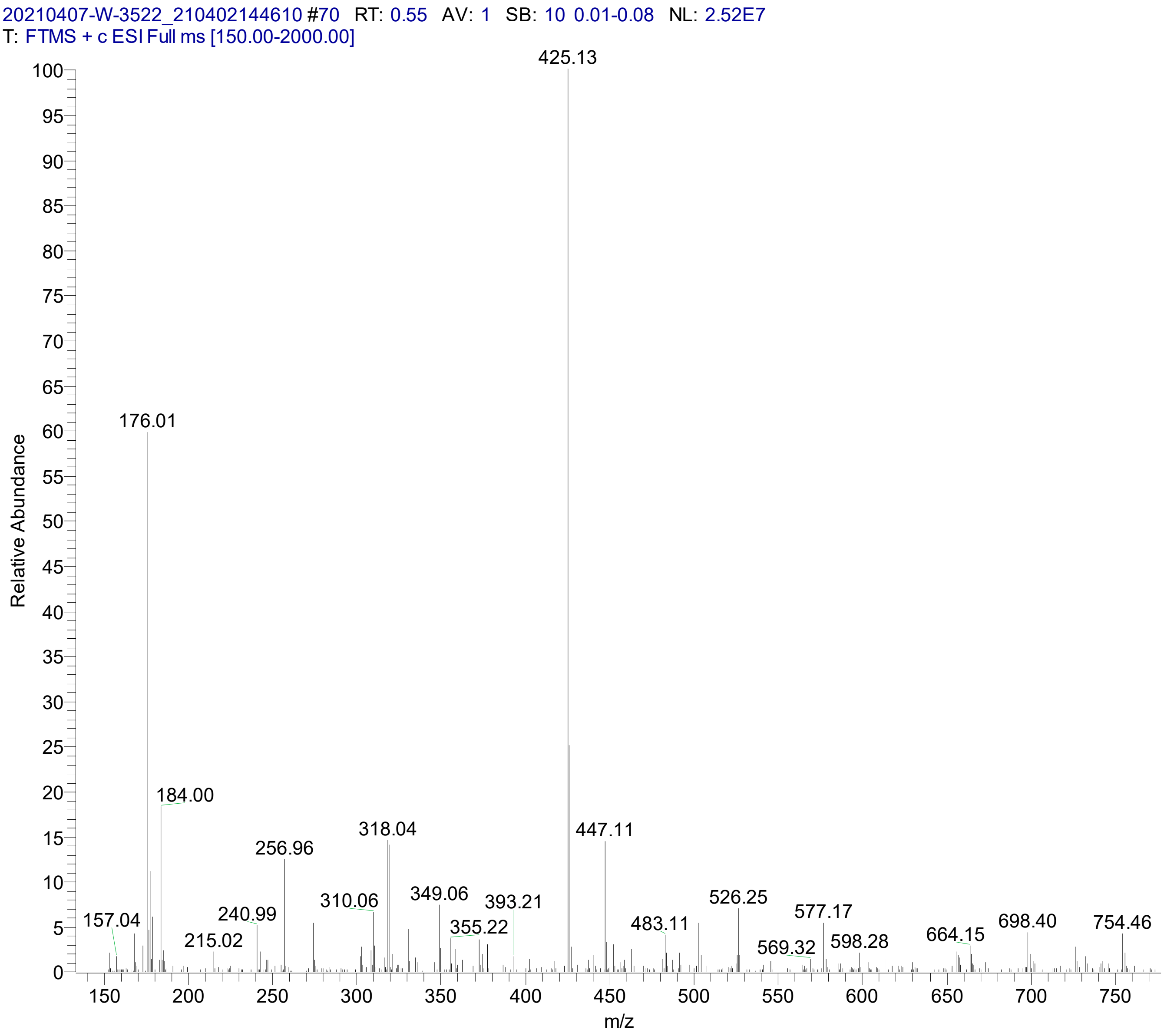

Figure S52. ESIMS spectrum of compound **12**.

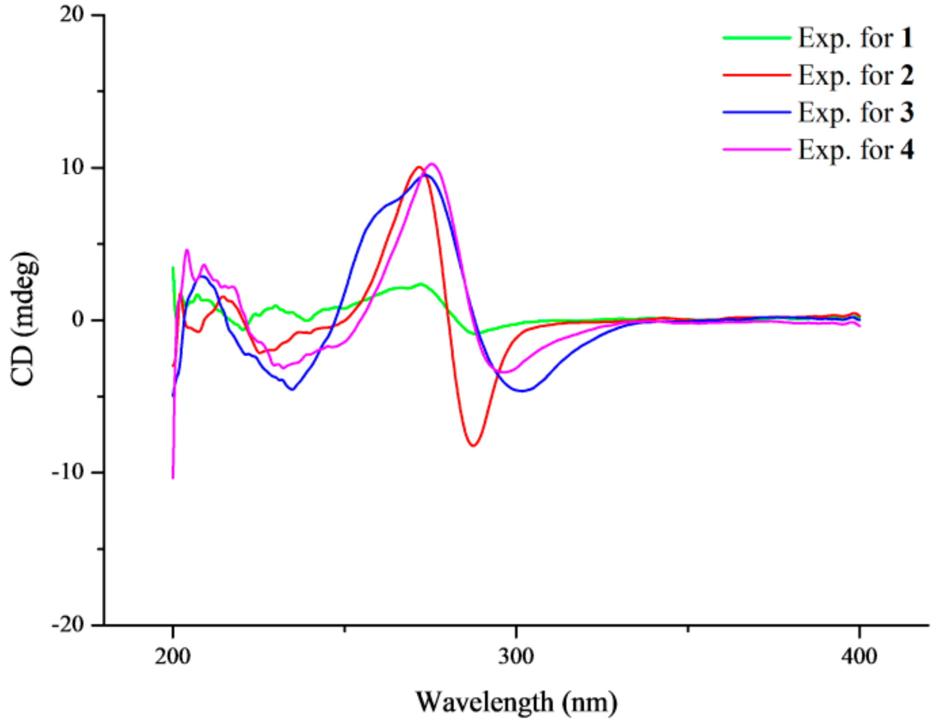

Figure S53. Experimental ECD spectra of compound **1**-**4**.

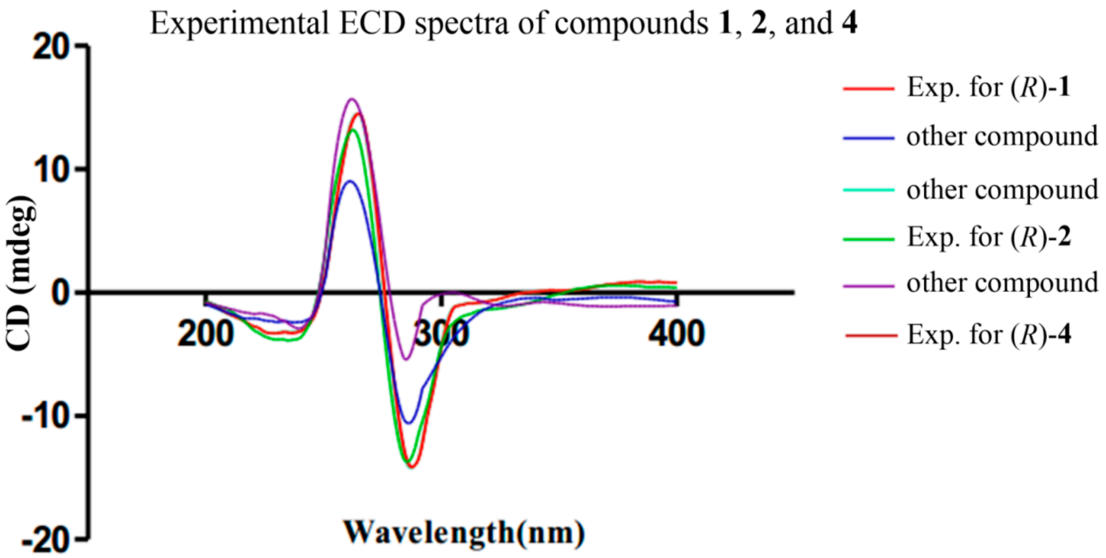

Figure S54. Experimental ECD spectra of compounds **1**, **2**, and **4** in the previously reported data.It was experimental ECD spectra of compounds **1**, **2**, and **4** in the previously reported data [S1].

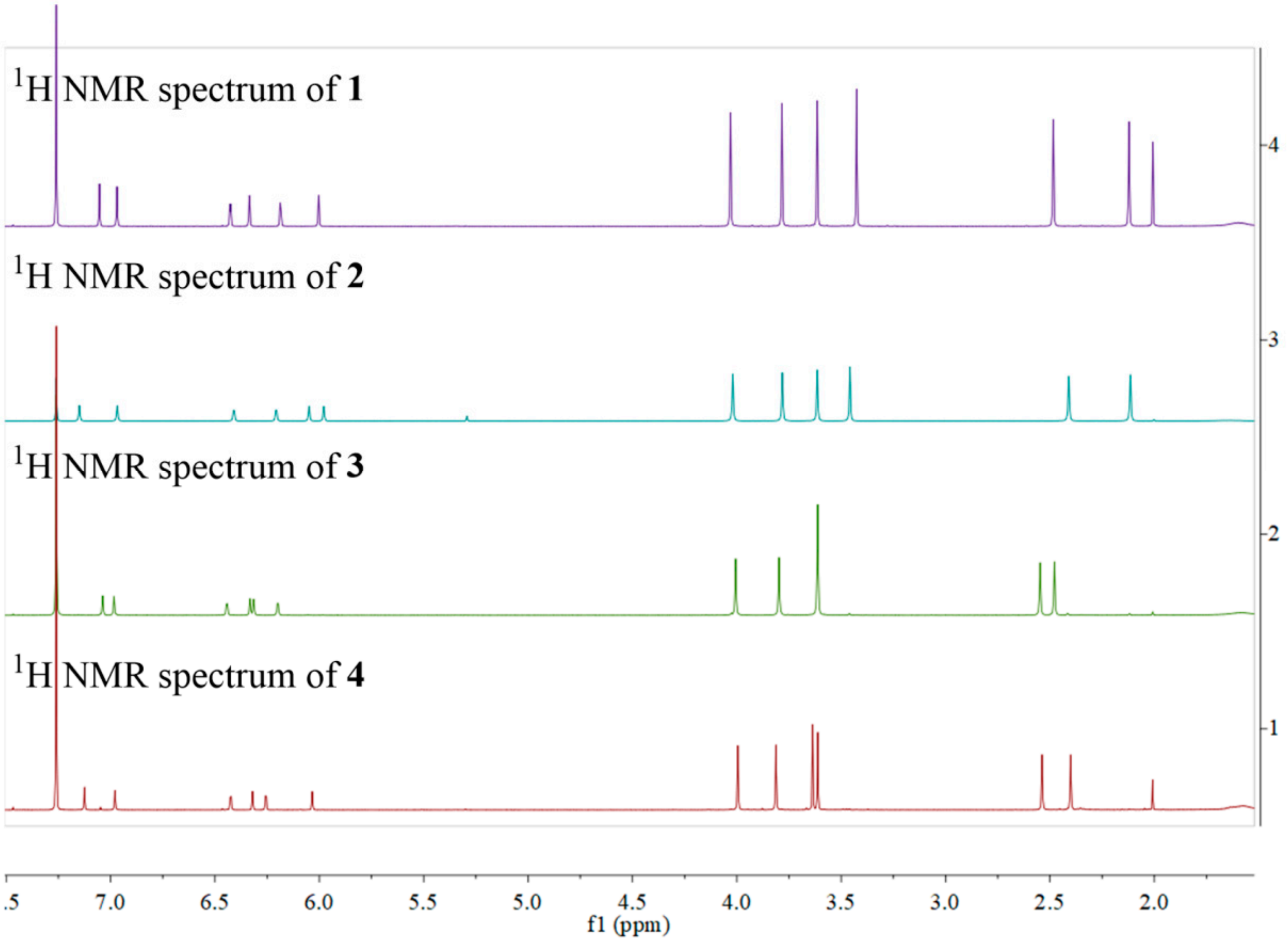

Figure S55. Comparison of ^{1}H NMR spectrums (500 MHz) of compounds **1-4** in $CDCl_3$.

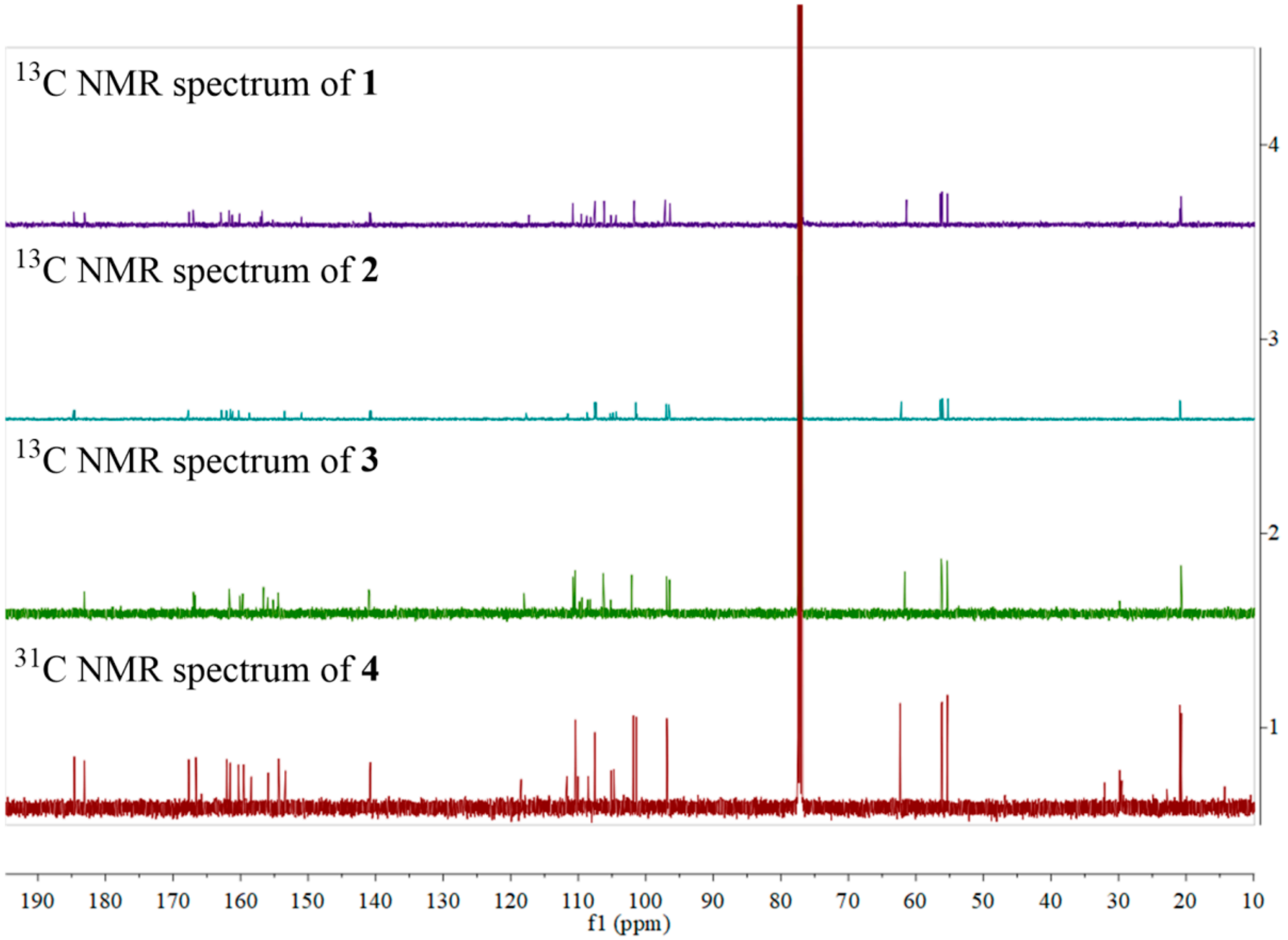

Figure S56. Comparison of ^{13}C NMR spectrums (125 MHz) of compounds **1**-**4** in $CDCl_3$.

Compound 5 ^{1}H NMR (600 MHz, $CDCl_3$) δ 6.71 (1H, s, H-7), 6.41 (1H, d, *J* = 2.6 Hz, H-7'), 6.34 (1H, s, H-10), 6.04 (1H, d, *J* = 2.7 Hz, H-9'), 5.98 (1H, s, H-3), 5.94 (1H, s, H-3'), 4.17 (3H, s, OCH_3-6), 4.03 (3H, s, OCH_3-6'), 3.81 (3H, s, OCH_3-8), 3.50 (3H, s, OCH_3-8'), 2.22 (3H, s, H_3-11), 2.03 (3H, s, H_3-11'). ^{13}C NMR (150 MHz, $CDCl_3$) δ 184.8 (C-4'), 184.4 (C-4), 167.9 (C-2), 167.8 (C-2'), 163.5 (C-5), 162.9 (C-5'), 161.8 (C-8'), 161.7 (C-6), 161.4 (C-6'), 159.5 (C-8), 153.5 (C-10a), 151.3 (C-10'a), 141.0 (C-9'a), 140.2 (C-9a), 108.9 (C-5'a), 108.8 (C-5a), 108.7 (C-9), 107.4 (C-3'), 107.3 (C-3), 107.0 (C-10'), 104.6 (C-4'a), 104.3 (C-4a), 99.2 (C-10), 97.2 (C-7'), 96.5 (C-9'), 92.8 (C-7), 56.5 (OCH_3-8), 56.4 (OCH_3-6'), 56.3 (OCH_3-6), 55.3 (OCH_3-8'), 20.8 (C-11'), 20.7 (C-11).

Compound **6** ^{1}H NMR (500 MHz, DMSO-d_6) δ 7.17 (1H, s, H-9), 6.80 (1H, s, H-10), 6.53 (1H, s, H-7'), 6.19 (1H, s, H-9'), 6.13 (1H, s, H-3'), 3.92 (3H, s, OCH_3-6'), 3.73 (3H, s, OCH_3-8), 3.58 (3H, s, OCH_3-8'), 3.33 (3H, s, OCH_3-6), 3.26 (1H, d, *J* = 17.0 Hz, H_a-3), 2.81 (1H, d, *J* = 17.0 Hz, H_b-3), 2.13 (3H, s, H_3-11'), 1.67 (3H, s, H_3-11). ^{13}C NMR (125 MHz, DMSO-d_6) δ 198.8 (C-4), 184.0 (C-4'), 168.6 (C-2'), 162.6 (C-5), 162.0 (C-5'), 161.6 (C-8'), 160.8 (C-6'), 160.2 (C-8), 158.5 (C-6), 153.9 (C-10a), 150.1 (C-10'a), 142.5 (C-9a), 140.1 (C-9'a), 115.6 (C-7), 109.3 (C-5'a), 107.6 (C-5a), 106.8 (C-3'), 105.2 (C-9), 103.6 (C-10'), 103.3 (C-10), 102.5 (C-4'a), 102.2 (C-4a), 100.5 (C-2), 96.6 (C-9'), 96.4 (C-7'), 61.4 (OCH_3-6), 56.0 (OCH_3-6'), 55.1 (OCH_3-8'), 55.1 (OCH_3-8), 47.9 (C-3), 27.6 (C-11), 20.2 (C-11').

Compound **7** ^{1}H NMR (500 MHz, DMSO-d_6) δ 12.93 (1H, s, OH-5'), 7.24 (1H, s, H-6'), 7.09 (1H, s, H-7'), 6.52 (1H, s, H-7), 6.45 (1H, s, H-3'), 5.93 (1H, s, H-9), 3.90 (3H, s, OCH_3-6), 3.75 (3H, s, OCH_3-8'), 3.54 (3H, s, OCH_3-10'), 3.47 (3H, s, OCH_3-8), 3.08 (1H, m, H_a-3), 2.86 (1H, m, H_b-3), 2.48 (3H, s, H_3-11'), 1.38 (3H, s, H_3-11). ^{13}C NMR (125 MHz, DMSO-d_6) δ 198.4 (C-4), 182.5 (C-4'), 168.2 (C-2'), 161.8 (C-8), 161.4 (C-6), 160.8 (C-8'), 160.0 (C-10'), 156.2 (C-5'), 155.6 (C-5), 154.4 (C-10'b), 151.5 (C-10a), 142.3 (C-6'a), 140.0 (C-9a), 118.2 (C-9'), 110.0 (C-10'a), 108.4 (C-5a), 107.4 (C-3'), 106.1 (C-4'a), 105.4 (C-10), 101.8 (C-4a), 100.4 (C-6'), 100.2 (C-2), 97.7 (C-7'), 96.6 (C-7), 95.2 (C-9), 60.9 (OCH_3-10'), 56.1 (OCH_3-6), 55.9 (OCH_3-8), 54.9 (OCH_3-8'), 48.1 (C-3), 27.7 (C-11), 20.0 (C-11') .

Compound 8 ^{1}H NMR (500 MHz, $CDCl_3$) δ 12.80 (1H, s, OH-5), 6.86 (1H, s, H-6), 6.57 (1H, s, H-7), 6.39 (1H, s, H-9), 6.26 (1H, s, H-3), 3.96 (3H, s, OCH_3-8), 3.92 (3H, s, OCH_3-10), 2.49 (3H, s, H_3-11). ^{13}C NMR (125 MHz, $CDCl_3$) δ 183.0 (C-4), 166.7 (C-2), 161.5 (C-10), 159.2 (C-8), 156.7 (C-5), 155.9 (C-10b), 141.3 (C-6a), 110.4 (C-3), 108.9 (C-4a), 105.9 (C-6), 105.0 (C-10a), 98.0 (C-7), 97.1 (C-9), 56.0 (OCH_3-8), 55.6 (OCH_3-10), 20.6 (C-11).

Compound **9** ^{1}H NMR (500 MHz, DMSO-d_6) δ 10.24 (1H, s, OH-7), 6.73 (1H, s, H-6), 5.57 (1H, s, H-3), 3.94 (3H, s, OCH_3-4), 2.60 (3H, s, H_3-9). ^{13}C NMR (125 MHz, DMSO-d_6) δ 169.7 (C-4), 161.8 (C-2), 158.6 (C-8a), 154.0 (C-7), 137.1 (C-5), 115.6 (C-6), 106.2 (C-4a), 105.8 (C-8), 86.5 (C-3), 56.6 (OCH_3-4), 23.3 (C-9).

Compound **10** ^{1}H NMR (600 MHz, DMSO-d_6) δ 6.60 (1H, s, H-6), 6.57 (1H, s, H-8), 5.58 (1H, s, H-3), 3.92 (3H, s, OCH_3-4), 3.75 (3H, s, OCH_3-7), 2.45 (3H, s, H_3-9). ^{13}C NMR (150 MHz, DMSO-d_6) δ 169.6 (C-4), 163.0 (C-7), 161.8 (C-2), 157.7 (C-8a), 136.4 (C-5), 115.4 (C-6), 106.3 (C-4a), 103.6 (C-8), 86.5 (C-3), 56.4 (OCH_3-4), 56.0 (OCH_3-7), 22.4 (C-9).

Compound **11** ^{1}H NMR (500 MHz, DMSO-d_6) δ 10.49 (1H, s, OH-7), 6.57 (1H, d, *J* = 2.4 Hz, H-6), 6.54 (1H, d, *J* = 2.4 Hz, H-8), 5.59 (1H, s, H-3), 3.92 (3H, s, OCH_3-4), 2.53 (3H, s, H_3-9). ^{13}C NMR (125 MHz, DMSO-d_6) δ 169.4 (C-4), 161.8 (C-2), 160.4 (C-7), 156.2 (C-8a), 138.2 (C-5), 116.0 (C-6), 106.1 (C-4a), 100.5 (C-8), 86.7 (C-3), 56.6 (OCH_3-4), 23.1 (C-9).

Compound **12** ^{1}H NMR (500 MHz, $CDCl_3$) δ 6.77 (1H, s, H-6), 6.65 (1H, s, H-6'), 5.55 (1H, s, H-3), 5.50 (1H, s, H-3'), 3.95 (3H, s, OCH_3-4), 3.93 (3H, s, OCH_3-4'), 3.80 (3H, s, OCH_3-7), 2.71 (3H, s, H_3-9), 2.38 (3H, s, H_3-9'). ^{13}C NMR (125 MHz, $CDCl_3$) δ 170.5 (C-4), 170.4 (C-4'), 164.2 (C-2), 163.5 (C-2'), 160.0 (C-7), 157.7 (C-7'), 154.1 (C-8'a), 153.9 (C-8a), 139.6 (C-5), 138.3 (C-5'), 116.2 (C-6'), 112.0 (C-6), 108.8 (C-4a), 107.6 (C-4'a), 106.6 (C-8), 105.1 (C-8'), 87.8 (C-3), 87.2 (C-3'), 56.4 (OCH_3-4), 56.3 (OCH_3-4'), 55.9 (OCH_3-7), 24.3 (C-9), 23.5 (C-9').

Figure S57. ^{1}H and ^{13}C NMR data of compounds **5**-**12**.

Table S1. ^{1}H NMR (600 MHz) and ^{13}C NMR (150 MHz) data of compound **13** in DMSO-d_6.

position	δ_C, type	δ_H, (J in Hz)	HMBC
2	168.1, C_q		
3	109.9, CH	6.52 (1H, s)	2, 4a, 11
4	182.5, C_q		
4a	108.3, C_q		
5	155.4, C_q		
6	105.3, CH	7.10 (1H, s)	4a, 7
6a	139.9, C_q		
7	101.7, CH	7.24 (1H, s)	6, 8, 9, 10a
8	160.3, C_q		
9	119.7, C_q		
10	156.1, C_q		
10a	107.4, C_q		
10b	154.4, C_q		
11	19.9, CH_3	2.48 (3H, s)	2, 3
3'	148.5, C_q		
4'	105.9, CH	6.93 (1H, s)	3', 5'
5'	138.7, C_q		
6'	101.9, C_q		
7'	154.1, C_q		
8'	105.1, C_q		
8'a	135.8, C_q		
9'	96.8, CH	5.96 (1H, d, J = 2.5)	11', 12'a
10'	157.9, C_q		
11'	94.5, CH	6.47 (1H, d, J = 2.6)	9', 12'a
12'	157.8, C_q		
12'a	104.5, C_q		
13'	152.8, C_q		
14'	10.3, CH_3	2.35 (3H, s)	4', 5'
OCH_3-8	55.8, CH_3	3.73 (3H, s)	8
OCH_3-10	60.6, CH_3	3.44 (3H, s)	10
OCH_3-10'	54.9, CH_3	3.51 (3H, s)	10'
OCH_3-12'	56.7, CH_3	4.09 (3H, s)	12'
NH-1'		12.98 (1H, s)	
OH-5		12.92 (1H, s)	4a, 6
OH-7'		12.47 (1H, s)	8'
OH-13'		10.36 (1H, s)	6', 12'a

Table S2. ^{1}H NMR (600 MHz) and ^{13}C NMR (150 MHz) data of compound **14** in DMSO-d_6.

position	δ_C, type	δ_H, (J in Hz)	HMBC
2	169.0, C_q		
3	106.8, CH	6.25 (1H, s)	2, 4a, 11
4	184.1, C_q		
4a	103.8, C_q		
5	160.8, C_q		
5a	110.7, C_q		
6	157.3, C_q		
7	120.1, C_q		
8	160.4, C_q		
9	101.8, CH	7.27 (1H, s)	5a, 7, 8, 10
9a	139.9, C_q		
10	101.3, CH	7.37 (1H, s)	4, 4a, 5a, 9
10a	152.3, C_q		
11	20.3, CH_3	2.44 (3H, s)	2, 3
3'	148.6, C_q		
4'	105.9, CH	6.92 (1H, s)	3', 5'
5'	138.7, C_q		
6'	101.9, C_q		
7'	154.1, C_q		
8'	105.2, C_q		
8'a	135.8, C_q		
9'	96.7, CH	5.96 (1H, d, J = 2.2)	11', 12'a
10'	157.8, C_q		
11'	94.6, CH	6.46 (1H, d, J = 2.2)	9', 12'a
12'	157.8, C_q		
12'a	104.6, C_q		
13'	152.7, C_q		
14'	10.3, CH_3	2.34 (3H, s)	3', 4', 5'
OCH_3-6	61.1, CH_3	3.41 (3H, s)	6
OCH_3-8	55.8, CH_3	3.73 (3H, s)	8
OCH_3-10'	54.8, CH_3	3.50 (3H, s)	10'
OCH_3-12'	56.7, CH_3	4.08 (3H, s)	12'
NH-1'		12.98 (1H, s)	5'
OH-7'		12.46 (1H, s)	8'
OH-13'		10.35 (1H, s)	6', 12'a, 13'

Table S3. ^{1}H NMR (500 MHz) data of compounds **1**-**4** in $CDCl_3$.

position	1	2	3	4
3	6.33 (1H, s)	6.05 (1H, s)	6.31 (1H, s)	6.03 (1H, s)
6	7.05 (1H, s)		7.04 (1H, s)	
7	6.97 (1H, s)		6.98 (1H, s)	
9		6.97 (1H, s)		6.98 (1H, s)
10		7.15 (1H, s)		7.12 (1H, s)
11	2.48 (3H, s)	2.41 (3H, s)	2.48 (3H, s)	2.40 (3H, s)
3′	6.00 (1H, s)	5.98 (1H, s)	6.33 (1H, s)	6.32 (1H, s)
7′	6.43 (1H, d, *J* = 2.2 Hz)	6.41 (1H, d, *J* = 2.3 Hz)	6.20 (1H, d, *J* = 2.2 Hz)	6.26 (1H, d, *J* = 2.2 Hz)
9′	6.19 (1H, d, *J* = 2.2 Hz)	6.21 (1H, d, *J* = 2.2 Hz)	6.44 (1H, d, *J* = 2.3 Hz)	6.42 (1H, d, *J* = 2.3 Hz)
11′	2.12 (3H, s)	2.11 (3H, s)	2.55 (3H, s)	2.54 (3H, s)
OCH_3-6		3.46 (3H, s)		3.64 (3H, s)
OCH_3-8	3.78 (3H, s)	3.78 (3H, s)	3.80 (3H, s)	3.81 (3H, s)
OCH_3-10	3.43 (3H, s)		3.61 (3H, s)	
OCH_3-6'	4.03 (3H, s)	4.02 (3H, s)		
OCH_3-8'	3.61 (3H, s)	3.61 (3H, s)	3.61 (3H, s)	3.61 (3H, s)
OCH_3-10'			4.01 (3H, s)	3.99 (3H, s)

Table S4. ^{13}C NMR (125 MHz) data of compounds **1**-**4** in $CDCl_3$.

position	1	2	3	4
2	167.0, C_q	167.8, C_q	167.0, C_q	167.7, C_q
3	110.8, CH	107.6, CH	110.8, CH	107.5, CH
4	183.1, C_q	184.6, C_q	183.1, C_q	184.6, C_q
4a	109.5, C_q	104.8, C_q	109.5, C_q	104.7, C_q
5	156.8, C_q	162.1, C_q	156.6, C_q	162.1, C_q
5a		111.6, C_q		111.7, C_q
6	106.2, CH	158.7, C_q	106.3, CH	158.4, C_q
6a	140.9, C_q		140.9, C_q	
7	101.7, CH	117.7, C_q	102.1, CH	118.5, C_q
8	160.2, C_q	160.3, C_q	160.1, C_q	160.3, C_q
9	117.3, C_q	101.5, CH	118.0, C_q	101.8, CH
9a		140.8, C_q		140.8, C_q
10	157.0, C_q	101.4, CH	156.7, C_q	101.4, CH
10a	108.1, C_q	153.5, C_q	108.2, C_q	153.3, C_q
10b	155.2, C_q		155.2, C_q	
11	20.7, CH_3	20.9, CH_3	20.7, CH_3	20.9, CH_3
2'	167.6, C_q	167.7, C_q	166.7, C_q	166.6, C_q
3'	107.5, CH	107.4, CH	110.5, CH	110.4, CH
4'	184.7, C_q	184.7, C_q	183.1, C_q	183.1, C_q
4'a	104.4, C_q	104.4, C_q	108.6, C_q	108.5, C_q
5'	162.9, C_q	162.8, C_q	154.4, C_q	154.3, C_q
5'a	108.7, C_q	108.7, C_q		
6'	161.2, C_q	161.2, C_q	109.8, C_q	110.0, C_q
6'a			141.0, C_q	140.9, C_q
7'	97.1, CH	97.0, CH	96.5, CH	96.8, CH
8'	161.7, C_q	161.5, C_q	161.7, C_q	161.6, C_q
9'	96.4, CH	96.6, CH	96.9, CH	96.9, CH
9'a	140.8, C_q	140.7, C_q		
10'	105.2, C_q	105.3, C_q	159.7, C_q	159.5, C_q
10'a	151.0, C_q	151.0, C_q	105.2, C_q	105.1, C_q
10'b			155.9, C_q	155.9, C_q
11'	20.9, CH_3	20.8, CH_3	20.7, CH_3	20.7, CH_3
OCH_3-6		62.2, CH_3		62.3, CH_3
OCH_3-8	56.1, CH_3	56.1, CH_3	56.3, CH_3	56.2, CH_3
OCH_3-10	61.4, CH_3		61.6, CH_3	
OCH_3-6'	56.4, CH_3	56.3, CH_3		
OCH_3-8'	55.3, CH_3	55.3, CH_3	55.4, CH_3	55.3, CH_3
OCH_3-10'			56.2, CH_3	56.1, CH_3

Table S5. Optical rotations of compounds **1**-**4** in MeOH at 20 °C.

compound	$[\alpha]^{20}_{D}$
1	-84.40 (*c* 0.0009, MeOH)
2	+4.17 (*c* 0.0016, MeOH)
3	+1.60 (*c* 0.0005, MeOH)
4	-28.10 (*c* 0.0005, MeOH)

References

(S1) He, Y.; Tian, J.; Chen, X.; Sun, W.; Zhu, H.; Li, Q.; Lei, L.; Yao, G.; Xue, Y.; Wang, J.; Li, H.; Zhang, Y., Fungal naphtho-γ-pyrones: Potent antibiotics for drug-resistant microbial pathogens. *Sci. Rep*. **2016**, *6*, 24291.
